# Image‐Based Morphological Profiling Identifies a Lysosomotropic, Iron‐Sequestering Autophagy Inhibitor

**DOI:** 10.1002/anie.201913712

**Published:** 2020-01-24

**Authors:** Luca Laraia, Guillaume Garivet, Daniel J. Foley, Nadine Kaiser, Sebastian Müller, Sarah Zinken, Thomas Pinkert, Julian Wilke, Dale Corkery, Axel Pahl, Sonja Sievers, Petra Janning, Christoph Arenz, Yaowen Wu, Raphaël Rodriguez, Herbert Waldmann

**Affiliations:** ^1^ Department of Chemical Biology Max-Planck-Institute of Molecular Physiology Otto-Hahn-Strasse 11 44227 Dortmund Germany; ^2^ Faculty of Chemistry and Chemical Biology TU Dortmund Otto-Hahn-Strasse 6 44227 Dortmund Germany; ^3^ Compound Management and Screening Center, Dortmund Otto-Hahn-Str. 11 44227 Dortmund Germany; ^4^ current address: Technical University of Denmark Department of Chemistry Kemitorvet 207 2800 Kgs. Lyngby Denmark; ^5^ current address: School of Physical and Chemical Sciences University of Canterbury Christchurch New Zealand; ^6^ Department of Chemistry Umeå Universitet KB.A4, Linnaeus väg 10 (rum: A4.35.07) 90187 Umeå Sweden; ^7^ Institut für Chemie der Humboldt-Universität zu Berlin Brook-Taylor-Str. 2 (R 1'102) 12489 Berlin Germany; ^8^ Institut Curie CNRS UMR 3666 INSERM U1143 PSL University Paris Chemical Cell Biology Group 26 Rue d'Ulm 75248 Paris Cedex 05 France

**Keywords:** autophagy, cell painting, lysosome, proteomics, target identification

## Abstract

Chemical proteomics is widely applied in small‐molecule target identification. However, in general it does not identify non‐protein small‐molecule targets, and thus, alternative methods for target identification are in high demand. We report the discovery of the autophagy inhibitor autoquin and the identification of its molecular mode of action using image‐based morphological profiling in the cell painting assay. A compound‐induced fingerprint representing changes in 579 cellular parameters revealed that autoquin accumulates in lysosomes and inhibits their fusion with autophagosomes. In addition, autoquin sequesters Fe^2+^ in lysosomes, resulting in an increase of lysosomal reactive oxygen species and ultimately cell death. Such a mechanism of action would have been challenging to unravel by current methods. This work demonstrates the potential of the cell painting assay to deconvolute modes of action of small molecules, warranting wider application in chemical biology.

## Graphical abstract

**I heard you paint cells**: Morphological profiling using the “cell painting” assay revealed that autoquin impairs lysosomal integrity, iron homeostasis, and autophagy.

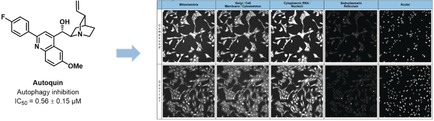

## Introduction

The identification of small molecules to probe biological systems is at the heart of chemical biology. Target‐agnostic phenotypic screens represent a rapid way to identify bioactive small molecules in physiologically relevant systems.^1^ However, a major challenge with this approach is the subsequent elucidation of molecular modes of action (MMOA) and target identification (ID) of bioactive compounds.^2^ Widely employed target ID techniques include affinity‐based pull‐down using immobilised derivatives followed by mass spectrometric protein identification.^3^ This typically requires the synthesis of a suitably functionalised probe, which might be time consuming or even infeasible if the compound of interest is highly complex. Emerging target identification strategies, which do not rely on modifications of the hit compound, such as thermal proteome profiling, are powerful additions to the target ID toolkit.^4^ However, these techniques are restricted to small molecules that mediate their activity through the targeting of proteins. In contrast, various drug classes target DNA,^5^ RNA,^6^ and lipids,^7^ and the discovery of regulatory RNA‐targeting small molecules has recently emerged as a new field.^8^ Therefore, the development of new methods, which enable the delineation of bioactive‐small‐molecule modes of action not mediated by binding to a protein target, is in high demand.

Morphological profiling has recently emerged as a complementary strategy for small‐molecule‐target identification. Monitoring changes in cellular morphology induced by a hit molecule and comparing these to changes induced by a set of reference compounds with known modes of action and targets can provide target hypotheses. Morphological profiles can be extracted from simple brightfield images,^9^ and obtained from complex fluorescence‐based high‐content screens in which multiple subcellular compartments are labelled with various fluorophores.^10^ The multiplexed use of different fluorophores has been established in the “cell painting” assay,^10^, ^11^ and has been proposed as a new strategy for determining whether a compound displays bioactivity in a very broad setting.^12^ In light of the promise and the potential of this approach, we explored the use of the cell painting assay for MMOA identification where other target identification methods had failed.^13^

**Scheme 1:**
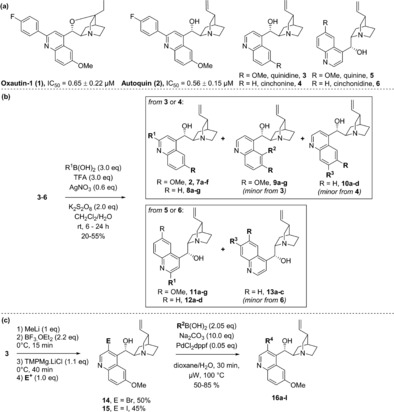
Synthesis of a cinchona alkaloid‐derived compound library. a) Molecular structures of previously identified autophagy inhibitor oxautin‐1, newly discovered inhibitor autoquin, and the four most abundant cinchona alkaloids. b) Synthesis of C2‐functionalised derivatives using the Borono–Minisci reaction. c) Synthesis of C3‐functionalised derivatives using selective C−H activation followed by Suzuki coupling. See Table 1 for details of the R‐groups investigated.

Recently we identified oxautin‐1 (**1**), a cinchona alkaloid‐derived autophagy inhibitor containing an oxazatwistane scaffold (Scheme 1 a).^14^ Autophagy is a cellular recycling process that degrades misfolded, aggregated, and/or superfluous proteins and organelles. The inhibition of autophagy is considered a potential anti‐cancer strategy^15^ making the identification of new small‐molecule autophagy inhibitors and their targets an intensive area of ongoing research.^14^, ^16^ Oxautin‐1 was predicted to inhibit both autophagosome biogenesis, and the fusion of autophagosomes and lysosomes, but its MMOA had remained elusive. Given this unknown mode of action, we embarked on the synthesis and biological investigation of more readily accessible and structurally more diverse oxautin analogues. We now report the discovery of the cinchona‐alkaloid‐derived autophagy inhibitor autoquin (**2,** Scheme 1 a). Analysis of morphological changes induced by autoquin in the cell painting assay unraveled that, like oxautin‐1, autoquin inhibits autophagy by indirect modulation of the activity of the lysosomal enzymes acid sphingomyelinase and acid ceramidase, resulting in impaired lysosome–autophagosome fusion. Deeper investigation revealed that autoquin also sequesters Fe^2+^ in lysosomes, which results in increased formation of lysosomal reactive oxygen species (ROS) and cell death.

## Results and Discussion

The oxautins were synthesised by intramolecular cyclisation of quinidine (**3**) and cinchonine (**4**) but respective analogues cannot be obtained from the cinchona alkaloids quinine (**5**) and cinchonidine (**6**) owing to their different configuration at the carbon atom α to the quinuclidine nitrogen atom, precluding cyclisation and limiting further exploration of oxazatwistane analogues as autophagy inhibitors (Scheme 1 a).^14^ In addition, the cyclisation to yield the oxazatwistane ring system in the oxautins requires somewhat harsh conditions and removes two vectors for further functionalisation, thereby further limiting exploration of SAR. Therefore, we investigated whether the oxazatwistane core was required for autophagy inhibitory activity. To this end, the four major cinchona alkaloids quinidine, quinine, cinchonine, and cinchonidine were subjected to Borono–Minisci conditions^17^ to selectively functionalise the C2 position and to evaluate the importance of the relative stereochemistry at the quinuclidine ring (Scheme 1 b). In addition to the expected C2‐functionalised derivatives, some reactions also delivered a minor product corresponding to the C5‐ (compounds **9**, from quinidine) or C7‐functionalised scaffold (**10**, from cinchonine, and **13**, from cinchonidine). In addition, functionalisation of the C3 position had not previously been investigated for the oxazatwistanes. Selective halogenation at C3^18^ provided intermediates that could be subjected to metal‐catalysed cross coupling reactions (Scheme 1 c, **14**–**15**). Suzuki reactions enabled the synthesis of 13 additional analogues (**16 a**–**l**).

The resulting 49‐membered compound collection was investigated for autophagy inhibition in MCF7 cells stably expressing EGFP‐tagged LC3, a widely used autophagy marker.^19^ Compounds that were able to reduce EGFP‐LC3 puncta formation upon autophagy induction by amino acid starvation using Earle's Balanced Salt Solution (EBSS) were classed as hits.^20^ The direct oxautin‐1^14^ analogue **2** derived from quinidine but lacking the oxazatwistane ring, displayed very similar potency in the autophagy assay, suggesting that the oxazatwistane ring was not essential for biological activity (Table 1, Entry 1). This compound, which we named autoquin, provided a benchmark against which all other compounds were assessed. Small variations at the C2 position resulted in a modest drop in activity (Table 1, Entries 2 and 3), while removal of the *p*‐F substituent completely abolished it (Entry 4). Varying the position of substitution on the phenyl ring reduced or abolished activity (Entries 5–7). Compounds with aryl substituents at C5 of the quinoline ring were generally inactive (Entries 8, 9, and 11) though a *m*‐Cl substituent returned some activity (Entry 10). In general, cinchonine derivatives, lacking the C6 methoxy group (Entries 12–21), were all less active than the quinidine‐derived compounds, confirming the importance of this residue for optimal activity. The *p*‐F‐C_6_H_4_ substituent at C2 retained the highest levels of activity (Entry 12), observed for all the quinidine‐derived compounds (Entry 1) and the oxazatwistanes.^14^ To assess the importance of the stereochemistry at the quinuclidine ring, a small collection of C2‐substituted derivatives of quinine and cinchonidine was synthesised and evaluated (Entries 22–36). Although three quinine‐derived analogues showed appreciable levels of activity, they were all at least 6‐fold less active than autoquin and were not pursued further. Crucially, the *p*‐F‐Ph substituted quinine and cinchonidine analogues (Entries 22 and 29) were significantly less active than their quinidine and cinchonine‐derived diastereomers (Entries 1 and 12). All compounds with substituents at the C3 position on the quinoline ring were either less active than autoquin (Entries 39 and 40) or completely inactive (Entries 37, 38, and 41–49).

**Table 1:** Structure activity relationships of cinchona alkaloid‐derived autophagy inhibitors. IC_50_ data represents the ability to inhibit autophagy induced by amino acid starvation using EBSS and is mean ± SD of three independent experiments. **2**=Autoquin. 
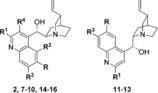

Entry	#	R	R^1^	R^2^	R^3^	R^4^	IC_50_ [μm]
1	**2**	OMe	*p*‐F‐C_6_H_4_	H	H	H	0.56±0.15
2	**7 a**	OMe	*p*‐Cl‐C_6_H_4_	H	H	H	2.44±1.01
3	**7 b**	OMe	*p*‐Me‐C_6_H_4_	H	H	H	2.31±0.07
4	**7 c**	OMe	Ph	H	H	H	>10
5	**7 d**	OMe	*m*‐Br‐C_6_H_4_	H	H	H	7.48±0.70
6	**7 e**	OMe	*m*‐Cl‐C_6_H_4_	H	H	H	5.70±0.39
7	**7 f**	OMe	*m*‐Cl‐*p*‐F‐C_6_H_3_	H	H	H	>10
8	**9 a**	OMe	H	*p*‐F‐C_6_H_4_	H	H	>10
9	**9 b**	OMe	H	*m*‐Cl‐C_6_H_4_	H	H	>10
10	**9 c**	OMe	H	*m*‐Cl‐*p*‐F‐C_6_H_3_	H	H	5.40±2.40
11	**9 d**	OMe	H	Ph	H	H	>10
12	**8 a**	H	*p*‐F‐C_6_H_4_	H	H	H	1.62±0.19
13	**8 b**	H	*p*‐Cl‐C_6_H_4_	H	H	H	Toxic
14	**8 c**	H	*p*‐Me‐C_6_H_4_	H	H	H	>10
15	**8 d**	H	Ph	H	H	H	>10
16	**8 e**	H	*m*‐Br‐C_6_H_4_	H	H	H	>10
17	**8 f**	H	*m*‐Cl‐C_6_H_4_	H	H	H	5.43±0.13
18	**8 g**	H	*m*‐Cl‐*p*‐F‐C_6_H_4_	H	H	H	>10
19	**10 a**	H	H	H	*p*‐F‐C_6_H_4_	H	>10
20	**10 b**	H	H	H	*p*‐Cl‐C_6_H_4_	H	2.52±0.32
21	**10 c**	H	H	H	*m*‐Br‐C_6_H_4_	H	>10
22	**11 a**	OMe	*p*‐F‐C_6_H_4_	H	H	H	3.30±1.60
23	**11 b**	OMe	*p*‐Cl‐C_6_H_4_	H	H	H	2.70±1.30
24	**11 c**	OMe	*p*‐Me‐C_6_H_4_	H	H	H	>10
25	**11 d**	OMe	Ph	H	H	H	>10
26	**11 e**	OMe	*m*‐Br‐C_6_H_4_	H	H	H	2.72±1.31
27	**11 f**	OMe	*m*‐Cl‐C_6_H_4_	H	H	H	na
28	**11 g**	OMe	*m*‐Cl‐*p‐*F‐C_6_H_3_	H	H	H	na
29	**12 a**	H	*p*‐F‐C_6_H_4_	H	H	H	>10
30	**12 b**	H	*p*‐Cl‐C_6_H_4_	H	H	H	5.84±1.30
31	**12 c**	H	*p*‐Me‐C_6_H_4_	H	H	H	>10
32	**12 d**	H	Ph	H	H	H	>10
33	**12 e**	H	*m*‐Br‐C_6_H_4_	H	H	H	>10
34	**13 a**	H	H	H	*p*‐F‐C_6_H_4_	H	>10
35	**13 b**	H	H	H	*p*‐Cl‐C_6_H_4_	H	na
36	**13 c**	H	H	H	Ph	H	>10
37	**16 a**	OMe	H	H	H	*p*‐F‐C_6_H_4_	>10
38	**16 b**	OMe	H	H	H	*p*‐Cl‐C_6_H_4_	>10
39	**16 c**	OMe	H	H	H	*p*‐NO_2_‐C_6_H_4_	3.87±0.69
40	**16 d**	OMe	H	H	H	*p*‐CF_3_‐C_6_H_4_	3.52±0.69
41	**16 e**	OMe	H	H	H	*p*‐NHBoc‐C_6_H_4_	>10
42	**16 f**	OMe	H	H	H	4‐py	>10
43	**16 g**	OMe	H	H	H	*m*‐F‐C_6_H_4_	>10
44	**16 h**	OMe	H	H	H	*m*‐F‐*p‐*F‐C_6_H_4_	>10
45	**16 i**	OMe	H	H	H	3,5‐di‐F‐ C_6_H_4_	>10
46	**16 j**	OMe	H	H	H	*m*‐Cl‐*p‐*F‐C_6_H_4_	>10
47	**16 k**	OMe	H	H	H	3,4‐di‐OMe‐ C_6_H_4_	>10
48	**16 l**	OMe	H	H	H	3,5‐di‐OMe‐ C_6_H_4_	>10
49	**14**	OMe	H	H	H	‐Br	>10

**Figure 1:**
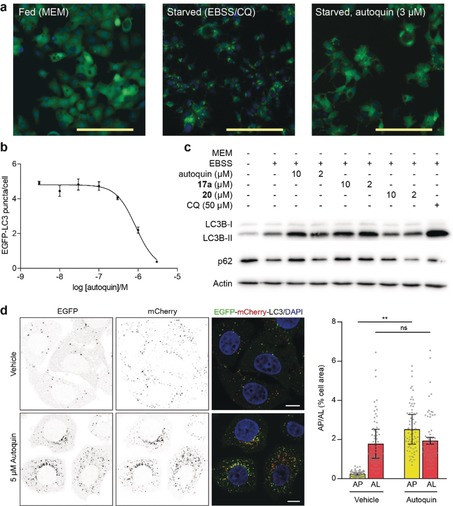
Validation of autoquin as an autophagy inhibitor. a) Effect of autoquin on MCF7 cells stably expressing EGFP‐LC3 upon autophagy induction by amino acid starvation using EBSS; *n*=3, representative images, scale bar=100 μm; CQ=chloroquine (50 μm). b) Quantification of the reduction in EGFP‐LC3 puncta by autoquin. Data points are mean ± SEM of three independent experiments. c) Effect of autoquin and analogues **17 a** and **20** (see Scheme 2 for structures and data) on LC3‐II and p62 levels as assessed by western blot; *n*=3, representative images shown. d) MCF7 cells stably expressing mCherry‐EGFP‐LC3 treated with vehicle or 5 μm autoquin for 24 h, scale bars=10 μm. e) Autophagosomes (AP; yellow puncta) and autolysosomes (AL; red puncta) from (d) were quantified and data represented as percentage of cell area. Bar graphs show mean ± SD from three biologically independent experiments. Data points represent individual cells pooled from the three independent experiments (*n*≥23 cells per replicate). Significance was determined from biological replicates using a two‐tailed, unpaired *t*‐test. ns=not significant, **p=0.0064.

Having established that the oxazatwistane scaffold was not essential for autophagy inhibition and that the *p*‐F‐C_6_H_4_ substituent at C2 of the quinoline ring was best for autophagy inhibition, we proceeded to validate autoquin as an autophagy inhibitor. As described above, autoquin showed a dose‐dependent inhibition of EGFP‐LC3 puncta after 3 hours upon autophagy induction by amino acid starvation in the primary screening assay (Figure 1 a,b). Additionally, it increased the stability of the chaperone p62 to autophagosome‐mediated degradation as assessed by western blot (Figure 1 c), suggesting that it is an inhibitor of autophagic flux. However, autoquin also showed a dose‐dependent increase in LC3‐II levels, suggesting that it is also an inhibitor of autophagosome maturation, similarly to oxautin‐1 (Figure 1 c). This finding was further strengthened by using a tandem mCherry‐EGFP‐LC3 expressing cell line, which enables the simultaneous monitoring of autophagosomes (green and red fluorescence) and autolysosomes (red fluorescence only). Exposing fed cells to autoquin for 24 hours resulted in a marked increase in autophagosomes, confirming its inhibitory effect on autophagosome maturation (Figure 1 d).

**Scheme 2:**
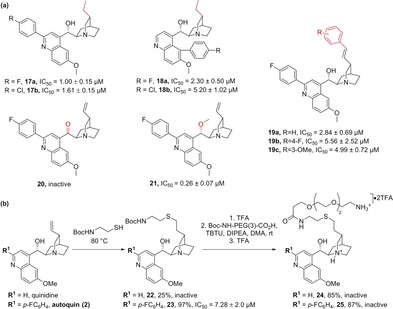
SAR of the quinuclidine ring and pull‐down probe synthesis. a) Autophagy inhibitory activity of analogues with variations vicinal to the quinuclidine ring, variations highlighted in red. b) Synthesis and autophagy inhibitory activities of pull‐down probes for target identification experiments.

Having validated autoquin as a bona fide autophagy inhibitor and confirmed its effect on autophagosome maturation, we focused our attention on making smaller, more targeted modifications to the autoquin scaffold to identify a suitable immobilisation point for affinity‐based target enrichment. Reduction of the alkene resulted in two derivatives with retained biological activity (**17 a**,**b**), suggesting that this may be a suitable position for further functionalisation (Scheme 2 a and Figure 1 c). C5‐substituted analogues (**18 a**,**b**) were less active, as observed with the unsaturated analogues (Scheme 2 a and Table 1). Arylation of the vinyl group via Heck coupling produced three derivatives (**19 a**–**c**) that, though less active than autoquin, retained good potency levels. Oxidation of the hydroxy group to a ketone led to an inactive compound (**20**) while, interestingly, methylation of the hydroxy group produced a very active compound (**21**), suggesting that this position is critical for potency. To access a derivative suitable for immobilisation and affinity‐based target enrichment, autoquin was subjected to an ene reaction with 2‐(Boc‐amino)ethanethiol to yield a compound (**23**), which was further elaborated into a pull‐down probe (**25,** Scheme 2 b). A corresponding negative probe (**24**) lacking the *p*‐fluorophenyl group was also synthesized from **22**. While the negative probe and its precursors were all inactive, the final positive probe **25** was as well. The phenomenon by which the introduction of an unprotected amino‐PEG linker leads to loss of activity has been observed by us on several occasions and can be ascribed to a presumable loss of cell permeability. In this case, the presence of two basic amines makes it more likely that a doubly charged molecule would interact with the cell membrane. As the pull‐down experiment was carried out in cell lysates and the intermediate **23** retained appreciable levels of activity, we continued with **25** as a positive probe.

To identify autoquin target proteins by affinity enrichment, the probes were immobilised on NHS‐activated magnetic beads and incubated with cell lysate. Proteins selectively enriched with the autoquin‐derived probe compared to the negative probe were considered to be hits. The only hit that was conclusively identified in all replicates was ferrochelatase (Supplementary Figure 1). Ferrochelatase (FECH) is located in the mitochondria and catalyses the insertion of iron into protoporphyrin IX, the last step in heme biosynthesis.^21^ No known link between FECH and autophagy has previously been reported. However, autophagy has been shown to play a role in mediating iron homeostasis through the selective degradation of ferritin.^22^ In an attempt to validate FECH as a target of autoquin, the pull‐down was repeated with non‐immobilised autoquin as a competitor and analysed by western blot. However, despite FECH enrichment by the positive probe (**25**) compared to the negative probe (**24**), no competition was observed (Supporting Information, Figure S2 a). Additionally, autoquin was not able to stabilise FECH to thermal denaturation as assessed by isothermal dose‐response fingerprinting, even at very high concentrations (Figures S2 b,c). Thus, FECH was not considered a direct functional target of autoquin.

Since chemical proteomics had not validated a target, autoquin was investigated in the multiparametric image‐based cell painting assay, which enables interrogation of a cellular system in its entirety.^10^, ^11^, ^12^, ^13^ Cell painting involves staining cells with markers for specific cellular compartments following compound treatment. A vast array of phenotypes including cell shape, morphology, size, and fluorescence intensity and distribution can be assessed simultaneously for each treatment condition, generating a set of fingerprints characteristic to a particular mode of action or target.^10^, ^11^, ^23^ The power of this assay becomes apparent when a library of reference compounds of known bioactivity is included in the screen and their fingerprints are compared with profiles recorded for novel compounds. In principle, this can enable the discovery of bioactivity profiles that are different, and thus novel, compared to a reference set, but also known modes of action can be revealed by similarity assessment.^13^

**Figure 2:**
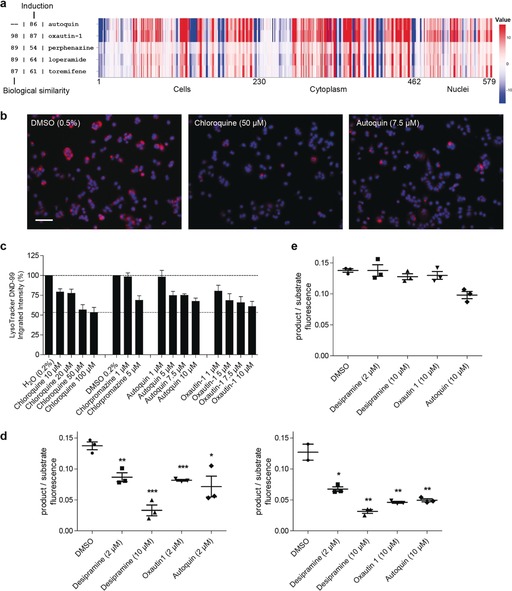
Autoquin is a lysosomotropic compound that acts as a functional inhibitor of acid sphingomyelinase. a) Cell painting profiles of autoquin and its most biosimilar compounds oxautin‐1, perphenazine, loperamide, and toremifene. See Figure S3 for representative images. b) Representative fluorescence microscopy images of MC7 cells treated with lysosomotropic compounds for 3 h and stained with Lysotracker DND‐99; scale bar=110 μm. c) Quantification of (b), *n*=3, data is mean ± SD. d) Product/substrate ratio of the acid sphingomyelinase (left) and acid ceramidase (right) reaction in intact cells. *n*=3, data is mean ± SD. e) Product/substrate ratio of the acid ceramidase reaction in cell lysates. *n*=3, data is mean ± SD. Statistical significance comparing treated samples to the DMSO control for (d) and (e) was assessed using the Student's *t*‐test. *p<0.05, **p<0.01, ***p<0.001.

Autoquin, oxautin‐1, and several analogues were characterised in the cell painting assay, in which their effect on 579 parameters (see the Supporting Information for the delineation of the parameters) was compared with the results obtained for a reference compound set comprising 3000 compounds with known bioactivity (see the Supporting Information for details). To assess the similarity in the bioactivity of the fingerprint profiles, “biological similarity” was employed (BioSim; see the Supporting Information for determination of similarity). Furthermore an “induction” value (the fraction of parameters (in %) that underwent significant changes (median absolute deviation (MAD) value upon compound treatment of at least +/− three‐fold of the median determined for the DMSO controls; see the Supporting Information)) was determined as measure for compound bioactivity. Compounds with an induction value of >10 % were considered bioactive in the cell painting assay. This analysis resulted in the discovery of three annotated compounds with high similarity (>80 %) in their bioactivity fingerprints to autoquin (Figure 2 a and Figure S3). Although at first glance, perphenazine (reportedly a non‐selective G‐protein‐coupled receptor ligand), loperamide (an opioid receptor agonist), and toremifene (an estrogen receptor ligand) do not display obvious biological or indeed chemical similarity, all three have been reported to be lysosomotropic compounds.^24^ Lysosomotropic compounds are typically hydrophobic with at least one basic nitrogen atom that, upon protonation, enables them to be trapped in the lysosomes.^25^ A comparison of their physicochemical properties revealed that all compounds are likely to be fully protonated at pH 4–5, typically found in the lysosome (Supporting Information, Table S1). This would favour a model in which they are able to pass cellular and lysosomal membranes before being protonated and trapped in the lysosome. A potential lysosomotropic profile had previously been suggested in the cell painting assay for structurally different compounds, but was not further investigated.11a To confirm whether autoquin and oxautin are indeed lysosomotropic, we assessed their ability to inhibit the accumulation of the lysosomal tracer Lysotracker Red (LR) DND‐99. A decrease in fluorescence intensity is often characteristic of a lysosomotropic phenotype, if the lysosomal pH is increased. Both autoquin and oxautin‐1 showed a dose‐dependent decrease in lysosomal accumulation of LR after a 3 hour treatment, similarly to the known lysosomotropes chloroquine and chlorpromazine (Figure 2 b,c). Lysosomotropic compounds are also often functional inhibitors of acid sphingomyelinase (FIASMAs), and other sphingolipid hydrolases including acid ceramidase.^26^ They do not directly interact with the hydrolases (therefore, they are qualified as functional inhibitors) but rather affect the inner lysosomal membrane localisation of acid sphingomyelinase and other sphingolipid hydrolases through a direct interaction with the negatively charged lipid bis(monoacylglycerol)phosphate (BMP), resulting in the degradation of the hydrolases.^27^ Both autoquin and oxautin‐1 were tested in a fluorescence‐based assay to monitor both acid sphingomyelinase and acid ceramidase activity in a cell‐based and a cell‐free system.^28^ FIASMAs characteristically inhibit hydrolase activity in cell‐based assays, in which intact lysosomes are present, but not in cell‐free systems. Both autoquin and oxautin‐1 inhibited acid sphingomyelinase and ceramidase activity in intact cells (Figure 2 d) but not in lysates (Figure 2 e), similarly to the control compound desipramine.

**Figure 3:**
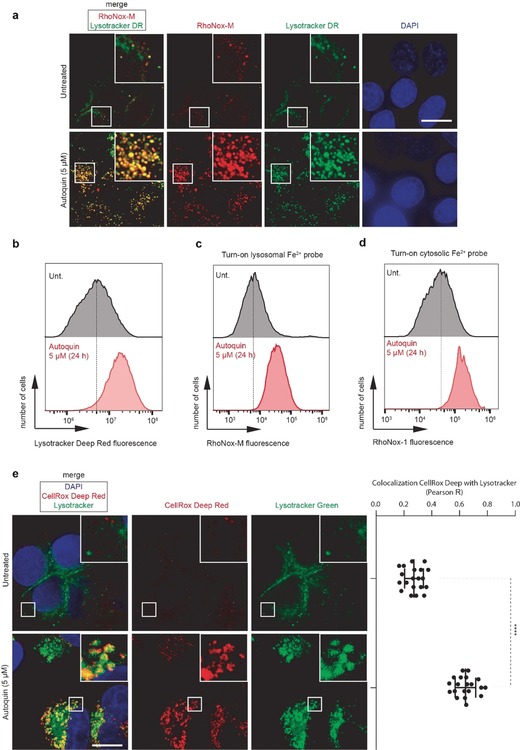
Autoquin increases lysosomal mass and sequesters Fe^2+^ to the lysosomes in MCF7 cells, causing an increase in lysosomal reactive oxygen species. a) Fluorescence microscopy images showing the subcellular localisation of Fe^2+^ (red) and lysosomes (green) by means of RhoNox‐M and Lysotracker deep red (DR) fluorescence, respectively. b) Flow cytometry quantification of Lysotracker DR intensity from cells treated according to (a). c) Flow cytometry quantification of RhoNox‐M intensity from cells treated according to (a). d) Flow cytometry quantification of intracellular Fe^2+^ using an alternative turn‐on fluorescent probe RhoNox‐1. e) Fluorescence microscopy images showing the subcellular localization of ROS (red) by means of fluorogenic reaction with CellROX deep red in MCF7 cells treated with autoquin (5 μm) for 24 h, scale bar=10 μm. Colocalization with Lysotracker DND‐26 (green) assessed by Pearson correlation coefficient (R). ****p<0.0001, unpaired Student's *t*‐test.

In light of these findings, we re‐evaluated the affinity pull‐down data and also took into consideration the recently reported biological activity of the natural product salinomycin, which sequesters iron to the lysosomes, inhibiting autophagy and causing ferroptosis.^29^ A similar phenotype has also been reported for the lipophilic iron chelators di‐2‐pyridylketone 4,4‐dimethyl‐3‐thiosemicarbazone (Dp44mT) and, to a lower extent, desferrioxamine (DFO).^30^ We speculated that the ability of autoquin to pull down FECH might have been iron‐dependent and not specific to FECH. This hypothesis was strengthened by reports that quinine and related cinchona alkaloids are able to chelate iron, contributing to their anti‐malarial effect.^31^ To assess whether autoquin displayed a similar mechanism of action to salinomycin, we evaluated its impact on lysosomal mass, lysosomal Fe^2+^, and ROS production. Autoquin significantly increased lysosomal mass after 24 hours, as assessed by Lysotracker deep red (DR) staining (Figure 3 a,b). A decrease in lysotracker staining at early time points (<4 hours) owing to increased pH is generally observed for lysosomotropic compounds including autoquin (Figure 2 b,c); however, this effect is reversed at later time points (>24 hours) as cells adapt to protect themselves from lysosomal stress.^32^ Autoquin also significantly enhanced lysosomal Fe^2+^ levels (Figure 3 a,c), as assessed by the turn‐on fluorescent probe RhoNox‐M, and overall Fe^2+^ levels, as assessed by RhoNox‐1 fluorescence (Figure 3 d). The concomitant increase in lysosomal mass and Fe^2+^ levels also resulted in a highly significant increase of lysosomal reactive oxygen species (ROS), as assessed by CellRox DR (Figure 3 e and Figure S4 a,b). Similarly to salinomycin, autoquin also induced lipid peroxidation, as assessed by a BODIPY 581/591 undecanoic acid (C11) probe^33^ (Figure S4 d) and caused lipid membrane permeabilization (Figure S4 e). As salinomycin had shown promising results in the selective targeting of breast cancer stem cells,^29^, ^34^ a tumorigenic cell subpopulation typically associated with resistance to chemotherapy and sustained tumor growth, it was speculated that autoquin may display a similar profile and potential. Autoquin was selectively cytotoxic against transformed human mammary epithelial HMLER CD44^high^/CD24^low^ cells (HMLER CD24^low^), an established model of human breast cancer stem cells, compared to a control isogenic cell line (HMLER CD24^high^) (Figure S4 f). As such, autoquin represents a new lysosomotropic compound that selectively targets breast cancer stem cells, and may hold promise in the study of anti‐cancer agents that target the lysosomes,^35^ a field that has been growing steadily in recent years, with multiple clinical trials focused on the approved anti‐malarials chloroquine and hydroxychloroquine.

## Conclusion

We have employed the cell painting assay to identify the MMOA of autoquin, a cinchona‐alkaloid derived autophagy inhibitor. While affinity‐based proteomic experiments proved inconclusive, image‐based profiling identified similarities in phenotypic profiles determined for autoquin and known lysosomotropic compounds. This MMOA does not require a direct binding event between autoquin and a target protein, which explains why MS‐based proteomic approaches were inconclusive. However, pull‐down experiments suggested that perturbation of iron homeostasis may contribute to the activity of autoquin, which was confirmed using markers for lysosomal ROS and lipid peroxidation. This work showcases image‐based profiling as an excellent complementary tool for mode‐of‐action and target identification. Given that the lack of identification strategies for non‐protein targets has been a major drawback of MS‐based approaches, we envisage and indeed encourage the chemical biology community to embrace the cell painting assay as an additional, alternative technique for the discovery of modes of action and novel bioactivity. Crucially, this technique obviates the need for functionalisation of the active molecule, making it particularly suitable for the identifying the MMOA of complex, NP‐like compounds. To further improve cell painting and increase its adoption, significantly larger libraries of reference compounds will be required. Although truly novel modes of action will still require proteomic experiments including pull‐downs and/or thermal proteome profiling to identify the molecular targets underlying an observed phenotype, the cell painting assay is a strong addition to the chemical biologist's tool‐kit for tackling the challenge of target identification.

## Conflict of interest

The authors declare no conflict of interest.

## Supporting information

As a service to our authors and readers, this journal provides supporting information supplied by the authors. Such materials are peer reviewed and may be re‐organized for online delivery, but are not copy‐edited or typeset. Technical support issues arising from supporting information (other than missing files) should be addressed to the authors.

Supplementary
