# Supplementary material for: Image‐Based Morphological Profiling Identifies a Lysosomotropic, Iron‐Sequestering Autophagy Inhibitor
Source: Angew Chem Int Ed Engl. 2020 Jan 24;59(14):5721–9. doi: 10.1002/anie.201913712 (PMC7154763; doi:10.1002/anie.201913712)
Supplement: Supplementary file 1 — Supplementary [file ANIE-59-5721-s001.pdf]

Angewandte Chemie
Eine Zeitschrift der Gesellschaft Deutscher Chemiker

Supporting Information

Image-Based Morphological Profiling Identifies a Lysosomotropic, Iron-Sequestering Autophagy Inhibitor

*Luca Laraia, Guillaume Garivet, Daniel J. Foley, Nadine Kaiser, Sebastian Müller, Sarah Zinken, Thomas Pinkert, Julian Wilke, Dale Corkery, Axel Pahl, Sonja Sievers, Petra Janning, Christoph Arenz, Yaowen Wu, Raphaël Rodriguez, and Herbert Waldmann**

anie_201913712_sm_miscellaneous_information.pdf

Supplementary Figures and Tables

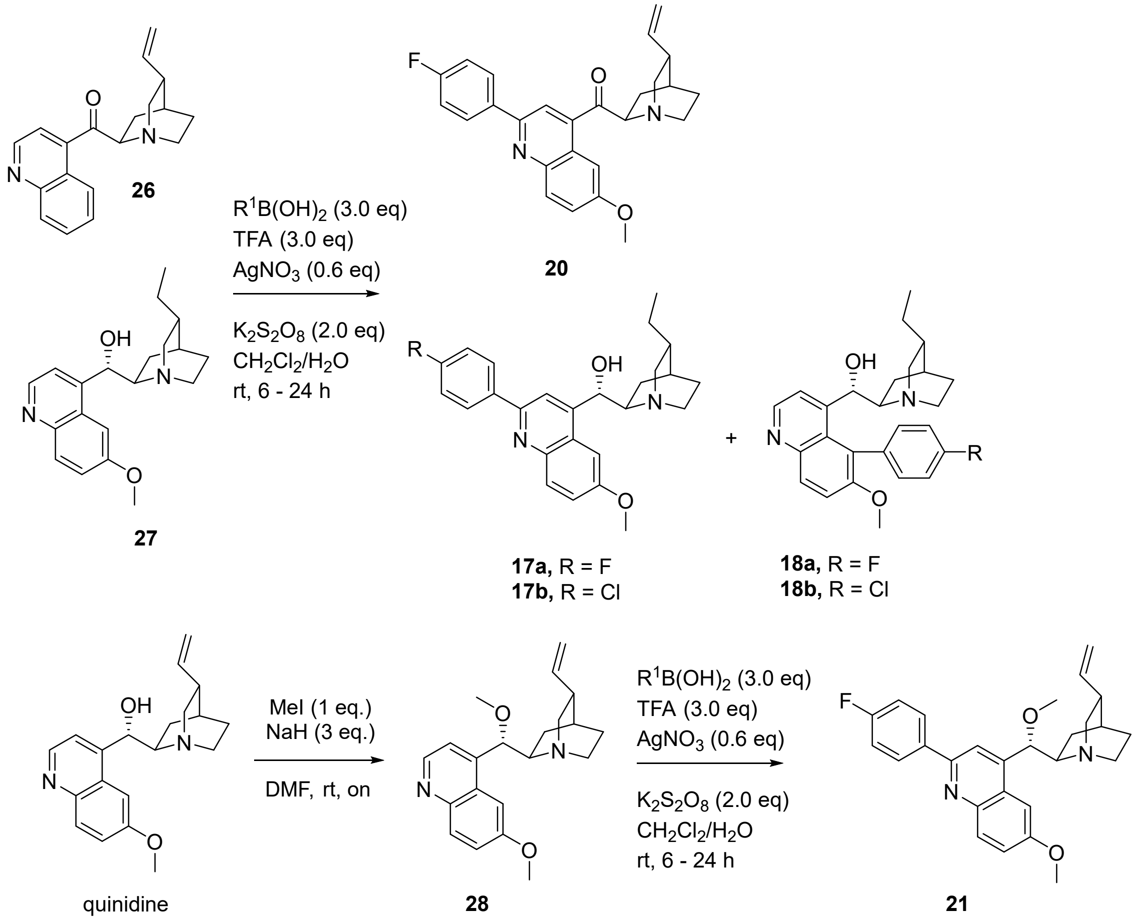

Supplementary Scheme 1. Synthesis of additional autoquin derivatives.

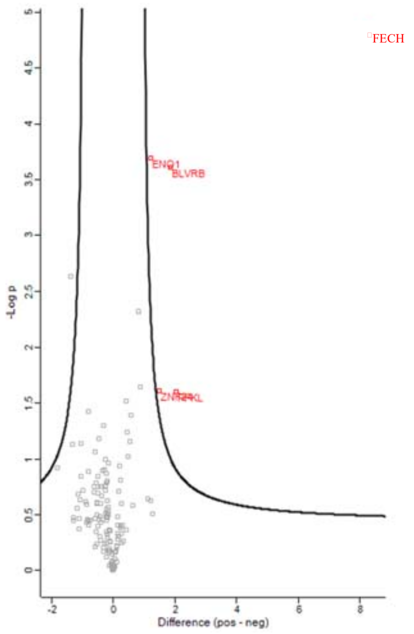

Supplementary Figure 1. Volcano plot of mass spectrometry analysis of pull-down data. Each data point is the mean of a biological replicate conducted in triplicate.

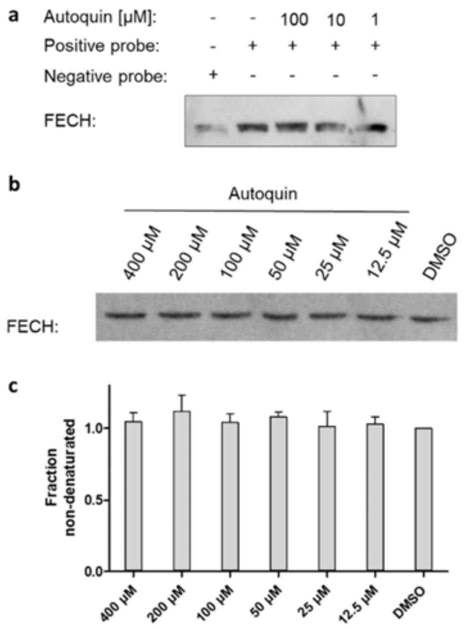

Supplementary Figure 2. Devalidation of Ferrochelatase (FECH) as a direct target of autoquin. (a) Competitive pull-down results using analysed by Western blot using a FECH antibody. N = 3, representative blot shown. (b) Isothermal dose response fingerprint (ITDRF) of Ferrochelatase at increasing autoquin concentrations. N = 2, representative blot shown. (c) quantification of blots shown in (b). Data is mean of 2 independent experiments.

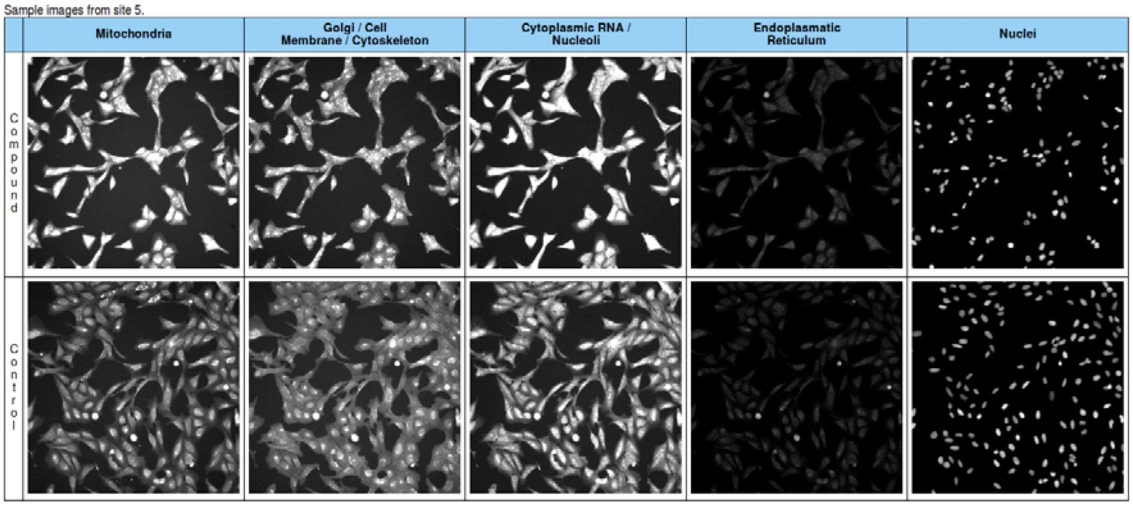

Supplementary Figure 3. Representative images from the cell painting assay (see main text Figure 2a).

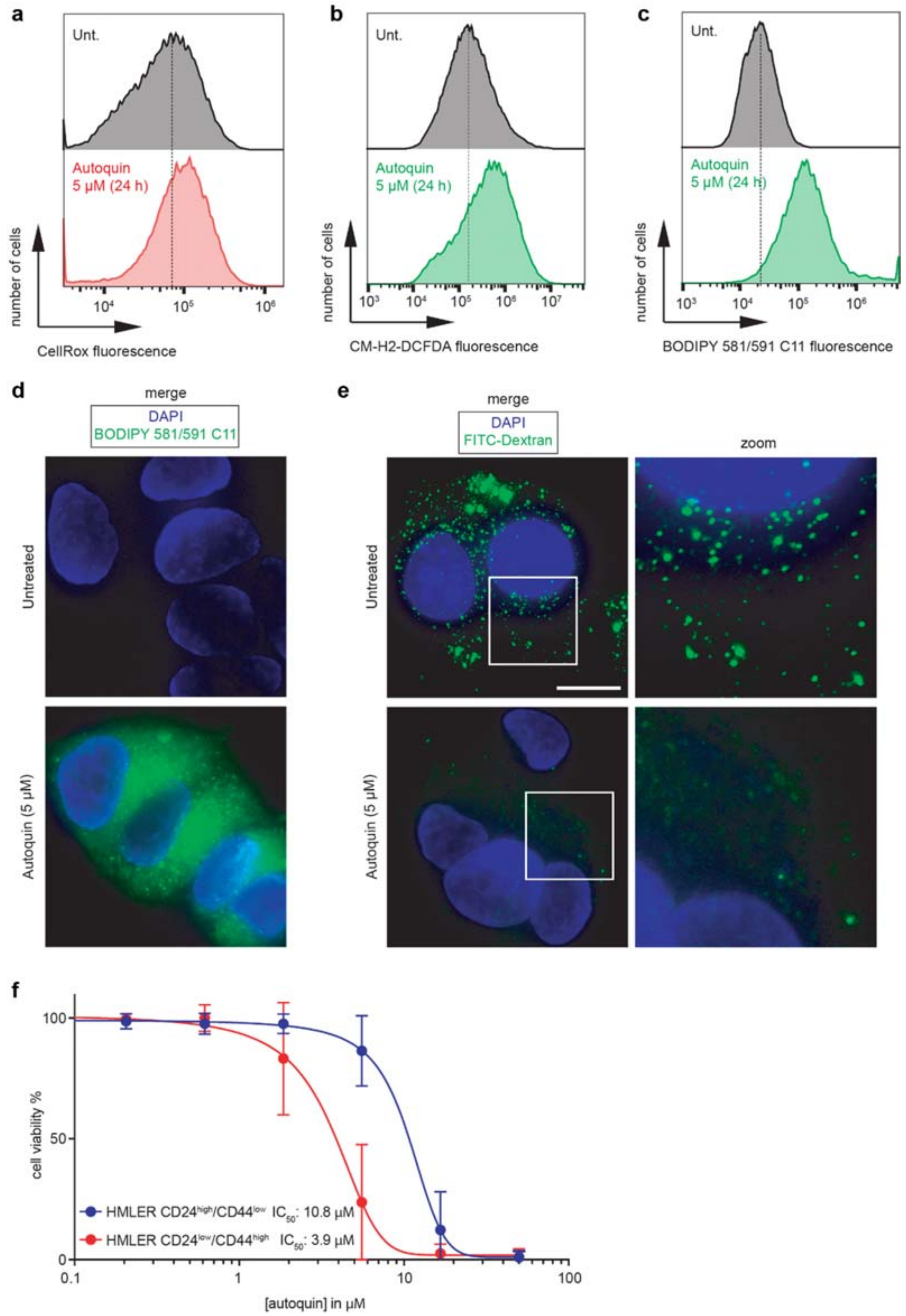

Supplementary Figure 4. Autoquin induces cellular ROS production and lipid peroxidation, and is selectively toxic towards cancer stem cells. (a) Flow cytometry of cells treated as shown in Figure 3e. (b) Total cellular ROS as assessed by flow cytometry of MCF7 cells treated with vehicle of autoquin (5 µM) for 24 h and stained with CM-H2-DCFDA. (c) Flow cytometry

measurement of lipid ROS by means of fluorogenic reaction with BODIPY 581/591 C11 in cells treated with autoquin (0.5 μM) at 48 h. (d) Fluorescence microscopy images showing lipid ROS (green), as described in (c); scale bar, 10 μm. (e) Fluorescence microscopy images of cells treated with Autoquin and analyzed using FITC-Dextran. (f) Cell viability curves of HMLER cell isoforms. n = 3 biological replicates.

Supplementary Table 1. Comparison of calculated physicochemical properties for autoquin and the most similar compounds identified in the cell painting assay.

Name	Structure	pKb	logD [pH =4.0]	logD [pH =5.0]	logD [pH =7.4]	logP	Polar surface area
perphenazine	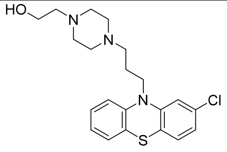	7.81	0.36	0.96	3.14	3.7	29.95
loperamide	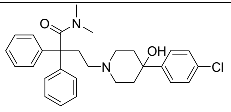	9.41	1.28	1.32	2.77	4.8	43.78
toremifene	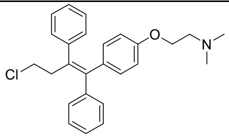	8.76	2.79	2.96	4.89	6.3	12.47
autoquin	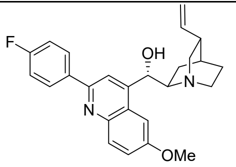	9.24	1.08	1.25	2.85	4.7	45.59

General directions

Unless otherwise noted, chemicals were obtained from Sigma Aldrich, Acros, TCI, or Alfa and were used without further purification. Dry solvents (DCM, THF and MeOH) were purchased and used without further purification unless stated otherwise. All other solvents or reagents were purified according to standard procedures or were used as received from Sigma Aldrich, Alfa Aesar, Acros, Fisher Scientific, Merck and TCI. Milli-Q grade water was used for all experiments. All reactions involving air or moisture sensitive reagents or intermediates were carried out following standard Schlenk line technique under an argon atmosphere and all the glassware were dried by heat gun under high vacuum prior to use. TLC was performed using pre-coated TLC Silica gel 60 F254 aluminum plates (Merck, Darmstadt), detection of compounds was performed by UV254 light and/or dipping into a solution of $KMnO_4$ (1.5 g in 400 mL H_2O, 5.0 g $NaHCO_3$) followed by heating with a heat gun. Column chromatography was performed using silica gel from Acros Organics (40–65 µm, 230–400 mesh). Flash master chromatography was performed using a Reveleris® X2 Flash System (Büchi) and GraceResolveTM cartridges.

^{1}H and ^{13}C NMR spectroscopic data were recorded on a Varian Mercury VX 400, Bruker Avance DRX 400 or 500, Varian Unity Inova 600 at rt unless stated otherwise. NMR spectra were recorded using CD_3OD, $CDCl_3$, CD_2Cl_2 or DMSO-d_6 or as solvent. Chemical shift (δ) values are reported in ppm with the solvent resonance as internal standard (CD_3OD: δ = 3.31 ppm for ^{1}H, δ = 49.00 ppm for ^{13}C), ($CDCl_3$: δ = 7.26 ppm for ^{1}H, δ = 77.16 ppm for ^{13}C), (CD_2Cl_2: δ = 5.32 ppm for ^{1}H, δ = 53.84 for ^{13}C), (DMSO-d_6: δ = 2.50 ppm for ^{1}H, δ = 39.52 ppm for ^{13}C), multiplicities are indicated b.s (broadened singlet), s (singlet), d (doublet), t (triplet), q (quartet), m (multiplet), dd (doublet of doublet), dt (doublet of triplet), ddd (doublet of doublet of doublet), coupling constants (*J*) are given in Hertz (Hz). Unless stated otherwise all NMRs were measured at room temperature. Where possible, structural assignments were performed using standard 2-D NMR techniques (gCOSY, gHSQC, gHMBC) and using 3-D NMR techniques (NOESY).

High Pressure Liquid Chromatography (HPLC) was carried out on an Agilent HPLC (1100series) using a reversed phase column (C4 or C18, 5 µm, 250 x 4.6 mm) equipped with a Finnigan LCQ ESI spectrometer and an UV/VIS detector operating at 210 and 280 nm (flow rate: 1.0 mL/min, time: 15 min, solvents A: 0.1% HCOOH in water, B: 0.1% HCOOH in acetonitrile, 1 min 10% B, in 10 min to 100% B). Preparative HPLC runs were performed on an Agilent HPLC (1100 series) using a reversed-phase C4 or C18 column (RP C4 or C18, solvents A: 0.1% TFA in water, B: 0.1% TFA in acetonitrile flow rate: 20.0 mL/min, for 5% B to 100% B over 55 min).

HRMS-ESI were taken on an Accela HPLC-System (HPLC column 50/1 Hypersil GOLD1.9 µm) with an LTQ Orbitrap mass spectrometer from Thermo Scientific. (ESI)-MS were measured by using an Agilent 1100 series binary pump together with a reversed-phase HPLC column (Macherey-Nagel), ionization method: electron spray ionization. Optical rotations were measured using a Schmidt & Hänsch Polartronic polarimeter in cuvettes with a path length of 10 cm. Concentration and solvent used for the measurement are specified for each value.

General Procedure I: Borono-Minisci Reaction

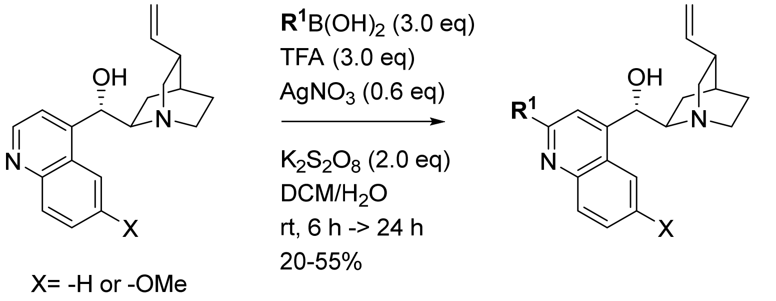

To a solution of the selected alkaloid (40 mg, 120 µmol) in DCM (1.25 mL) was added trifluoroacetic acid (28 µL, 370 µmol) followed by arylboronic acid (3.0 eq). Water (0.75 mL) was then added, followed by silver (I) nitrate (8 mg, 40 µmol) in water (0.50 mL). Potassium persulfate (100 mg, 370 µmol) was then added and the solution was stirred vigorously at rt. After 16 h the reaction was worked up by quenching with 3 mL of sat. $NaHCO_3$ and diluting with 5 mL CH_2Cl_2. The organic layer was separated and the aqueous was washed with 3 x 5 mL *i*PrOH:$CHCl_3$ (10%) mixture. The organic layers were combined, dried (Na_2SO_4) and filtered. The solvent was removed in vacuo and the mixture was purified by FC eluting 1–4 % (MeOH:$CHCl_3$) or by mass-directed preparative HPLC.

General Procedure II: Suzuki coupling of aryl chloride

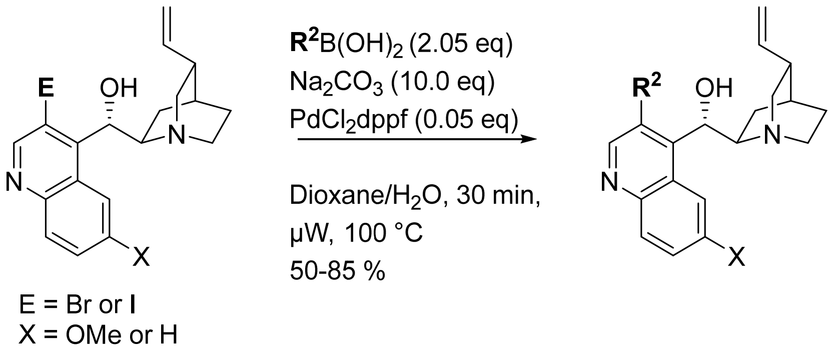

To a microwave tube charged with a solution of aryl bromide (25 mg, 65 µmol, 1.0 eq) and boronic acid (100 µmol, 1.5 eq) in dioxane (2 mL) was added a solution of Na_2CO_3 (69 mg, 0.65 mmol, 10 eq) in H_2O (1 mL) at rt. The reaction was degassed for 10 min with argon. Then, $PdCl_2dppf$ (13 mg, 15 µmol, 5 mol%) was added. The reaction mixture was stirred for 1 h at 100 °C in a CEM Discover microwave reactor. The reaction was cooled to room temperature, filtered through a pad of Celite® and the Celite washed with EtOAc (3 x 20 mL). The organic phase was washed with brine. The resulting aqueous phase was extracted with 2 x 20 mL 10%

*i*PrOH in $CHCl_3$. Organic phases were dried (Na_2SO_4), filtered, and the solvent removed under reduced pressure. The crude was purified by preparative HPLC (ACN/H_2O + 0.1 % TFA) or a Reveleris® X2 Flash System (DCM/1-5% MeOH).

General Procedure III: Heck reactions

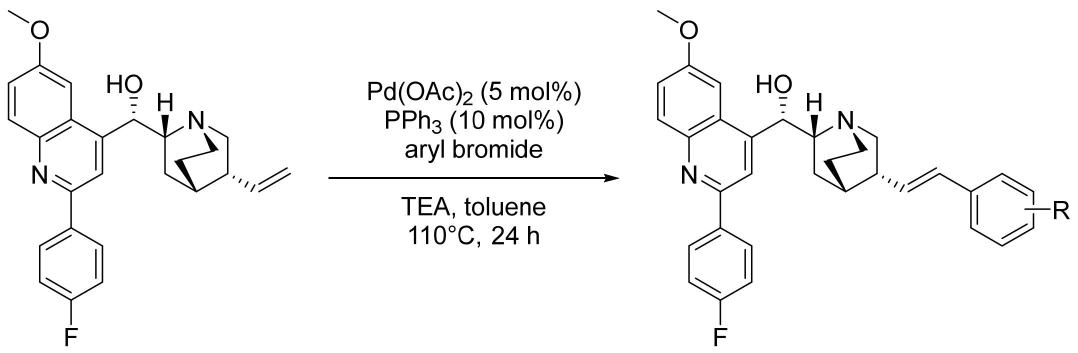

Autoquin (50 mg, 0.12 mmol, 1 eq.) and $Pd(OAc)_2$ (1.34 mg, 0.06 mmol, 0.05 eq.) were placed into a heat- and vacuum-dried schlenk-type flask under Argon and dissolved in anhydrous toluene (0.2 M, 0.5 mL). Then bromobenzene (2 eq.) and TEA (33 µL, 0.24 mmol, 2 eq.) were added dropwise. The mixture then was placed to a preheated heating block and stirred at 110°C for 24 hours under Argon. The reaction then was allowed to cool to room temperature, diluted with 3 mL MeOH and filtered through a syringe filter (pore size: 0.45 µm) two times. Solvents were removed *in vacuo*. Purification by mass directed preparative HPLC using 10-60% acetonitrile + 0.1% TFA in H_2O + 0.1% TFA or Flash Column Chromatography eluting with 0-10% MeOH in DCM gave the desired product.

C-2/C-7 substituted and side products compound collection

(S)-(2-(4-fluorophenyl)-quinolin-4-yl)((2R,4S,5R)-5-vinylquinuclidin-2-yl)methanol-trifluoroacetate (8a)

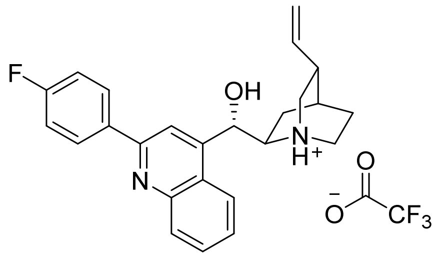

The title compound was synthesized following **General Procedure I** starting with cinchonine (40.0 mg, 136.0 µmol, 1.0 eq) and 4-Fluorophenylboronic acid (57.0 mg, 405 µmol, 3.0 eq). Purified using preparative HPLC (ACN/H_2O + 0.1 % TFA) to afford the product as an amorphous solid (23 mg, 38%).

^{1}H NMR (600 MHz, MeOD): δ = 8.31 (s, 1H), 8.29 (d, J = 8.4 Hz, 1H), 8.25 – 8.18 (m, 3H), 7.94 – 7.89 (m, 1H), 7.79 – 7.74 (m, 1H), 7.37 – 7.31 (m, 2H), 6.13 (s, 1H), 5.76 (ddd, J = 17.3, 10.5, 7.0 Hz, 1H), 5.12 (d, J = 17.2 Hz, 1H), 5.02 (d, J = 10.5 Hz, 1H), 4.31 – 4.25 (m, 1H), 3.75 (t, J = 8.9 Hz, 1H), 3.63 (dd, J = 13.0, 10.7 Hz, 1H), 3.38 – 3.31 (m, 2H), 2.82 (dd, J = 3.4, 1.7 Hz, 1H), 2.32 – 2.27 (m, 1H), 2.26 – 2.20 (m, 1H), 2.12 (dd, J = 5.9, 2.9 Hz, 1H), 2.01 – 1.94 (m, 1H), 1.62 (m, 1H); **^{13}C NMR** (151 MHz, MeOD): δ = 165.95 (d, J = 250.1 Hz), 162.29 (q, J = 36.1 Hz), 156.97, 151.69, 146.94, 139.15, 134.75, 132.58, 131.53 (d, J = 8.8 Hz), 129.31, 128.85, 125.28, 124.05, 118.58, 117.15 (d, J = 22.3 Hz), 68.74, 61.75, 55.58, 49.57, 45.49, 38.50, 28.21, 25.15, 19.43; **^{19}F NMR** (565 MHz, MeOD): δ = 77.03, -112.10; **HRMS (ESI+)**: *m/z* = 389.2023 ($[M + H]^+$, calcd. for $C_{25}H_{26}ON_2F^+$: 389.2024) (Δ = 0.30 ppm)– No TFA visible; $[\boldsymbol{\alpha}]_{20}^{D}$ = + 71° (c = 1.0, MeOH).

(*R*)-(2-(4-fluorophenyl)-quinolin-4-yl)((2*S*,4*S*,5*R*)-5-vinylquinuclidin-2-yl)methanol-trifluoroacetate (12a)

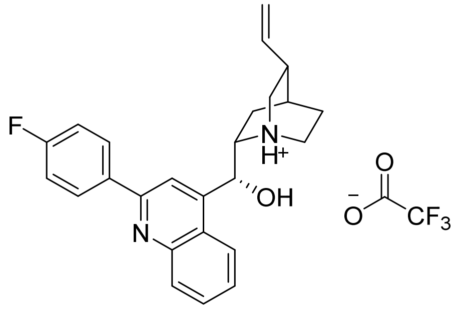

The title compound was synthesized following **General Procedure I** starting with cinchonidine (40.0 mg, 136.0 µmol, 1.0 eq) and 4-Fluorophenylboronic acid (57.0 mg, 465 µmol, 3.0 eq). Purified using preparative HPLC (ACN/H_2O + 0.1 % TFA) to afford the product as an amorphous solid (19 mg, 32%).

^{1}H NMR (500 MHz, MeOD): δ = 8.27 (s, J = 9.3 Hz, 1H), 8.24 – 8.18 (m, 4H), 7.87 (t, J = 7.7 Hz, 1H), 7.72 (t, J = 7.7 Hz, 1H), 7.31 (t, J = 8.7 Hz, 2H), 6.06 (s, 1H), 5.75 (ddd, J = 17.3, 10.4, 7.1 Hz, 1H), 5.11 (d, J = 17.1 Hz, 1H), 5.02 (d, J = 10.5 Hz, 1H), 4.31 – 4.23 (m, 1H), 3.74 (t, J = 8.8 Hz, 1H), 3.62 (dd, J = 12.8, 10.8 Hz, 1H), 3.38 – 3.31 (m, 1H), 2.82 (s, 1H), 2.32 – 2.19 (m, 2H), 2.12 (t, J = 4.9 Hz, 1H), 1.96 (t, J = 9.7 Hz, 1H), 1.61 (dd, J = 13.3, 10.6 Hz, 1H), 1.36 – 1.17 (m, 1H); **^{13}C NMR** (126 MHz, MeOD): δ = 165.75 (d, J = 249.5 Hz), 162.38, 157.27, 150.32, 148.08, 139.14, 135.68, 132.03, 131.26 (d, J = 8.7 Hz), 129.88, 128.98, 125.18, 123.73, 118.25, 117.27, 117.02 (d, J = 22.1 Hz), 68.77, 61.79, 55.58, 49.63, 45.56, 38.53, 28.19, 25.16, 19.47; **^{19}F NMR** (565 MHz, MeOD): δ = -77.06, -111.82; **HRMS (ESI+)**: *m/z* = 389.2025 ([*M* + H]$^+$, calcd. for $C_{25}H_{26}ON_2F^+$: 389.2024) (Δ = 0.26 ppm)– No TFA visible; $[\boldsymbol{\alpha}]_{\mathbf{20}}^{\boldsymbol{D}}$ = - 34° (c = 1.0, MeOH).

(*R*)-(7-(4-fluorophenyl)-quinolin-4-yl)((2*S*,4*S*,5*R*)-5-vinylquinuclidin-2-yl)methanol-trifluoroacetate (13a)

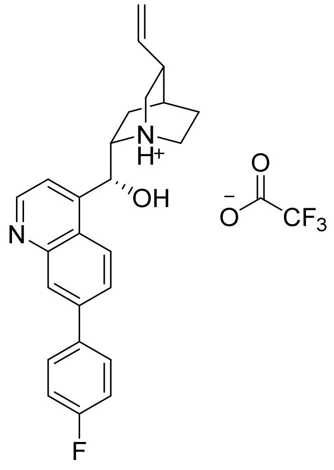

The compound was synthesized following **General Procedure I**. Crude was purified using preparative HPLC (ACN/H_2O + 0.1 % TFA) to afford the pure compound (2 mg, 3%).

HRMS (ESI+): *m/z* = 389.2024 ($[M + H]^+$, calcd. for $C_{25}H_{26}ON_2F^+$: 389.2024) – No TFA visible., $[\alpha]_{20}^{D}$= -38° (c = 1.0, MeOH). (Δ = 0.01 ppm)

^{1}H NMR (500 MHz, MeOD): δ = 8.87 (d, J = 20.6 Hz, 1H), 8.31 – 8.19 (m, 1H), 8.16 – 8.04 (m, 1H), 7.98 (d, J = 7.4 Hz, 1H), 7.85 (dd, J = 12.5, 4.7 Hz, 1H), 7.76 (dd, J = 8.5, 5.3 Hz, 2H), 7.20 (dd, J = 11.5, 5.6 Hz, 2H), 5.98 (d, J = 25.2 Hz, 1H), 5.77 – 5.62 (m, 1H), 5.11 – 5.00 (m, 2H), 4.97 – 4.89 (m, 1H), 4.25 – 4.04 (m, 1H), 3.72 – 3.61 (m, 1H), 3.55 (dd, J = 23.6, 11.2 Hz, 1H), 2.74 (s, J = 6.9 Hz, 1H), 2.30 – 2.10 (m, 2H), 2.06 – 2.00 (m, 1H), 1.96 – 1.84 (m, 1H), 1.62 – 1.44 (m, 1H), 1.35 – 1.15 (m, 1H).

(S)-(2-(4-fluorophenyl)-6-methoxyquinolin-4-yl)((2R,4S,5R)-5-vinylquinuclidin-2-yl)methanol- trifluoroacetate (2, autoquin) ***and*** **(S)-(5-(4-fluorophenyl)-6-methoxyquinolin-4-yl)((2R,4S,5R)-5-vinylquinuclidin-2-yl)methanol- trifluoroacetate (9a)**

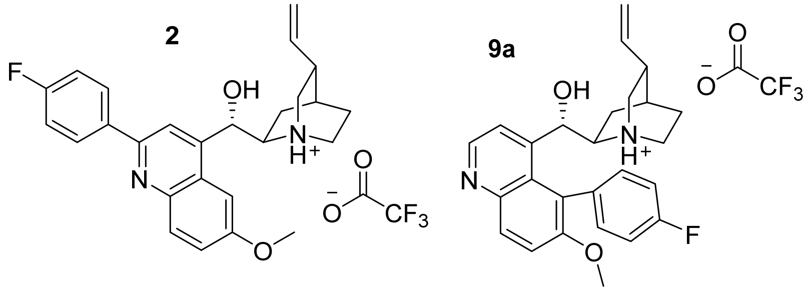

The title compounds were synthesized following **General Procedure I** starting with quinidine (40.0 mg, 120.0 µmol, 1.0 eq) and 4-Fluorophenylboronic acid (50.0 mg, 360 µmol, 3.0 eq). Purified using preparative HPLC (ACN/H_2O + 0.1 % TFA) to afford the **3a** solid (19 mg, 32%) and xx (5 mg, 8%) as an amorphous solids.

3a: ^{1}**H NMR** (700 MHz, MeOD): δ = 8.16 (s, 1H), 8.07 – 8.03 (m, 3H), 7.50 (dd, J = 9.2, 1.9 Hz, 1H), 7.40 (d, J = 1.6 Hz, 1H), 7.25 (t, J = 8.6 Hz, 2H), 6.12 (s, 1H), 6.07 – 6.01 (m, 1H), 5.22 – 5.15 (m, 2H), 4.19 (ddd, J = 17.3, 10.4, 7.1 Hz, 1H), 3.95 (s, 3H), 3.64 (app t, J = 9.4 Hz, 1H), 3.45 (dd, J = 15.8, 7.3 Hz, 2H), 3.27 (dd, J = 21.0, 10.1 Hz, 1H), 2.67 (dd, J = 17.4, 8.5 Hz, 1H), 2.48 – 2.43 (m, 1H), 1.95 (s, 1H), 1.88 (t, J = 11.3 Hz, 1H), 1.79 (dd, J = 21.2, 10.8 Hz, 1H), 1.25 – 1.19 (m, 1H); ^{13}**C NMR** (176 MHz, MeOD): δ = 164.13 (d, J = 249.3 Hz), 161.24 (q, J = 36.2 Hz), 159.44, 152.60, 149.33, 140.66, 136.75, 132.85, 129.95 (d, J = 8.7 Hz), 128.19, 125.30, 124.10, 118.77, 117.69, 117.19 (d, J=22.2 Hz), 100.98, 67.66, 59.82, 55.45, 49.09, 48.47, 36.97, 27.32, 22.63, 17.58; ^{19}**F NMR** (565 MHz, MeOD): δ = -77.10, -111.59; **HRMS (ESI+)**: *m/z* = 419.2127 ([*M* + H]$^+$, calcd. for $C_{26}H_{28}O_2N_2F^+$: 419.2129) (Δ = 0.66 ppm)– No TFA visible; $[\alpha]_{20}^{D}$ = + 62° (c = 1.0, MeOH).

(5a): ^{1}**H NMR** (600 MHz, MeOD): δ = 8.98 (d, J = 5.4 Hz, 1H), 8.36 (dd, J = 10.6, 7.4 Hz, 2H), 8.21 (dd, J = 27.1, 7.4 Hz, 1H), 8.05 (d, J = 9.4 Hz, 1H), 7.39 (td, J = 8.6, 2.7 Hz, 1H), 7.33 – 7.25 (m, 1H), 7.21 – 7.15 (m, 1H), 5.86 (ddd, J = 17.3, 10.5, 6.8 Hz, 1H), 5.24 – 5.14 (m, 2H), 4.08 (s, J = 9.7 Hz, 1H), 3.96 – 3.91 (m, 1H), 3.89 (s, 3H), 3.25 – 3.13 (m, 2H), 2.64 – 2.56 (m, 1H), 2.33 – 2.29 (m, 1H), 2.04 – 1.97 (m, 1H), 1.91 – 1.75 (m, 3H), 1.33 – 1.24 (m, 1H), 0.71 – 0.62 (m, 1H). ^{19}**F NMR** (565 MHz, MeOD): δ = -76.50, -112.01. **HRMS (ESI+)**:

m/z = 419.2127 ([*M* + H]$^+$, calcd. for $C_{26}H_{28}O_2N_2F^+$: 419.2129) – No TFA visible., $[\alpha]_{20}^{D}$= +50° (c = 1.0, MeOH). (Δ = 0.50 ppm)

(*R*)-(2-(4-fluorophenyl)-6-methoxyquinolin-4-yl)((2*S*,4*S*,5*R*)-5-vinylquinuclidin-2-yl)methanol- trifluoroacetate (11a)

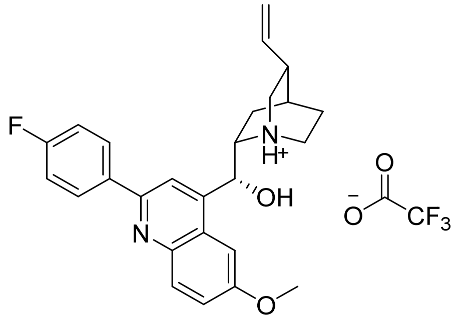

The title compound was synthesized following **General Procedure I** starting with quinidine (40.0 mg, 120.0 µmol, 1.0 eq) and 4-Fluorophenylboronic acid (50.0 mg, 360 µmol, 3.0 eq). Purified using preparative HPLC (ACN/H_2O + 0.1 % TFA) to afford the product as an amorphous solid (25 mg, 39%).

^{1}H NMR (700 MHz, MeOD): δ = 8.30 (s, J = 13.9 Hz, 1H), 8.21 – 8.11 (m, 3H), 7.60 (d, J = 9.2 Hz, 1H), 7.52 (s, 1H), 7.35 (t, J = 8.5 Hz, 2H), 6.15 (s, J = 23.4 Hz, 1H), 5.79 (ddd, J = 17.3, 10.4, 7.1 Hz, 1H), 5.14 (d, J = 17.1 Hz, 1H), 5.05 (d, J = 10.5 Hz, 1H), 4.29 (dd, J = 15.7, 7.2 Hz, 1H), 4.05 (s, J = 12.2 Hz, 3H), 3.77 – 3.70 (m, 1H), 3.70 – 3.63 (m, 1H), 3.40 – 3.32 (m, 2H), 2.84 (s, 1H), 2.31 (dd, J = 12.8, 7.6 Hz, 1H), 2.29 – 2.20 (m, 1H), 2.13 (d, J = 2.3 Hz, 1H), 1.99 (t, J = 10.2 Hz, 1H), 1.69 – 1.63 (m, 1H); **^{13}C NMR** (176 MHz, MeOD): δ = 165.70 (d, J = 250.12 Hz), 161.86 (d, J = 27.74 Hz), 160.80, 153.95, 151.03, 141.90, 139.24, 134.15, 131.27, 131.22, 129.51, 126.73, 125.50, 119.09, 117.23, 117.19 (d, J = 22.1 Hz), 102.46, 68.66, 61.31, 56.95, 55.58, 49.53, 45.36, 38.54, 28.30, 25.17, 19.43; **^{19}F NMR** (377 MHz, MeOD): δ = -76.85, -112.23; **HRMS (ESI+)**: *m/z* = 419.2124 ([*M* + H]$^+$, calcd. for $C_{26}H_{28}O_2N_2F^+$: 419.2129) (Δ = 1.12 ppm)– No TFA visible; $[\boldsymbol{\alpha}]_{20}^{D}$ = - 40° (c = 1.0, MeOH).

(S)-(2-(4-chlorophenyl)-quinolin-4-yl)((2R,4S,5R)-5-vinylquinuclidin-2-yl)methanol-trifluoroacetate (8b) *and* (S)-(7-(4-chlorophenyl)-quinolin-4-yl)((2R,4S,5R)-5-vinylquinuclidin-2-yl)methanol- trifluoroacetate (10b)

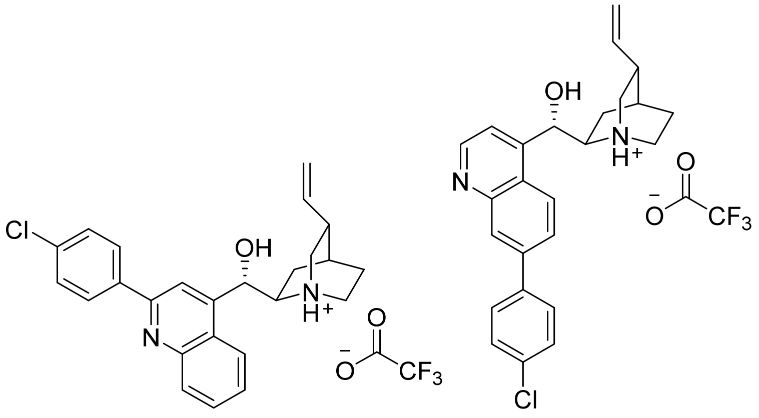

The title compounds were synthesized following **General Procedure I** starting with cinchonine (40.0 mg, 135.0 µmol, 1.0 eq) and 4-Chlorophenylboronic acid (63.0 mg, 405 µmol, 3.0 eq). Purified using preparative HPLC (ACN/H_2O + 0.1 % TFA) to afford **4b** (30 mg, 48%) and **6b** (5 mg, 8%) as an amorphous solids.

4b: ^{1}H NMR (600 MHz, MeOD): δ = 8.23 (s, 1H), 8.19 – 8.14 (m, 4H), 7.83 (dd, J = 11.8, 4.5 Hz, 1H), 7.73 – 7.69 (m, 1H), 7.57 (d, J = 8.6 Hz, 2H), 6.14 (s, 1H), 6.13 – 6.08 (m, 1H), 5.25 (dd, J = 13.8, 9.2 Hz, 2H), 4.27 (ddd, J = 12.4, 8.4, 2.4 Hz, 1H), 3.73 (t, J = 9.5 Hz, 1H), 3.59 – 3.49 (m, 2H), 3.34 (ddd, J = 12.2, 10.1, 8.5 Hz, 1H), 2.74 (q, J = 8.6 Hz, 1H), 2.53 – 2.47 (m, 1H), 2.02 (s, 1H), 1.98 – 1.91 (m, 1H), 1.85 (ddd, J = 12.7, 10.2, 1.3 Hz, 1H), 1.31 – 1.25 (m, 1H); **^{13}C NMR** (151 MHz, MeOD): δ = 162.35 (d, J = 36.1 Hz), 157.17, 149.16, 149.00, 138.65, 138.07, 137.30, 131.59, 130.75, 130.32, 130.22, 128.83, 125.28, 123.60, 118.80, 117.71 (d, J = 9.4 Hz), 116.87, 69.05, 61.64, 50.55, 50.09, 49.85, 49.57, 38.38, 28.66, 23.97, 19.09; **HRMS (ESI+)**: *m/z* = 407.1696 ([*M* + H]$^+$, calcd. for $C_{25}H_{26}ON_2$ $^{37}Cl^+$: 407.1699) (Δ = 0.75 ppm) – No TFA visible; $[\boldsymbol{\alpha}]_{\mathbf{20}}^{\boldsymbol{D}}$= + 26° (c = 1.0, MeOH).

6b: ^{1}H NMR (600 MHz, MeOD): δ = 9.01 (dt, J = 25.4, 6.2 Hz, 1H), 8.45 – 8.33 (m, 2H), 8.22 (d, J = 11.2 Hz, 1H), 8.05 – 7.98 (m, 1H), 7.89 – 7.80 (m, 2H), 7.60 – 7.50 (m, 2H), 6.31 – 6.19 (m, 1H), 6.13 – 6.06 (m, 1H), 5.31 – 5.22 (m, 2H), 3.79 – 3.68 (m, 1H), 3.62 – 3.47 (m, 2H), 2.74 (t, J = 13.4 Hz, 1H), 2.56 – 2.38 (m, 1H), 2.01 (dd, J = 16.0, 8.3 Hz, 2H), 1.97 – 1.90 (m, 2H), 1.89 – 1.79 (m, 2H); **HRMS (ESI+)**: *m/z* = 407.1702 ([*M* + H]$^+$, calcd. for $C_{25}H_{26}ON_2$ $^{37}Cl^+$: 407.1699) (Δ = 0.73 ppm) – No TFA visible; $[\boldsymbol{\alpha}]_{\mathbf{20}}^{\boldsymbol{D}}$= +69° (c = 1.0, MeOH).

(S)-(2-(4-chlorophenyl)-6-methoxyquinolin-4-yl)((2R,4S,5R)-5-vinylquinuclidin-2-yl)methanol- trifluoroacetate (7a)

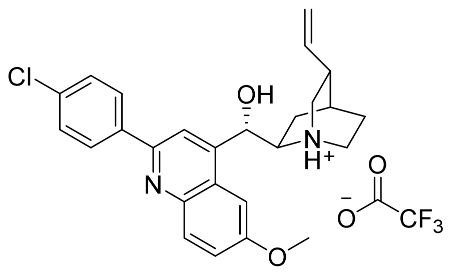

The title compound was synthesized following **General Procedure I** starting with quinidine (40.0 mg, 120.0 µmol, 1.0 eq) and 4-Chlorophenylboronic acid (56.0 mg, 360 µmol, 3.0 eq). Purified using preparative HPLC (ACN/H_2O + 0.1 % TFA) to afford the product **3b** as an amorphous solid (22 mg, 33%).

^{1}H NMR (700 MHz, MeOD): δ = 8.22 (s, 1H), 8.16 – 8.12 (m, 3H), 7.59 (d, J = 8.6 Hz, 2H), 7.54 (dd, J = 9.2, 2.6 Hz, 1H), 7.40 (d, J = 2.6 Hz, 1H), 6.16 (ddd, J = 17.5, 10.4, 7.3 Hz, 1H), 6.09 (d, J = 18.2 Hz, 1H), 5.32 – 5.27 (m, 2H), 4.30 (ddd, J = 12.4, 8.4, 2.4 Hz, 1H), 4.05 (s, 3H), 3.76 (app t, J = 9.4 Hz, 1H), 3.58 (dd, J = 16.5, 9.1 Hz, 2H), 3.25 – 3.20 (m, 1H), 2.79 (dd, J = 17.6, 8.6 Hz, 1H), 2.59 – 2.53 (m, 1H), 2.07 (s, J = 13.9 Hz, 1H), 2.03 – 1.97 (m, 1H), 1.92 (m, 1H), 1.38 – 1.29 (m, 1H); **^{13}C NMR** (176 MHz, MeOD): δ = 162.40 (d, J = 34.7 Hz), 160.36, 154.56, 147.86, 144.67, 138.57, 138.09, 136.96, 131.94, 130.13 (d, J = 21.0 Hz), 126.45, 124.04, 117.84 (d, J = 42.9 Hz), 102.05, 69.19, 61.42, 56.58, 50.51, 49.93, 49.53, 47.93, 38.37, 28.67, 24.02, 19.10; **HRMS (ESI+)**: *m/z* = 437.1803 ([*M* + H]$^+$, calcd. for $C_{26}H_{28}O_2N_2$ $^{37}Cl^+$: 437.1804) (Δ = 0.24 ppm) – No TFA visible; $[\alpha]_{20}^{D}$= + 183° (c = 1.0, MeOH).

(*R*)-(2-(4-chlorophenyl)-quinolin-4-yl)((2*S*,4*S*,5*R*)-5-vinylquinuclidin-2-yl)methanol-trifluoroacetate (12b) *and* (*R*)-(7-(4-chlorophenyl)-quinolin-4-yl)((2*S*,4*S*,5*R*)-5-vinylquinuclidin-2-yl)methanol- trifluoroacetate (13b)

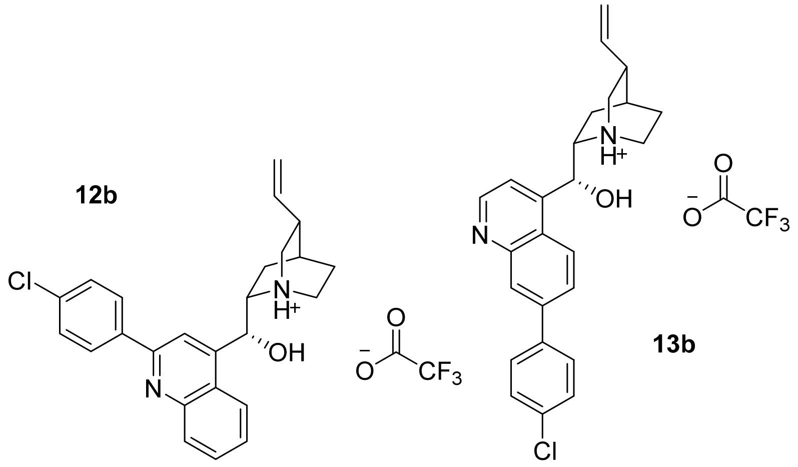

12b 13b

The title compound was synthesized following **General Procedure I** starting with cinchonidine (40.0 mg, 135.0 µmol, 1.0 eq) and 4-Chlorophenylboronic acid (63.0 mg, 405 µmol, 3.0 eq). Purified using preparative HPLC (ACN/H_2O + 0.1 % TFA) to afford the products **12b** (25 mg, 40%) and **13b** (1 mg, 2%) as amorphous solids.

12b: ^{1}**H NMR** (600 MHz, MeOD): δ = 8.27 (s, 1H), 8.20 (dd, J = 13.2, 8.4 Hz, 2H), 8.14 (d, J = 8.6 Hz, 2H), 7.87 (t, J = 7.7 Hz, 1H), 7.73 (t, J = 7.6 Hz, 1H), 7.58 (d, J = 8.6 Hz, 2H), 6.06 (d, J = 0.9 Hz, 1H), 5.74 (ddd, J = 17.3, 10.5, 7.1 Hz, 1H), 5.10 (d, J = 17.2 Hz, 1H), 5.00 (d, J = 10.5 Hz, 1H), 4.29 – 4.23 (m, 1H), 3.72 (t, J = 8.9 Hz, 1H), 3.61 (dd, J = 13.0, 10.7 Hz, 1H), 3.36 – 3.28 (m, 2H), 2.80 (s, J = 3.3 Hz, 1H), 2.30 – 2.19 (m, 2H), 2.10 (dd, J = 5.8, 2.9 Hz, 1H), 1.98 – 1.91 (m, 1H), 1.63 – 1.57 (m, 1H); ^{13}**C NMR** (176 MHz, MeOD): δ = 162.31 (q, J = 35.7 Hz), 157.40, 149.97, 148.92, 139.37, 138.66, 137.68, 132.01, 130.71, 130.65, 130.49, 129.17, 125.52, 123.87, 118.23, 117.50, 69.01, 62.07, 55.85, 49.77, 45.80, 38.76, 28.43, 25.41, 19.74; **HRMS (ESI+)**: *m/z* = 407.1697 ([*M* + H]$^+$, calcd. for $C_{25}H_{26}ON_2$ $^{37}Cl^+$: 407.1699) (Δ = 0.50 ppm) – No TFA visible; $[\boldsymbol{\alpha}]_{\mathbf{20}}^{\boldsymbol{D}}$= -16° (c = 1.0, MeOH).

13b: ^{1}**H NMR** (700 MHz, MeOD): δ = 8.90 (d, J = 4.4 Hz, 1H), 8.20 (dd, J = 8.3, 1.3 Hz, 1H), 7.86 (d, J = 4.4 Hz, 1H), 7.85 – 7.78 (m, 2H), 7.61 – 7.58 (m, 2H), 7.52 – 7.49 (m, 2H), 6.05 (s, 1H), 5.81 (ddd, J = 17.3, 10.5, 7.0 Hz, 1H), 5.17 (d, J = 17.1 Hz, 1H), 5.07 (t, J = 10.1 Hz, 1H), 4.34 – 4.28 (m, 1H), 3.75 (t, J = 8.9 Hz, 1H), 3.67 (dd, J = 13.1, 10.7 Hz, 1H), 3.43 – 3.34 (m, 1H), 2.86 (s, 1H), 2.32 – 2.22 (m, 2H), 2.18 – 2.14 (m, 1H), 2.01 (dt, J = 15.8, 10.5 Hz, 1H), 1.63 (ddd, J = 13.5, 6.3, 3.2 Hz, 1H), 1.31 (d, J = 7.8 Hz, 1H); **HRMS (ESI+)**: *m/z* =

407.1697 ([M + H]$^+$, calcd. for $C_{25}H_{26}ON_2$ $^{37}Cl^+$: 407.1699) (Δ = 0.50 ppm) – No TFA visible; $[\alpha]_{20}^{D}$= -° (c = 1.0, MeOH).

(*R*)-(2-(4-chlorophenyl)-6-methoxyquinolin-4-yl)((2*S*,4*S*,5*R*)-5-vinylquinuclidin-2-yl)methanol- trifluoroacetate (11b)

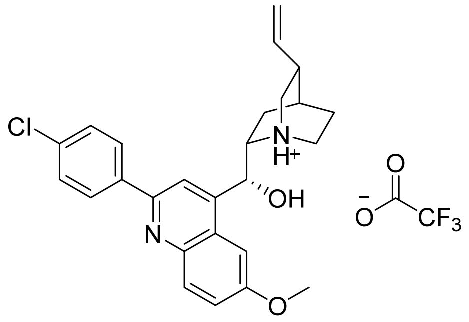

The title compound was synthesized following **General procedure I** starting with quinine (40.0 mg, 120.0 µmol, 1.0 eq) and 4-Chlorophenylboronic acid (56.0 mg, 360 µmol, 3.0 eq). Purified using preparative HPLC (ACN/H_2O + 0.1 % TFA) to afford the product as an amorphous solid (15 mg, 22%).

^{1}H NMR (700 MHz, MeOD): δ = 8.27 (s, 1H), 8.16 – 8.10 (m, 3H), 7.61 – 7.55 (m, 3H), 7.46 (d, J = 2.5 Hz, 1H), 6.07 (s, 1H), 5.80 (ddd, J = 17.4, 10.5, 7.1 Hz, 1H), 5.15 (d, J = 17.1 Hz, 1H), 5.06 (d, J = 10.5 Hz, 1H), 4.34 – 4.25 (m, 1H), 4.05 (s, 3H), 3.79 – 3.71 (m, 1H), 3.66 (dd, J = 13.1, 10.7 Hz, 1H), 3.40 – 3.33 (m, 2H), 2.85 (b.s, 1H), 2.36 – 2.30 (m, 1H), 2.30 – 2.21 (m, 1H), 2.15 (m, 1H), 2.03 – 1.97 (m, 1H), 1.72 – 1.61 (m, 1H); **^{13}C NMR** (176 MHz, MeOD): δ = 162.39 (q, J = 35.7 Hz), 160.53, 154.11, 149.10, 143.48, 139.08, 137.61, 137.22, 130.92, 130.16, 126.50, 124.48, 118.46, 117.15, 116.84, 102.13, 68.62, 61.29, 56.66, 55.47, 49.41, 45.31, 38.43, 28.15, 25.06, 19.38; **HRMS (ESI+)**: *m/z* =437.1805 ([M + H]$^+$, calcd. for $C_{26}H_{28}O_2N_2$ $^{37}Cl^+$: 437.1804) (Δ = 0.16 ppm) – No TFA visible; $[\alpha]_{20}^{D}$= -34° (c = 1.0, MeOH).

(S)-(2-(4-methylphenyl)-quinolin-4-yl)((2R,4S,5R)-5-vinylquinuclidin-2-yl)methanol-trifluoroacetate (8c)

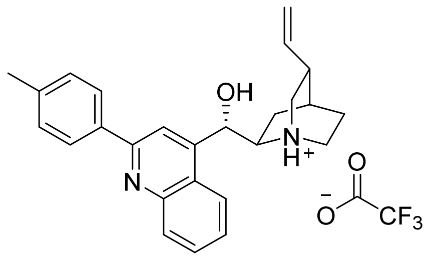

The title compound was synthesized following **General procedure I** starting with cinchonine (40.0 mg, 135.0 µmol, 1.0 eq) and 4-Methylphenylboronic acid (55.0 mg, 405 µmol, 3.0 eq). Purified using preparative HPLC (ACN/H_2O + 0.1 % TFA) to afford the product as an amorphous solid (14 mg, 23%).

^{1}H NMR (600 MHz, MeOD): δ = 8.27 (s, 1H), 8.21 (dd, J = 12.2, 8.4 Hz, 2H), 8.05 (d, J = 8.2 Hz, 2H), 7.89 (dd, J = 11.3, 4.1 Hz, 1H), 7.76 – 7.73 (m, 1H), 7.43 (d, J = 7.9 Hz, 2H), 6.17 (s, 1H), 6.16 – 6.09 (m, 1H), 5.30 (d, J = 10.4 Hz, 1H), 5.28 – 5.27 (m, 1H), 4.29 (tt, J = 15.1, 4.5 Hz, 1H), 3.77 (t, J = 9.5 Hz, 1H), 3.63 – 3.52 (m, 2H), 3.41 – 3.34 (m, 1H), 2.77 (q, J = 8.6 Hz, 1H), 2.52 (dd, J = 12.3, 10.7 Hz, 1H), 2.47 (s, 3H), 2.05 (s, 1H), 2.01 – 1.95 (m, 1H), 1.88 (dt, J = 11.3, 9.0 Hz, 1H), 1.32 (m, 1H); **^{13}C NMR** (151 MHz, MeOD): δ = 162.65 (d, J = 34.5 Hz), 158.40, 150.08, 147.87, 142.20, 138.04, 136.33, 132.03, 130.89, 129.52, 128.97, 128.83, 125.20, 123.73, 118.97, 118.32, 117.74, 117.04, 69.12, 61.62, 50.58, 50.12, 49.60, 38.34, 28.64, 23.96, 21.40, 19.12; **HRMS (ESI+)**: *m/z* = 385.2264 ([*M* + H]$^+$, calcd. for $C_{26}H_{29}ON_2^+$: 385.2274) (Δ = 2.80 ppm) – No TFA visible; $[\alpha]_{20}^{D}$= + 143° (c = 1.0, MeOH).

(*R*)-(2-(4-methylphenyl)-quinolin-4-yl)((2*S*,4*S*,5*R*)-5-vinylquinuclidin-2-yl)methanol-trifluoroacetate (12c)

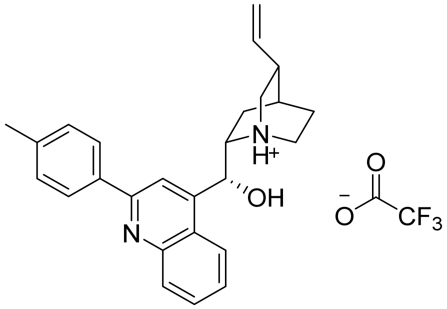

The title compound was synthesized following **General procedure I** starting with cinchonidine (40.0 mg, 135.0 µmol, 1.0 eq) and 4-Methylphenylboronic acid (55.0 mg, 405 µmol, 3.0 eq). Purified using preparative HPLC (ACN/H_2O + 0.1 % TFA) to afford the product as an amorphous solid (25 mg, 41%).

^{1}H NMR (700 MHz, MeOD): δ = 8.51 – 8.47 (m, 1H), 8.42 (d, J = 8.6 Hz, 1H), 8.14 (t, J = 7.8 Hz, 1H), 8.05 (d, J = 7.9 Hz, 2H), 7.98 (t, J = 7.7 Hz, 1H), 7.63 (d, J = 7.3 Hz, 1H), 7.56 (d, J = 7.9 Hz, 1H), 7.15 (d, J = 7.5 Hz, 1H), 6.27 (s, 1H), 5.78 (ddd, J = 17.2, 10.5, 6.8 Hz, 1H), 5.15 (d, J = 17.2 Hz, 1H), 5.06 (d, J = 10.6 Hz, 1H), 4.31 (dd, J = 15.0, 6.7 Hz, 1H), 3.78 (t, J = 8.8 Hz, 1H), 3.70 – 3.62 (m, 1H), 3.39 (m, 2H), 3.33 (s, 1H), 2.86 (s, 1H), 2.52 (s, 3H), 2.34 – 2.24 (m, 1H), 2.19 – 2.15 (m, 1H), 2.02 (t, J = 9.5 Hz, 1H), 1.69 – 1.62 (m, 1H); **^{13}C NMR** (176 MHz, MeOD): δ = 162.72 (q, J = 35.4 Hz), 156.96, 156.83, 145.13, 141.76, 141.47, 138.93, 135.07, 130.75, 129.98, 129.26, 125.34, 124.77, 124.25, 120.00, 118.64, 117.26, 68.76, 61.44, 55.55, 49.84, 45.46, 38.13, 27.89, 24.95 , 21.57, 19.28; **HRMS (ESI+)**: *m/z* = 385.2268 ([*M* + H]$^+$, calcd. for $C_{26}H_{29}ON_2^+$: 385.2274) (Δ = 1.71 ppm) – No TFA visible; $[\alpha]_{20}^{D}$= - 85° (c = 1.0, MeOH).

(S)-(2-(4-methylphenyl)-6-methoxyquinolin-4-yl)((2R,4S,5R)-5-vinylquinuclidin-2-yl)methanol- trifluoroacetate (7b)

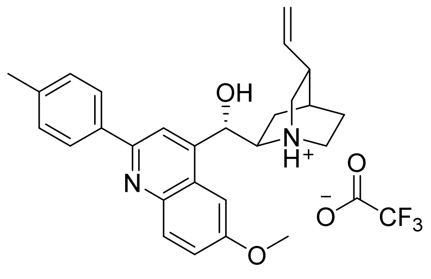

The title compound was synthesized following **General procedure I** starting with quinidine (40.0 mg, 120.0 µmol, 1.0 eq) and 4-Methylphenylboronic acid (49.0 mg, 360 µmol, 3.0 eq). Purified using preparative HPLC (ACN/H_2O + 0.1 % TFA) to afford the product as an amorphous solid (28 mg, 44%).

^{1}H NMR (700 MHz, MeOD): δ = 8.35 (s, 1H), 8.24 (d, J = 9.3 Hz, 1H), 7.98 (d, J = 7.8 Hz, 2H), 7.70 (d, J = 9.5 Hz, 1H), 7.61 (s, 1H), 7.50 (d, J = 7.8 Hz, 2H), 6.30 (s, J = 10.6 Hz, 1H), 6.14 (ddd, J = 16.6, 9.5, 7.3 Hz, 1H), 5.33 – 5.25 (m, 2H), 4.29 – 4.23 (m, 1H), 4.07 (s, 3H), 3.77 (t, J = 9.6 Hz, 1H), 3.56 (t, J = 11.5 Hz, 2H), 3.37 (dd, J = 21.4, 10.5 Hz, 1H), 2.77 (dd, J = 16.9, 8.3 Hz, 1H), 2.58 – 2.52 (m, 1H), 2.48 (s, 3H), 2.05 (s, 1H), 1.98 (t, J = 10.6 Hz, 1H), 1.90 – 1.85 (m, 1H); **^{13}C NMR** (176 MHz, MeOD): δ = 162.68 (q, J = 35.6 Hz), 161.41, 154.11, 143.94, 138.12, 138.08, 131.34, 130.56, 130.11, 129.46, 129.31, 129.21, 127.07, 127.02, 126.77, 119.68, 117.71, 102.94, 69.05, 61.02, 57.12, 50.48, 49.76, 49.37, 38.34, 28.71, 24.00, 21.53, 18.89; **HRMS (ESI+)**: *m/z* = 415.2369 ([M + H]$^+$, calcd. for $C_{27}H_{31}O_2N_2^+$: 415.2380) (Δ = 2.76 ppm) – No TFA visible; $[\alpha]_{20}^{D}$= + 115° (c = 1.0, MeOH).

(*R*)-(2-(4-methylphenyl)-6-methoxyquinolin-4-yl)((2*S*,4*S*,5*R*)-5-vinylquinuclidin-2-yl)methanol- trifluoroacetate (11c)

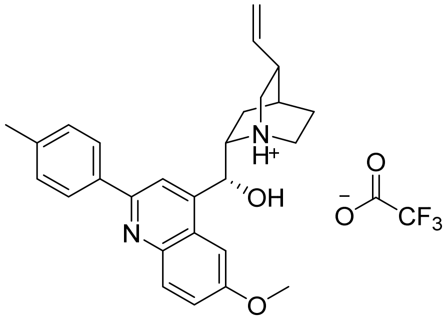

The title compound was synthesized following **General procedure I** starting with quinine (40.0 mg, 120.0 µmol, 1.0 eq) and 4-Chlorophenylboronic acid (49.0 mg, 360 µmol, 3.0 eq). Purified using preparative HPLC (ACN/H_2O + 0.1 % TFA) to afford the product as an amorphous solid (21 mg, 33%).

^{1}H NMR (600 MHz, MeOD): δ = 8.25 (s, 1H), 8.12 (d, J = 9.3 Hz, 1H), 7.92 (d, J = 8.2 Hz, 2H), 7.55 (dd, J = 9.3, 2.5 Hz, 1H), 7.47 (d, J = 2.2 Hz, 1H), 7.38 (d, J = 8.0 Hz, 2H), 6.11 (s, 1H), 5.73 (ddd, J = 17.4, 10.4, 7.2 Hz, 1H), 5.08 (d, J = 17.1 Hz, 1H), 4.98 (d, J = 10.4 Hz, 1H), 4.26 – 4.19 (m, 1H), 3.99 (s, 3H), 3.72 – 3.66 (m, 1H), 3.59 (dd, J = 12.9, 10.8 Hz, 1H), 3.29 (dt, J = 7.9, 6.3 Hz, 2H), 2.78 (s, 1H), 2.40 (s, J = 6.9 Hz, 3H), 2.27 – 2.14 (m, 2H), 2.07 (d, J = 2.8 Hz, 1H), 1.92 (t, J = 9.7 Hz, 1H), 1.60 – 1.55 (m, 1H); **^{13}C NMR** (126 MHz, MeOD): δ = 163.13 (q, J = 34.7 Hz), 160.77, 154.63, 151.63, 142.69, 140.78, 139.09, 134.04, 130.55, 1630.03, 129.86, 128.97, 128.42, 126.57, 125.65, 119.22, 117.06, 102.38, 68.40, 61.03, 56.82, 55.34, 45.13, 38.38, 28.14, 24.99, 21.45, 19.17; **HRMS (ESI+)**: *m/z* = 415.2377 ([*M* + H]$^+$, calcd. for $C_{27}H_{31}O_2N_2^+$: 415.2380) (Δ = 0.66 ppm) – No TFA visible; $[\boldsymbol{\alpha}]_{\mathbf{20}}^{\boldsymbol{D}}$= - 61 °(c = 1.0, MeOH).

(S)-(2-phenyl-quinolin-4-yl)((2R,4S,5R)-5-vinylquinuclidin-2-yl)methanol-trifluoroacetate (8d)

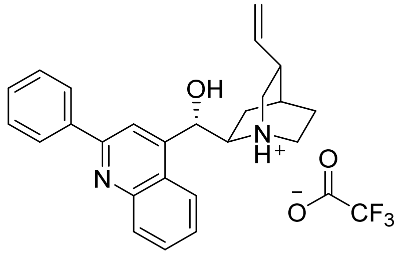

The title compound was synthesized following **General procedure I** starting with cinchonine (40.0 mg, 135.0 µmol, 1.0 eq) and phenylboronic acid (49.0 mg, 405 µmol, 3.0 eq). Purified using preparative HPLC (ACN/H_2O + 0.1 % TFA) to afford the product as an amorphous solid (16 mg, 27%).

^{1}H NMR (700 MHz, MeOD): δ = 8.29 (s, 1H), 8.25 (t, J = 7.3 Hz, 2H), 8.15 (dd, J = 5.2, 3.3 Hz, 2H), 7.92 – 7.89 (m, 1H), 7.78 – 7.75 (m, 1H), 7.61 (t, J = 7.3 Hz, 2H), 7.58 (t, J = 7.2 Hz, 1H), 6.22 (d, J = 1.4 Hz, 1H), 6.14 (ddd, J = 17.5, 10.4, 7.3 Hz, 1H), 5.32 – 5.26 (m, 2H), 4.30 (ddd, J = 12.6, 8.5, 2.4 Hz, 1H), 3.77 (t, J = 9.5 Hz, 1H), 3.63 – 3.53 (m, 2H), 3.41 – 3.35 (m, 1H), 2.77 (dd, J = 17.5, 8.6 Hz, 1H), 2.56 – 2.50 (m, 1H), 2.05 (s, 1H), 2.01 – 1.95 (m, 1H), 1.88 (ddd, J = 12.7, 10.3, 1.4 Hz, 1H), 1.35 – 1.31 (m, 1H); **^{13}C NMR** (176 MHz, MeOD): δ = 162.78 (q, J = 35.6 Hz), 158.95 , 158.39, 150.27, 147.95, 139.24, 138.07, 132.04, 131.48, 130.20, 129.65, 129.04, 128.99, 125.32, 123.86, 118.43, 117.71, 69.05, 61.61, 50.55, 50.05, 49.53, 38.34, 28.66, 23.97, 19.08; **HRMS (ESI+)**: *m/z* = 371.2117 ([*M* + H]$^+$, calcd. for $C_{25}H_{27}ON_2^+$: 371.2118) (Δ = 0.19 ppm) – No TFA visible; $[\alpha]_{20}^{D}$= + 80° (c = 1.0, MeOH).

(*R*)-(2-phenyl-quinolin-4-yl)((2*S*,4*S*,5*R*)-5-vinylquinuclidin-2-yl)methanol-trifluoroacetate (12d) *and* (*R*)-(7-phenyl-quinolin-4-yl)((2*S*,4*S*,5*R*)-5-vinylquinuclidin-2-yl)methanol- trifluoroacetate (13c)

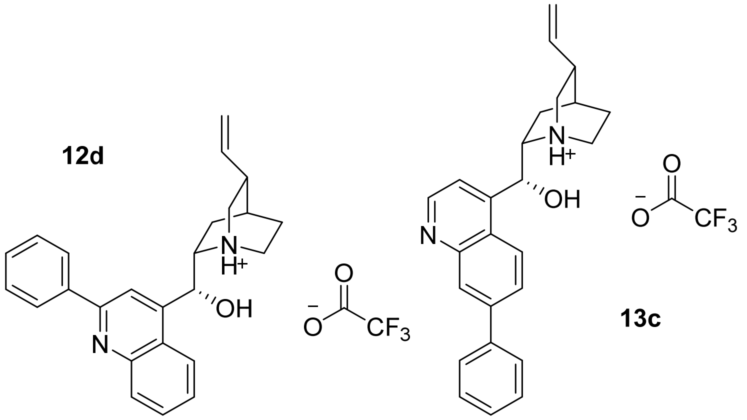

12d **13c**

The title compound was synthesized following **General procedure I** starting with cinchonidine (40.0 mg, 135.0 µmol, 1.0 eq) and phenylboronic acid (55.0 mg, 405 µmol, 3.0 eq). Purified using preparative HPLC (ACN/H_2O + 0.1 % TFA) to afford the products **12d** (19 mg, 33%) and **13d** (4 mg, 7%) as an amorphous solids.

12d: ^{1}H NMR (500 MHz, MeOD): δ = 8.30 (s, 1H), 8.27 – 8.22 (m, 2H), 8.11 (dt, J = 4.2, 2.3 Hz, 2H), 7.94 – 7.90 (m, 1H), 7.77 (ddd, J = 8.2, 7.0, 1.1 Hz, 1H), 7.63 – 7.57 (m, 3H), 6.19 (s, 1H), 6.11 (ddd, J = 17.5, 10.4, 7.3 Hz, 1H), 5.29 – 5.22 (m, 2H), 4.26 (ddd, J = 12.5, 8.4, 2.4 Hz, 1H), 3.75 (t, J = 9.5 Hz, 1H), 3.55 (dt, J = 22.5, 11.1 Hz, 2H), 3.39 – 3.31 (m, 1H), 2.74 (dd, J = 17.5, 8.6 Hz, 1H), 2.53 – 2.45 (m, 1H), 2.02 (s, 1H), 1.98 – 1.92 (m, 1H), 1.88 – 1.81 (m, 1H), 1.32 – 1.25 (m, 1H); **^{13}C NMR** (126 MHz, MeOD): δ = 162.28 (d, 35.98 Hz), 158.11, 151.63, 146.78, 138.26, 138.06, 132.67, 131.95, 130.34, 129.39, 129.25, 128.64, 125.40, 124.07, 118.77, 117.74, 69.09, 61.52, 50.54, 50.06, 49.63, 38.35, 28.65, 23.95, 19.05; **HRMS (ESI+)**: *m/z* = 371.2118 ([*M* + H]$^+$, calcd. for $C_{25}H_{27}ON_2^+$: 371.2118) (Δ = 0.09 ppm) – No TFA visible; $[\boldsymbol{\alpha}]_{\mathbf{20}}^{\boldsymbol{D}}$= + 38° (c = 1.0, MeOH).

13c: ^{1}H NMR (500 MHz, MeOD): δ = 8.99 (dd, J = 26.5, 4.8 Hz, 1H), 8.40 – 8.33 (m, 1H), 8.22 (m, 1H), 8.14 (dd, J = 8.9, 1.6 Hz, 1H), 7.99 (t, J = 4.7 Hz, 1H), 7.82 (dd, J = 7.1, 5.5 Hz, 2H), 7.57 – 7.51 (m, 2H), 7.46 (dt, J = 14.9, 7.5 Hz, 1H), 6.24 – 6.16 (m, 1H), 6.16 – 6.05 (m, 1H), 5.31 – 5.23 (m, 2H), 4.32 – 4.24 (m, 1H), 4.03 – 3.96 (m, 1H), 3.79 – 3.69 (m, 1H), 3.63 – 3.47 (m, 1H), 3.36 (dd, J = 20.7, 10.4 Hz, 1H), 2.75 (s, 1H), 2.55 – 2.43 (m, 1H), 2.05 – 1.98 (m, 1H), 1.89 (ddd, J = 19.6, 16.2, 10.7 Hz, 2H), 1.34 – 1.19 (m, 1H); **HRMS (ESI+)**: *m/z* =

371.2117 ($[M + H]^+$, calcd. for $C_{25}H_{27}ON_2^+$: 371.2118) (Δ = 0.28 ppm) – No TFA visible; $[\alpha]_{20}^{D}$= + 45° (c = 1.0, MeOH).

(S)-(2-phenyl-6-methoxyquinolin-4-yl)((2R,4S,5R)-5-vinylquinuclidin-2-yl)methanol-trifluoroacetate (7c) ***and*** **(S)-(5-(phenyl)-6-methoxyquinolin-4-yl)((2R,4S,5R)-5-vinylquinuclidin-2-yl)methanol- trifluoroacetate (9d)**

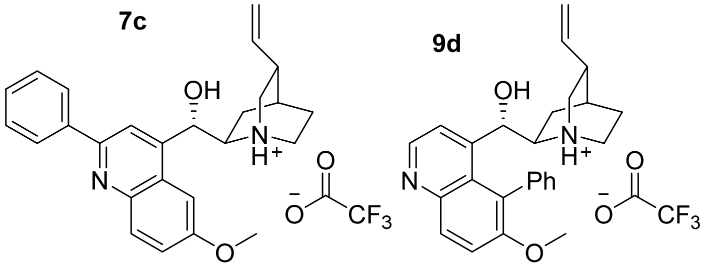

The title compound was synthesized following **General procedure I** starting with quinidine (40.0 mg, 120.0 µmol, 1.0 eq) and Phenylboronic acid (44.0 mg, 360 µmol, 3.0 eq). Purified using preparative HPLC (ACN/H_2O + 0.1 % TFA) to afford the products **7c** (27 mg, 44%) and **9d** (6 mg, 10%) as amorphous solids.

7c:^{1}H NMR (500 MHz, MeOD): δ = 8.31 (s, 1H), 8.19 (d, J = 9.3 Hz, 1H), 8.05 – 8.00 (m, 2H), 7.67 – 7.59 (m, 4H), 7.55 (d, J = 2.6 Hz, 1H), 6.26 (s, 1H), 6.10 (ddd, J = 17.5, 10.4, 7.3 Hz, 1H), 5.27 – 5.19 (m, 2H), 4.22 (ddd, J = 12.6, 8.4, 2.4 Hz, 1H), 4.02 (s, 3H), 3.72 (t, J = 9.5 Hz, 1H), 3.51 (dd, J = 12.2, 11.0 Hz, 2H), 3.36 – 3.28 (m, 1H), 2.72 (dd, J = 17.5, 8.6 Hz, 1H), 2.54 – 2.47 (m, 1H), 2.00 (s, 1H), 1.96 – 1.89 (m, 1H), 1.87-1.80 (m, 1H), 1.31 – 1.24 (m, 1H); **^{13}C NMR** (126 MHz, MeOD): δ = 162.39 (q, J = 35.8 Hz), 161.26, 154.03, 153.53, 139.15, 137.97, 135.49, 132.50, 130.45, 129.32, 127.05, 126.84, 118.83, 119.58, 117.54, 116.51, 102.59, 68.86, 60.83, 56.92, 50.27, 49.57, 38.19, 28.54, 23.83, 18.71; **HRMS (ESI+)**: *m/z* = 401.2222 ($[M + H]^+$, calcd. for $C_{26}H_{29}O_2N_2^+$: 401.2224) (Δ = 0.47 ppm) – No TFA visible; $[\alpha]_{20}^{D}$= + 118° (c = 1.0, MeOH).

9d:^{1}H NMR (500 MHz, MeOD): δ = 8.92 (t, J = 7.2 Hz, 1H), 8.13 (d, J = 5.4 Hz, 1H), 8.07 (s, 1H), 7.60 (ddd, J = 7.7, 6.3, 5.3 Hz, 3H), 7.50 – 7.40 (m, 3H), 6.29 (s, J = 17.9 Hz, 1H), 6.10 (dddd, J = 17.6, 10.3, 7.3, 3.0 Hz, 1H), 5.32 – 5.21 (m, 2H), 4.26 (ddd, J = 12.4, 8.4, 2.2 Hz, 1H), 4.07 (s, J = 7.4 Hz, 3H), 3.76 (dd, J = 19.3, 9.7 Hz, 1H), 3.55 (t, J = 11.5 Hz, 2H), 3.43 – 3.32 (m, 1H), 2.76 (dd, J = 17.4, 8.5 Hz, 1H), 2.54 – 2.44 (m, 1H), 2.04 (s, 1H), 1.95 (dd, J = 14.4, 10.2 Hz, 1H), 1.92 – 1.83 (m, 1H), 1.27 (dtd, J = 13.4, 9.4, 3.9 Hz, 1H); **^{13}C NMR** (126

MHz, MeOD): δ = 162.54 (d, J = 37.0 Hz), 159.36, 153.89, 144.20, 141.63, 138.06, 137.36, 131.20, 130.62, 129.85, 129.45, 127.76, 126.08, 120.69, 117.75, 102.64, 68.99, 60.91, 57.37, 50.47, 49.80, 49.63, 38.28, 28.69, 23.96, 18.92; **HRMS (ESI+)**: *m/z* = 401.2218([*M* + H]$^+$, calcd. for $C_{26}H_{29}O_2N_2^+$: 401.2224) (Δ = 1.36 ppm) – No TFA visible; $[\alpha]_{20}^{D}$= + 86° (c = 1.0, MeOH).

(*R*)-(2-phenyl-6-methoxyquinolin-4-yl)((2*S*,4*S*,5*R*)-5-vinylquinuclidin-2-yl)methanol-trifluoroacetate (11d)

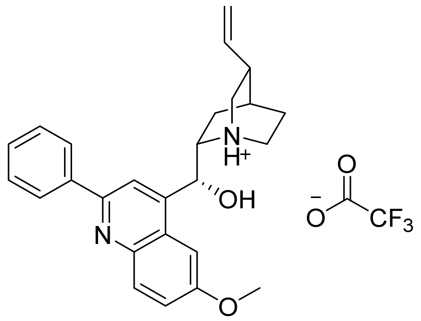

The title compound was synthesized following **General procedure I** starting with quinine (40.0 mg, 120.0 µmol, 1.0 eq) and Phenylboronic acid (44.0 mg, 360 µmol, 3.0 eq). Purified using preparative HPLC (ACN/H_2O + 0.1 % TFA) to afford the product as an amorphous solid (24 mg, 39%).

^{1}H NMR (500 MHz, MeOD): δ = 8.33 (s, 1H), 8.19 (d, J = 9.3 Hz, 1H), 8.07 – 8.03 (m, 2H), 7.65 – 7.59 (m, 4H), 7.54 (d, J = 2.5 Hz, 1H), 6.15 (s, 1H), 5.76 (ddd, J = 17.4, 10.4, 7.1 Hz, 1H), 5.10 (d, J = 17.1 Hz, 1H), 5.01 (d, J = 10.4 Hz, 1H), 4.28 – 4.19 (m, 1H), 4.03 (s, 3H), 3.75 – 3.69 (m, 1H), 3.62 (dd, J = 13.0, 10.7 Hz, 1H), 3.34 – 3.28 (m, 2H), 2.80 (d, J = 7.2 Hz, 1H), 2.31 – 2.17 (m, 2H), 2.09 (dd, J = 5.9, 2.9 Hz, 1H), 1.98 – 1.91 (m, 1H), 1.65 – 1.57 (m, 1H); **^{13}C NMR** (126 MHz, MeOD): δ = 162.48 (q, J = 35.4 Hz), 161.12, 154.34, 152.72, 139.99, 139.08, 136.20, 132.20, 130.35, 129.20, 127.83, 126.98, 126.33, 119.65, 118.87, 117.09, 116.55, 102.53, 68.49, 61.02, 56.90, 55.38, 45.17, 38.38, 28.14, 24.99, 19.20; **HRMS (ESI+)**: *m/z* = 401.2222 ([*M* + H]$^+$, calcd. for $C_{26}H_{29}O_2N_2^+$: 401.2224) (Δ = 0.44 ppm) – No TFA visible; $[\alpha]_{20}^{D}$= + 27° (c = 1.0, MeOH).

(S)-(2-(3-bromophenyl)-quinolin-4-yl)((2R,4S,5R)-5-vinylquinuclidin-2-yl)methanol-trifluoroacetate (8e)

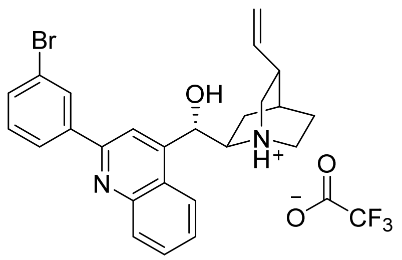

The title compound was synthesized following **General procedure I** starting with quinidine (40.0 mg, 120.0 µmol, 1.0 eq) and 3-Bromophenylboronic acid (72.0 mg, 360 µmol, 3.0 eq). Purified using preparative HPLC (ACN/H_2O + 0.1 % TFA) to afford the product as an amorphous solid (12 mg, 18%).

^{1}H NMR (700 MHz, MeOD): δ = 8.39 (t, J = 1.8 Hz, 1H), 8.25 (s, 1H), 8.23 (d, J = 7.9 Hz, 1H), 8.18 (d, J = 8.3 Hz, 1H), 8.16 – 8.14 (m, 1H), 7.87 (ddd, J = 8.3, 6.9, 1.1 Hz, 1H), 7.74 (ddd, J = 8.2, 6.9, 1.1 Hz, 1H), 7.70 (ddd, J = 8.0, 1.9, 0.8 Hz, 1H), 7.52 (t, J = 7.9 Hz, 1H), 6.19 – 6.12 (m, 2H), 5.32 – 5.27 (m, 2H), 4.30 (ddd, J = 12.5, 8.5, 2.4 Hz, 1H), 3.78 (t, J = 9.5 Hz, 1H), 3.58 (ddd, J = 32.1, 17.9, 11.0 Hz, 2H), 3.43 – 3.35 (m, 1H), 2.78 (dd, J = 17.6, 8.6 Hz, 1H), 2.58 – 2.51 (m, 1H), 2.06 (s, J = 12.8 Hz, 1H), 2.02 – 1.96 (m, 1H), 1.92 – 1.85 (m, 1H), 1.36 – 1.29 (m, 1H); **^{13}C NMR** (176 MHz, MeOD): δ = 162.33 (q, J = 36.2 Hz), 156.79, 149.31, 148.87, 142.46, 138.05, 133.83, 131.85, 131.79, 131.28, 131.15, 128.91, 127.45, 125.42, 124.12, 123.49, 117.90, 117.51, 69.16, 61.71, 50.58, 50.13, 49.53, 38.37, 28.63, 23.97, 19.18; **HRMS (ESI+)**: *m/z* = 451.1199 ([*M* + H]$^+$, calcd. for $C_{25}H_{26}ON_2$ $^{81}Br^+$: 451.1203) (Δ = 0.75 ppm) – No TFA visible; $[\alpha]_{20}^{D}$= +69° (c = 1.0, MeOH).

(*R*)-(2-(3-bromophenyl)-quinolin-4-yl)((2*S*,4*S*,5*R*)-5-vinylquinuclidin-2-yl)methanol-trifluoroacetate (12e)

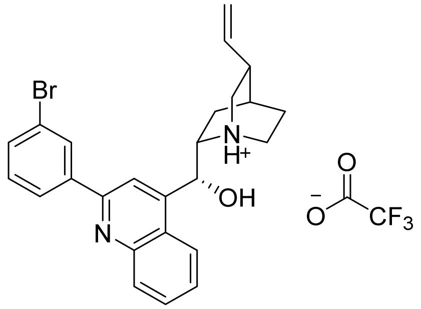

The title compound was synthesized following **General procedure I** starting with cinchonidine (40.0 mg, 120.0 µmol, 1.0 eq) and 3-Bromophenylboronic acid (81.0 mg, 405 µmol, 3.0 eq). Purified using preparative HPLC (ACN/H_2O + 0.1 % TFA) to afford the product as an amorphous solid (19 mg, 27%).

^{1}H NMR (700 MHz, MeOD): δ = 8.38 (s, 1H), 8.27 (s, 1H), 8.22 (t, J = 9.2 Hz, 2H), 8.15 (d, J = 7.8 Hz, 1H), 7.89 – 7.86 (m, 1H), 7.76 – 7.72 (m, 1H), 7.71 – 7.69 (m, 1H), 7.51 (t, J = 7.9 Hz, 1H), 6.08 (d, J = 1.4 Hz, 1H), 5.79 (ddd, J = 17.3, 10.5, 7.0 Hz, 1H), 5.15 (d, J = 17.2 Hz, 1H), 5.05 (d, J = 10.5 Hz, 1H), 4.34 – 4.28 (m, 1H), 3.79 – 3.75 (m, 1H), 3.65 (dd, J = 13.0, 10.7 Hz, 1H), 3.40 – 3.33 (m, 2H), 2.84 (dd, J = 3.4, 1.8 Hz, 1H), 2.34 – 2.24 (m, 2H), 2.15 (dd, J = 6.1, 3.0 Hz, 1H), 1.99 (tdd, J = 10.9, 5.2, 2.8 Hz, 1H), 1.67 – 1.61 (m, 1H); **^{13}C NMR** (176 MHz, MeOD): δ = 162.53 (q, J = 36.1 Hz), 156.99, 149.34, 149.07, 142.53, 139.38, 134.13, 131.88, 131.63, 131.60, 130.95, 129.21, 127.76, 125.65, 124.36, 123.81, 118.15, 117.50, 69.08, 62.10, 55.86, 49.77, 45.82, 38.77, 28.42, 25.41, 19.80; **HRMS (ESI+)**: *m/z* = 451.1202 ($[M + H]^+$, calcd. for $C_{25}H_{26}ON_2$ $^{81}Br^+$: 451.1203) (Δ = 0.18 ppm) – No TFA visible; $[\alpha]_{20}^{D}$= -33° (c = 1.0, MeOH).

(S)-(2-(3-bromophenyl)-6-methoxyquinolin-4-yl)((2R,4S,5R)-5-vinylquinuclidin-2-yl)methanol- trifluoroacetate (7d)

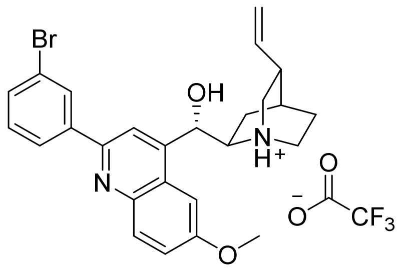

The title compound was synthesized following **General procedure I** starting with quinidine (40.0 mg, 120.0 µmol, 1.0 eq) and 3-Bromophenylboronic acid (72.0 mg, 360 µmol, 3.0 eq). Purified using preparative HPLC (ACN/H_2O + 0.1 % TFA) to afford the product as an amorphous solid (22 mg, 31%).

^{1}H NMR (700 MHz, MeOD): δ = 8.34 (t, J = 1.8 Hz, 1H), 8.20 (s, 1H), 8.14 (d, J = 9.2 Hz, 1H), 8.12 – 8.09 (m, 1H), 7.67 (ddd, J = 8.0, 1.9, 0.8 Hz, 1H), 7.54 (dd, J = 9.2, 2.6 Hz, 1H), 7.50 (t, J = 7.9 Hz, 1H), 7.38 (d, J = 2.6 Hz, 1H), 6.16 (ddd, J = 17.6, 10.5, 7.3 Hz, 1H), 6.07 (s, 1H), 5.30 (dd, J = 12.8, 5.5 Hz, 2H), 4.30 (ddd, J = 12.5, 8.4, 2.4 Hz, 1H), 4.04 (s, 3H), 3.77 (t, J = 9.5 Hz, 1H), 3.58 (dt, J = 18.9, 6.7 Hz, 2H), 3.43 – 3.36 (m, 1H), 2.79 (dd, J = 17.5, 8.6 Hz, 1H), 2.59 – 2.54 (m, 1H), 2.08 (s, 1H), 2.06 – 1.98 (m, 1H), 1.95 – 1.90 (m, 1H), 1.37 (ddt, J = 22.8, 13.9, 5.6 Hz, 3H), 1.32 – 1.29 (m, 2H); **^{13}C NMR** (176 MHz, MeOD): δ = 162.53 (d, J = 37.3 Hz), 160.34, 154.24, 147.27, 145.25, 142.58, 138.07, 133.41, 132.57, 131.79, 131.30, 127.16, 126.54, 124.08, 123.78, 117.85, 117.75, 101.96, 69.29, 61.49, 56.51, 50.53, 49.96, 49.53, 38.36, 28.63, 24.01, 19.17; **HRMS (ESI+)**: *m/z* = 481.1305 ($[M + H]^+$, calcd. for $C_{26}H_{28}O_2N_2\,^{81}Br^+$:481.1308) (Δ = 0.67 ppm) – No TFA visible; $[\boldsymbol{\alpha}]_{\mathbf{20}}^{\boldsymbol{D}}$= +70° (c = 1.0, MeOH).

(*R*)-(2-(3-bromophenyl)-6-methoxyquinolin-4-yl)((2*S*,4*S*,5*R*)-5-vinylquinuclidin-2-yl)methanol- trifluoroacetate (11e)

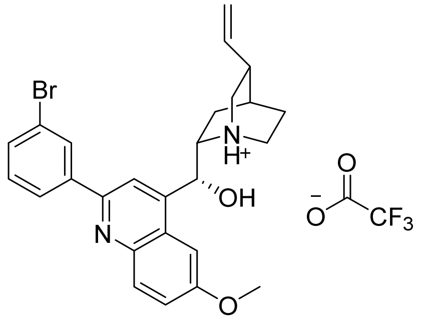

The title compound was synthesized following **General procedure I** starting with quinine (40.0 mg, 120.0 µmol, 1.0 eq) and 3-Bromophenylboronic acid (72.0 mg, 360 µmol, 3.0 eq). Purified using preparative HPLC (ACN/H_2O + 0.1 % TFA) to afford the product as an amorphous solid (17 mg, 24%).

^{1}H NMR (700 MHz, MeOD): δ = 8.32 (t, J = 1.8 Hz, 1H), 8.25 (s, J = 8.7 Hz, 1H), 8.14 (d, J = 9.2 Hz, 1H), 8.09 (d, J = 7.9 Hz, 1H), 7.70 – 7.67 (m, 1H), 7.55 (dd, J = 9.2, 2.6 Hz, 1H), 7.50 (t, J = 7.9 Hz, 1H), 7.46 (d, J = 2.6 Hz, 1H), 6.09 (d, J = 1.1 Hz, 1H), 5.80 (ddd, J = 17.3, 10.5, 7.1 Hz, 1H), 5.15 (dd, J = 17.1, 4.4 Hz, 1H), 5.06 (d, J = 10.5 Hz, 1H), 4.32 – 4.27 (m, 1H), 4.05 (s, 3H), 3.76 – 3.72 (m, 1H), 3.66 (dd, J = 13.1, 10.7 Hz, 1H), 3.35 (ddd, J = 13.1, 6.9, 3.1 Hz, 2H), 2.84 (dd, J = 3.4, 1.8 Hz, 1H), 2.34 – 2.30 (m, 1H), 2.29 – 2.23 (m, 1H), 2.15 (dd, J = 6.0, 3.0 Hz, 1H), 2.02 – 1.96 (m, 1H), 1.67 – 1.61 (m, 1H); **^{13}C NMR** (176 MHz, MeOD): δ = 162.33 (q, J = 35.3 Hz)), 160.81, 153.94, 149.02, 144.15, 141.74, 139.35, 133.91, 132.00, 131.87, 131.49, 128.33, 127.54, 126.87, 124.32 (d, J = 72.4 Hz), 118.44 117.23, 102.27, 68.87, 61.55, 56.95, 55.70, 49.65, 45.53, 38.67, 28.38, 25.30, 19.64; **HRMS (ESI+)**: *m/z* = 481.1303 ([M + H]$^+$, calcd. for $C_{26}H_{28}O_2N_2$ $^{81}Br^+$: 481.1308) (Δ = 1.12 ppm) – No TFA visible; $[\alpha]_{20}^{D}$= -11° (c = 1.0, MeOH).

(S)-(2-(3-chlorophenyl)-6-methoxyquinolin-4-yl)((2R,4S,5R)-5-vinylquinuclidin-2-yl)methanol- trifluoroacetate (7e) ***and*** **(S)-(5-(3-chlorophenyl)-6-methoxyquinolin-4-yl)((2R,4S,5R)-5-vinylquinuclidin-2-yl)methanol- trifluoroacetate (9f)**

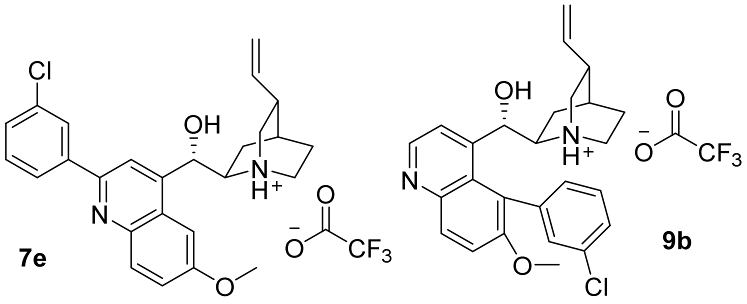

7e **9b**

The title compound was synthesized following **General procedure I** starting with quinidine (40.0 mg, 120.0 µmol, 1.0 eq) and 3-Chlorophenylboronic acid (56.0 mg, 360 µmol, 3.0 eq). Purified using preparative HPLC (ACN/H_2O + 0.1 % TFA) to afford **7e** (20 mg, 30%) and **9b** (4 mg, 6%) as amorphous solids.

7e: ^{1}**H NMR** (500 MHz, MeOD): δ = 8.16 (s, 1H), 8.13 (t, J = 1.6 Hz, 1H), 8.08 (d, J = 9.3 Hz, 1H), 8.01 (d, J = 7.6 Hz, 1H), 7.53 (d, J = 7.9 Hz, 1H), 7.51 – 7.46 (m, 2H), 7.34 (d, J = 2.4 Hz, 1H), 6.12 (ddd, J = 17.5, 10.5, 7.4 Hz, 1H), 6.05 (s, 1H), 5.27 – 5.22 (m, 2H), 4.29 – 4.22 (m, 1H), 3.99 (s, 3H), 3.71 (t, J = 9.4 Hz, 1H), 3.56 – 3.49 (m, 2H), 3.39 – 3.30 (m, 1H), 2.74 (dd, J = 17.4, 8.6 Hz, 1H), 2.55 – 2.48 (m, 1H), 2.01 (d, J = 10.7 Hz, 1H), 1.93 (dd, J = 7.5, 4.6 Hz, 1H), 1.90 – 1.81 (m, 1H), 1.29 (ddd, J = 12.4, 9.9, 6.7 Hz, 1H); ^{13}**C NMR** (126 MHz, MeOD): δ = 160.38, 154.26, 147.55, 145.06, 142.23, 138.12, 136.09, 132.38, 131.59, 130.48, 128.37, 126.78, 126.58, 123.97, 117.91, 117.72, 101.89, 69.21, 61.40, 56.53, 50.48, 49.90, 38.39, 28.66, 24.01, 19.10; **HRMS (ESI+)**: *m/z* = 437.1803 ([*M* + H]$^{+}$, calcd. for $C_{26}H_{28}O_2N_2$ $^{37}Cl^{+}$:437.1804) (Δ = 0.20 ppm) – No TFA visible; $[\alpha]_{20}^{D}$= - 41° (c = 1.0, MeOH).

9b: ^{1}**H NMR** (500 MHz, MeOD): δ = 8.88 – 8.84 (m, 1H), 8.25 (dd, J = 9.4, 1.9 Hz, 1H), 8.15 (dd, J = 4.7, 2.0 Hz, 1H), 7.90 (d, J = 9.4 Hz, 1H), 7.79 (m, 1H), 7.54 (m, 3H), 7.18 (s, 1H), 7.09 (t, J = 8.8 Hz, 1H), 5.84 (ddd, J = 17.3, 10.5, 6.9 Hz, 1H), 5.14 (m, 3H), 3.96 – 3.88 (m, 1H), 3.84 (d, J = 7.4 Hz, 3H), 3.33 (m, 1H), 3.24 – 3.16 (m, 1H), 3.14 – 3.07 (m, 1H), 2.60 – 2.52 (m, 1H), 1.89 – 1.75 (m, 4H), 1.34 – 1.22 (m, 1H), 0.69 – 0.59 (m, 1H); ^{13}**C NMR** (126 MHz, MeOD): δ = 158.55, 150.88, 146.29, 140.43, 137.52, 135.37, 132.11, 131.77, 131.20, 130.72, 130.52, 130.30, 130.14, 126.54, 123.64, 122.84, 119.49, 117.70, 69.16, 60.09, 57.37, 51.07, 49.70, 37.92, 28.61, 23.30, 19.80; **HRMS (ESI+)**: *m/z* = 437.1802 ([*M* + H]$^{+}$, calcd. for $C_{26}H_{28}O_2N_2$ $^{37}Cl^{+}$:437.1804) (Δ = 0.46 ppm) – No TFA visible; $[\alpha]_{20}^{D}$= + 12° (c = 1.0, MeOH).

(S)-(2-(3-chloro,4-fluorophenyl)-6-methoxyquinolin-4-yl)((2R,4S,5R)-5-vinylquinuclidin-2-yl)methanol- trifluoroacetate (7f) *and* (S)-(5-(3-chloro,4-fluorophenyl)-6-methoxyquinolin-4-yl)((2R,4S,5R)-5-vinylquinuclidin-2-yl)methanol-trifluoroacetate (9c)

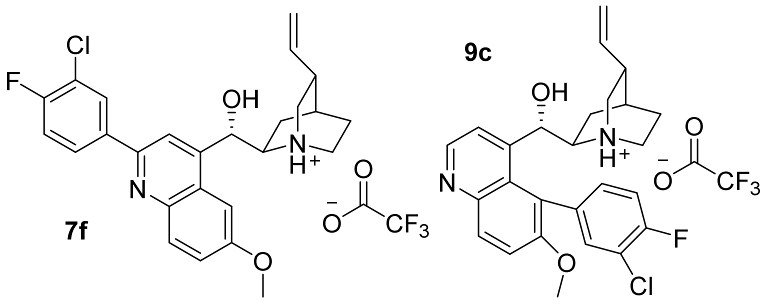

The title compound was synthesized following **General procedure I** starting with quinidine (40.0 mg, 120.0 µmol, 1.0 eq) and (3-Chloro,4-Fluoro)phenylboronic acid (56.0 mg, 360 µmol, 3.0 eq). Purified using preparative HPLC (ACN/H_2O + 0.1 % TFA) to **7f** (10 mg, 15%) and **9c** (3 mg, 5%) as amorphous solids.

7f: **^{1}H NMR** (500 MHz, MeOD): δ = 8.15 (s, 1H), 8.12 – 8.05 (m, 2H), 7.48 (dd, J = 9.3, 2.5 Hz, 1H), 7.40 (t, J = 8.8 Hz, 1H), 7.35 – 7.28 (m, 2H), 6.12 (ddd, J = 17.5, 10.4, 7.4 Hz, 1H), 6.02 (s, 1H), 5.28 – 5.22 (m, 2H), 4.29 – 4.20 (m, 1H), 3.99 (s, 3H), 3.72 (t, J = 9.4 Hz, 1H), 3.57 – 3.49 (m, 2H), 3.35 (m, 1H), 2.74 (dd, J = 17.4, 8.6 Hz, 1H), 2.57 – 2.48 (m, 1H), 2.04 – 1.99 (m, 1H), 1.99 – 1.91 (m, 1H), 1.91 – 1.82 (m, 1H), 1.35 – 1.23 (m, 1H); **^{13}C NMR** (176 MHz, MeOD): δ = 162.98 (q, J = 34.5 Hz), 160.91, 160.29, 159.48, 153.46, 147.09, 145.45, 138.12, 138.10, 132.77, 130.63, 128.68, 128.63, 126.40, 123.68, 122.55, 122.45, 118.16, 118.03, 117.72, 117.46, 101.93, 69.29, 61.50, 56.49, 50.53, 49.97, 49.53, 38.39, 28.66, 24.02, 19.18; **^{19}F NMR** (377 MHz, MeOD): δ = -77.05, -118.64; **HRMS (ESI+)**: *m/z* = 455.1706 ([*M* + H]$^+$, calcd. for $C_{26}H_{27}O_2N_2{}^{37}ClF^+$: 455.1710) (Δ = 0.88 ppm) – No TFA visible; $[\alpha]_{20}^{D}$= (c = 1.0, MeOH).

9c: **^{1}H NMR** (500 MHz, MeOD): δ = 8.81 (dd, J = 4.7, 2.7 Hz, 1H), 8.22 (dd, J = 9.3, 2.4 Hz, 1H), 8.06 (dd, J = 9.2, 4.6 Hz, 1H), 7.83 (d, J = 9.4 Hz, 1H), 7.46 (t, J = 8.7 Hz, 1H), 7.38 – 7.33 (m, 1H), 7.29 – 7.24 (m, 1H), 5.90 – 5.79 (m, 1H), 5.20 – 5.05 (m, 3H), 4.09 – 3.92 (m, 2H), 3.88 – 3.81 (m, 3H), 3.23 – 3.07 (m, 2H), 2.59 (b.s, 1H), 1.82 (dd, J = 14.7, 8.4 Hz, 3H), 1.27 (ddd, J = 22.2, 10.7, 5.2 Hz, 2H), 0.60 (b.s, 1H); **^{13}C NMR** (176 MHz, MeOD): δ = 163.18

(q, J = 35.0 Hz), 159.96, 158.55, 157.71, 148.02, 138.00, 137.03, 136.27, 135.56, 132.83, 131.10, 131.06, 130.60, 127.33, 121.57, 121.47, 120.75, 117.74, 117.59, 117.47, 115.45, 102.15, 69.15, 61.29, 56.95, 50.53, 49.97, 49.53, 38.30, 28.64, 23.99, 19.15.^{19}**F NMR** (377 MHz, MeOD): δ = -77.21, -117.55; **HRMS (ESI+)**: *m/z* = 455.1709 ([*M* + H]$^+$, calcd. for $C_{26}H_{27}O_2N_2{}^{37}ClF^+$: 455.1710) (Δ = 0.29 ppm) – No TFA visible; $[\boldsymbol{\alpha}]_{\mathbf{20}}^{\boldsymbol{D}}$= (c = 1.0, MeOH).

C3 substituted compound collection

(S)-(3-bromo-6-methoxyquinolin-4-yl)((2R,4S,5R)-5-vinylquinuclidin-2-yl)methanol-trifluoroacetate (14)

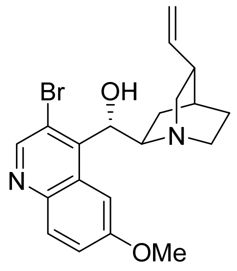

A flame dry 50 ml Schlenk-tube was charged with a solution of (+)-Quinidine (1.0 g, 3.08 mmol, 1.0 eq) in THF. Methyl lithium (1.93 mL, 3.08 mmol, 1.0 eq) was added dropwise at 0°C. After addition, a white opaque solution was formed and the mixture was stirred for 1 h at rt. BF_3*OEt_2 (0.42 mL, 3.39 mmol, 1.1 eq) was added at 0°C and stirred for 15 min. A solution of TMPMgCl*LiCl (3.40 mL, 3.39 mmol, 1.1 eq) was added dropwise at 0°C and stirred for 40 min at 0 °C. Then, 1,2-Dibromo-1,1,2,2-tetrachloroethane (0.80 g, 4.62 mmol, 1.5 eq) was added to the reaction and the mixture was stirred 15 h at rt. The reaction was quenched with 15 mL NH_4Cl/NH_3 solution (9:1) and extracted with DCM (3 x 20 mL). The organic phase was dry over Na_2SO_4 and purified by flash chromatography (Al_2O_3 III, isohexane / ethyl acetate = 1:1) to afford the desired compound as an oil (65%).

^{1}H NMR (500 MHz, MeOD): δ = 8.78 (s, 1H), 8.51 (s, br, 1H), 7.97 (d, J = 9.2 Hz, 1H), 7.50 (dd, J = 9.2, 2.8 Hz, 1H), 6.11 – 6.01 (m, 2H), 5.39-5.32 (m, 2H), 5.01 (m, 1H), 3.98 (s, 3H), 3.85 (dd, J = 16.7, 8.1 Hz, 1H), 3.54 – 3.48 (m, 1H), 2.89 (dd, J = 13.6, 10.1 Hz, 1H), 2.66 – 2.58 (m, 1H), 2.54 – 2.47 (m, 1H), 2.33 (dd, J = 9.6, 5.1 Hz, 1H), 2.19 – 2.11 (m, 1H), 1.80 – 1.69 (m, 2H), 1.65 – 1.54 (m, 2H); **HRMS (ESI+)**: *m/z* = 405.0994 ($[M + H]^+$, calcd. for $C_{20}H_{24}O_2N_2$ $^{81}Br^+$: 405.0995), (Δ = 0.35 ppm); $[\boldsymbol{\alpha}]_{20}^{D}$= + 82° (c = 1.0, MeOH). Data in accordance with the literature.[1]

(S)-(3-iodo-6-methoxyquinolin-4-yl)((2R,4S,5R)-5-vinylquinuclidin-2-yl)methanol-trifluoroacetate (15)

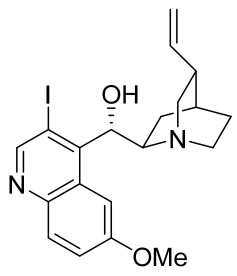

A flame dry 50 ml Schlenk-tube was charged with a solution of (+)-Quinidine (1.0 g, 3.08 mmol, 1.0 eq) in THF. Methyl lithium (1.93 mL, 3.08 mmol, 1.0 eq)) was added dropwise at 0°C. After addition, a white opaque solution was formed and the mixture was stirred for 1 h at rt. BF_3*OEt_2 (0.42 mL, 3.39 mmol, 1.1 eq) was added at 0°C and stirred for 15 min. A solution of TMPMgCl*LiCl (3.40 mL, 3.39 mmol, 1.1 eq) was added dropwise at 0°C and stirred for 40 min at 0 °C. Then, Iodine (1.17 g, 4.62 mmol, 1.5 eq) was added to the reaction and the mixture was stirred 15 h at rt. The reaction was quenched with 15 mL NH_4Cl/NH_3 solution (9:1) and extracted with DCM (3x20 mL). The organic phase was dry over Na_2SO_4 and purified by flash chromatography (Al_2O_3 III, isohexane / ethyl acetate = 1:1) to afford the desired compound as an oil (60%).

^{1}H NMR (500 MHz, MeOD): δ =8.80 (s, 1H), 8.10 (s, br, 1H), 7.85 (d, J = 9.2 Hz, 1H), 7.27 (dd, J = 9.0, 2.9 Hz, 1H), 5.82-5.73 (m, 1H), 5.51-5.44 (m, 1H), 5.00 (d, J = 1.4 Hz, 1H), 4.98 (dt, J = 7.2, 1.3 Hz, 1H), 3.89 (s, 3H), 3.62-3.47 (m, 1H), 2.95-2.84 (m, 1H), 2.70-2.60 (m, 2H), 2.31-2.25 (m, 1H), 1.90-1.83 (m, 2H), 1.77-1.69 (m, 2H), 1.55-1.48 (m, 2H); **HRMS (ESI+)**: *m/z* = 451.0868 ([*M* + H]$^+$, calcd. for $C_{20}H_{24}O_2N_2I^+$: 451.0877), (Δ = 1.95 ppm); $[\boldsymbol{\alpha}]_{\mathbf{20}}^{\boldsymbol{D}}$= + 39° (c = 1.0, MeOH). Data in accordance with the literature.[1]

(S)-(3-(4-fluorophenyl)-6-methoxyquinolin-4-yl)((2R,4S,5R)-5-vinylquinuclidin-2-yl)methanol- trifluoroacetate (16a)

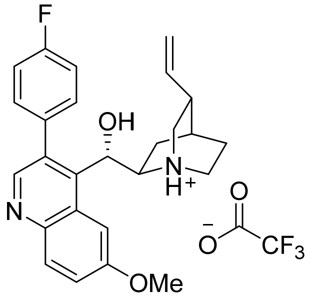

The compound was synthesized following **General Procedure V** starting with (R)-(3-bromo-6-methoxyquinolin-4-yl)((2S,4S,5R)-5-vinylquinuclidin-2-yl)methanol). Purified using preparative HPLC (ACN/H_2O + 0.1 % TFA) to afford the pure compound (22 mg, 63%).

^{1}H NMR (600 MHz, MeOD): δ = 8.65 (s, 1H), 8.23 (d, J = 2.6 Hz, 1H), 8.08 (d, J = 9.2 Hz, 1H), 7.61 (ddd, J = 12.0, 9.0, 4.0 Hz, 3H), 7.37 (dd, J = 16.5, 7.9 Hz, 2H), 5.91 (ddd, J = 17.1, 10.5, 6.5 Hz, 1H), 5.51 (d, J = 8.4 Hz, 1H), 5.18 (dd, J = 30.4, 13.8 Hz, 2H), 4.02 (s, J = 6.9 Hz, 3H), 3-38-3.31 (m, 1H), 3.00 – 2.94 (m, 1H), 2.91 (dd, J = 11.6, 5.1 Hz, 1H), 2.84 (ddd, J = 16.3, 9.5, 6.4 Hz, 1H), 2.72 (d, J = 3.5 Hz, 1H), 2.28 – 2.21 (m, 1H), 2.12 (d, J = 2.9 Hz, 1H), 2.04 (dd, J = 14.2, 7.3 Hz, 1H), 1.86 – 1.79 (m, 1H), 1.79 – 1.71 (m, 1H), 1.37 – 1.28 (m, 1H); **^{13}C NMR** (151 MHz, MeOD): δ = 161.85 (d, J = 38.2 Hz), 160.27, 148.84, 139.02, 136.04, 134.17, 133.17 (d, J = 8.3 Hz), 130.37, 129.15, 128.00, 124.58, 117.25, 117.00, 116.46 (d, J = 23.1 Hz), 106.02, 71.94, 61.67, 56.36, 55.24, 49.57, 44.06, 37.86, 36.01, 27.62, 27.30, 24.79, 24.51, 17.11; **^{19}F NMR** (565 MHz, MeOD): δ = -77.13, -113.94; **HRMS (ESI+)**: *m/z* = 419.2127 ($[M + H]^+$, calcd. for $C_{26}H_{28}O_2N_2F^+$: 419.2129) (Δ = 0.64 ppm) – No TFA visible; $[\alpha]_{20}^{D}$= + 24° (c = 1.0, MeOH).

(S)-(3-(4-chlorophenyl)-6-methoxyquinolin-4-yl)((2R,4S,5R)-5-vinylquinuclidin-2-yl)methanol- trifluoroacetate (16b)

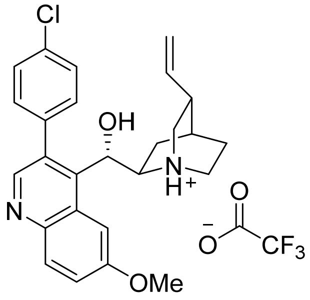

The compound was synthesized following **General Procedure V** starting with (R)-(3-bromo-6-methoxyquinolin-4-yl)((2S,4S,5R)-5-vinylquinuclidin-2-yl)methanol (25.0 mg, mmol, 1.0 eq). Purified using preparative HPLC (ACN/H_2O + 0.1 % TFA) to afford the pure compound (26 mg, 72%).

^{1}H NMR (700 MHz, MeOD): δ = 8.60 (s, 1H), 8.19 (d, J = 2.6 Hz, 1H), 8.08 (d, J = 9.2 Hz, 1H), 7.65 (d, J = 8.1 Hz, 2H), 7.60 – 7.54 (m, 3H), 5.92 (ddd, J = 17.1, 10.5, 6.5 Hz, 1H), 5.49 (d, J = 8.3 Hz, 1H), 5.23 (d, J = 10.5 Hz, 1H), 5.17 (d, J = 17.2 Hz, 1H), 4.02 (s, 3H), 3.35 (t, J = 6.8 Hz, 1H), 2.97 (ddd, J = 13.1, 4.8, 2.2 Hz, 1H), 2.94 – 2.88 (m, 2H), 2.74 (d, J = 3.6 Hz, 1H), 2.29 – 2.21 (m, 1H), 2.14 (d, J = 2.9 Hz, 1H), 2.06 (dd, J = 14.3, 7.2 Hz, 1H), 1.87 – 1.75 (m, 2H), 1.33 (dd, J = 19.8, 12.6 Hz, 1H); **^{13}C NMR** (176 MHz, MeOD): δ = 162.51 (d, J = 36.2 Hz), 159.97, 149.82, 145.19, 140.87, 138.98, 137.21, 136.20, 135.64, 132.62, 131.80, 130.59, 128.66, 123.78, 117.27, 105.83, 72.00, 61.73, 56.27, 55.34, 49.53, 44.11, 37.88, 31.12, 27.30, 24.78, 24.52; **HRMS (ESI+)**: *m/z* =437.1806 ([M + H]+, calcd. for $C_{26}H_{28}O_2N_2$ $^{37}Cl^{+}$: 437.1804) (Δ = 0.17 ppm) – No TFA visible; $[\boldsymbol{\alpha}]_{\mathbf{20}}^{\boldsymbol{D}}$= + 19° (c = 1.0, MeOH).

(S)-(3-(4-nitrophenyl)-6-methoxyquinolin-4-yl)((2R,4S,5R)-5-vinylquinuclidin-2-yl)methanol- trifluoroacetate (16c)

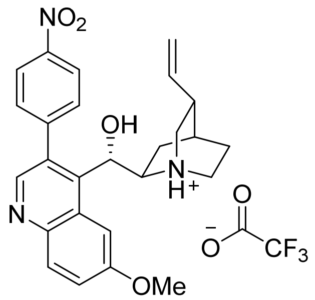

The compound was synthesized following **General Procedure V** starting with (R)-(3-bromo-6-methoxyquinolin-4-yl)((2S,4S,5R)-5-vinylquinuclidin-2-yl)methanol (25.0 mg, mmol, 1.0 eq). Purified using preparative HPLC (ACN/H_2O + 0.1 % TFA) to afford the pure compound (9 mg, 25%).

^{1}H NMR (700 MHz, MeOD): δ = 8.64 (d, J = 4.5 Hz, 2H), 8.25 (dd, J = 9.0, 2.1 Hz, 2H), 7.72 (dd, J = 9.6, 2.8 Hz, 1H), 7.44 (d, J = 2.6 Hz, 1H), 7.33 (d, J = 2.6 Hz, 1H), 6.08 – 5.98 (m, 1H), 5.97 (s, 1H), 5.18 (t, J = 14.0 Hz, 2H), 4.23 – 4.17 (m, 1H), 3.97 (s, 3H), 3.64 (t, J = 9.4 Hz, 1H), 3.51 – 3.42 (m, 2H), 3.33 – 3.27 (m, 1H), 3.11 (dd, J = 14.7, 7.2 Hz, 1H), 2.94 (dd, J = 14.5, 7.1 Hz, 1H), 2.68 (dd, J = 17.5, 8.7 Hz, 1H), 2.45 – 2.39 (m, 1H), 1.98 – 1.86 (m, 1H), 1.85 – 1.78 (m, 1H), 1.28 – 1.16 (m, 1H); **^{13}C NMR** (176 MHz, MeOD): δ = 159.40, 148.78, 148.49, 147.22, 146.42, 142.60, 142.27, 138.04, 132.75, 128.05, 124.00 (d, J = 11.6 Hz), 120.65, 117.72, 102.68, 80.43, 69.08, 61.37, 56.61, 50.53, 49.99, 49.53, 38.34, 35.36, 28.66, 24.02, 19.15; **HRMS (ESI+)**: *m/z* = 446.2071 ([*M* + H]$^+$, calcd. for $C_{26}H_{28}O_4N_3^+$: 446.2074) (Δ = 0.76 ppm) – No TFA visible; $[\alpha]_{20}^{D}$= - 18° (c = 1.0, MeOH).

(S)-(3-(4-trifluoromethylphenyl)-6-methoxyquinolin-4-yl)((2R,4S,5R)-5-vinylquinuclidin-2-yl)methanol- trifluoroacetate (16d)

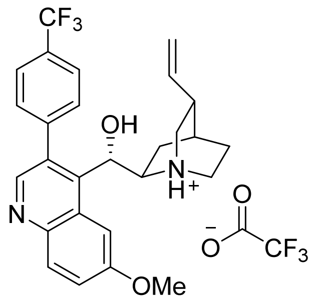

The compound was synthesized following **General Procedure V** starting with (R)-(3-bromo-6-methoxyquinolin-4-yl)((2S,4S,5R)-5-vinylquinuclidin-2-yl)methanol (25.0 mg, mmol, 1.0 eq). Purified using preparative HPLC (ACN/H_2O + 0.1 % TFA) to afford the pure compound (11 mg, 29%).

^{1}H NMR (600 MHz, MeOD): δ = 8.63 (s, 1H), 8.31 (s, 1H), 8.08 (d, J = 9.2 Hz, 1H), 7.94 (d, J = 7.6 Hz, 2H), 7.79 (s, 2H), 7.58 (dd, J = 9.2, 2.7 Hz, 1H), 5.75 – 5.67 (m, 1H), 5.52 (d, J = 6.9 Hz, 1H), 5.09 (dd, J = 10.7, 0.7 Hz, 1H), 4.74 (dd, J = 17.4, 0.7 Hz, 1H), 4.02 (s, 3H), 3.96 (q, J = 8.3 Hz, 1H), 3.15 – 3.06 (m, 3H), 2.91 – 2.85 (m, 1H), 2.68 – 2.61 (m, 1H), 2.20 – 2.13 (m, 1H), 2.06 (s, 1H), 2.02 – 1.87 (m, 3H); **^{13}C NMR** (151 MHz, MeOD): δ = 161.77 (d, J = 35.6 Hz), 160.09, 148.73, 144.38, 142.65, 142.04, 137.77, 135.41, 132.13, 131.59, 130.89, 129.92, 128.93, 128.19, 126.40, 124.59, 124.49, 116.91, 106.37, 72.82, 62.12, 57.62, 56.31, 50.89, 49.57, 37.14, 27.61, 24.20, 23.66; **^{19}F NMR** (565 MHz, MeOD): δ = -64.17, -77.22; **HRMS (ESI+)**: *m/z* = 469.2098 ([*M* + H]$^+$, calcd. for $C_{27}H_{28}O_2N_2F_3^+$: 469.2097) (Δ = 0.12 ppm) – No TFA visible; $[\alpha]_{20}^{D}$= - 57° (c = 1.0, MeOH).

(S)-(3-(4-*tert*-butyloxycarbonylaminophenyl)-6-methoxyquinolin-4-yl)((2R,4S,5R)-5-vinylquinuclidin-2-yl)methanol- trifluoroacetate (16e)

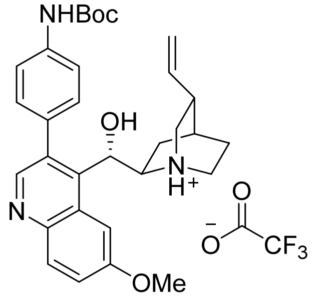

The title compound was synthesized following **General Procedure V** starting with (R)-(3-bromo-6-methoxyquinolin-4-yl)((2S,4S,5R)-5-vinylquinuclidin-2-yl)methanol (25.0 mg, mmol, 1.0 eq) and xx boronic acid (xx mg, xx mmols). Purified using preparative HPLC (ACN/H_2O + 0.1 % TFA) to afford the product as an amorphous solid (11 mg, 25%).

^{1}H NMR (500 MHz, MeOD): δ = 8.65 (s, 1H), 8.32 (d, J = 2.6 Hz, 1H), 8.07 (d, J = 9.3 Hz, 1H), 7.70 (d, J = 8.3 Hz, 2H), 7.56 (dd, J = 9.3, 2.7 Hz, 1H), 7.51 (d, J = 8.8 Hz, 2H), 5.77 – 5.68 (m, 1H), 5.64 (d, J = 7.8 Hz, 1H), 5.13 (d, J = 10.6 Hz, 1H), 4.78 (d, J = 17.5 Hz, 1H), 4.03 (s, 3H), 3.13 – 3.00 (m, 3H), 2.83 – 2.76 (m, 1H), 2.65 (d, J = 6.7 Hz, 1H), 2.17 – 1.98 (m, 4H), 1.93 (s, 1H), 1.58 (s, 9H), 1.39 – 1.30 (m, 1H); **^{13}C NMR** (126 MHz, MeOD): δ = 172.98, 159.96, 157.10, 155.11, 149.11, 147.06, 142.14, 141.77, 137.77, 136.77, 131.72, 131.53, 130.39, 129.17, 127.61, 124.18, 120.27, 117.00, 106.37, 101.40, 72.63, 62.09, 61.54, 56.26, 50.93, 49.63, 37.06, 28.65, 27.54, 24.73, 23.75, 20.86, 14.47; **HRMS (ESI+)**: *m/z* = 516.2853 ($[M + H]^+$, calcd. for $C_{31}H_{38}O_4N_3^+$: 516.2857) – No TFA visible., (Δ = 0.78 ppm); $[\alpha]_{20}^{D}$= + 72° (c = 1.0, MeOH).

(S)-(3-(3-fluorophenyl)-6-methoxyquinolin-4-yl)((2R,4S,5R)-5-vinylquinuclidin-2-yl)methanol- trifluoroacetate (16g)

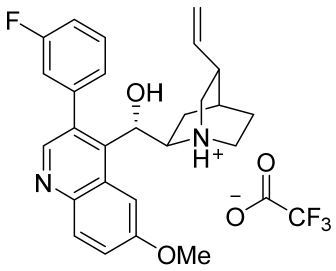

The compound was synthesized following **General Procedure V** starting with (R)-(3-bromo-6-methoxyquinolin-4-yl)((2S,4S,5R)-5-vinylquinuclidin-2-yl)methanol (25.0 mg, mmol, 1.0 eq). Purified using preparative HPLC (ACN/H_2O + 0.1 % TFA) to afford the pure compound (26 mg, 75%).

^{1}H NMR (600 MHz, MeOD): δ = 8.66 (s, 1H), 8.37 (s, J = 25.8 Hz, 1H), 8.08 (d, J = 9.3 Hz, 1H), 7.66 (dd, J = 14.0, 7.7 Hz, 1H), 7.60 (dd, J = 9.3, 2.7 Hz, 1H), 7.41 (d, J = 6.2 Hz, 2H), 7.34 (td, J = 8.5, 2.2 Hz, 1H), 5.79 – 5.72 (m, 1H), 5.61 (d, J = 7.0 Hz, 1H), 5.15 (d, J = 10.6 Hz, 1H), 4.83 (s, 1H), 4.03 (s, 3H), 3.99 – 3.92 (m, 1H), 3.16 – 3.07 (m, 3H), 2.98 – 2.91 (m, 1H), 2.70 – 2.61 (m, 1H), 2.23 – 2.16 (m, 1H), 2.07 (s, 1H), 2.03 – 1.88 (m, 3H); **^{13}C NMR** (151 MHz, MeOD): δ = 161.53 (q, J = 36.8 Hz), 160.21, 148.11, 143.26, 143.09, 140.28, 137.76, 135.65, 129.94, 129.91, 129.27, 129.21, 127.09, 126.42, 124.94, 118.44, 118.04, 117.04, 106.53, 72.77, 62.17, 56.61, 50.53, 49.57, 37.20, 27.67, 24.13, 23.66; **^{19}F NMR** (565 MHz, MeOD): δ = -77.10, -115.83. **HRMS (ESI+)**: *m/z* = 419.2126 ([*M* + H]$^+$, calcd. for $C_{26}H_{28}O_2N_2F^+$: 419.2129) (Δ = 0.92 ppm) – No TFA visible; $[\boldsymbol{\alpha}]^{\boldsymbol{D}}_{\mathbf{20}}$= ° (c = 1.0, MeOH).

(S)-(3-(3,4-difluorophenyl)-6-methoxyquinolin-4-yl)((2R,4S,5R)-5-vinylquinuclidin-2-yl)methanol- trifluoroacetate (16h)

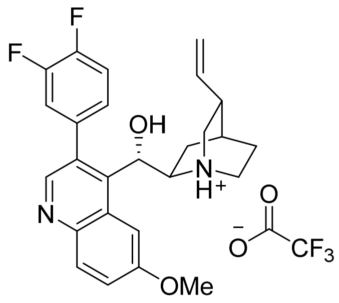

The compound was synthesized following **General Procedure V** starting with (R)-(3-bromo-6-methoxyquinolin-4-yl)((2S,4S,5R)-5-vinylquinuclidin-2-yl)methanol (25.0 mg, mmol, 1.0 eq). Purified using preparative HPLC (ACN/H_2O + 0.1 % TFA) to afford the pure compound (18 mg, 50%).

^{1}H NMR (600 MHz, MeOD): δ = 8.77 (d, J = 4.7 Hz, 1H), 7.88 (d, J = 4.6 Hz, 1H), 7.56 – 7.50 (m, 2H), 7.45 (d, J = 2.6 Hz, 1H), 7.43 – 7.34 (m, 2H), 6.18 – 6.09 (m, 2H), 5.29 (t, J = 13.4 Hz, 2H), 4.30 (ddd, J = 12.3, 8.4, 2.3 Hz, 1H), 4.07 (s, 3H), 3.74 (t, J = 9.4 Hz, 1H), 3.56 (q, J = 11.0 Hz, 2H), 3.44 – 3.35 (m, 1H), 2.78 (q, J = 8.5 Hz, 1H), 2.55 – 2.48 (m, 1H), 2.06 (s, 1H), 2.02 – 1.96 (m, 1H), 1.90 (dt, J = 11.4, 9.0 Hz, 1H), 1.36 – 1.24 (m, 1H); **^{13}C NMR** (151 MHz, MeOD): δ = 162.98 (d, J = 34.9 Hz), 159.72, 148.00, 147.56, 141.88, 138.04, 128.12, 127.98, 127.91, 124.19, 120.79, 120.67, 120.57, 118.10, 117,98, 117.72, 102.13, 69.02, 61.29, 56.80, 50.51, 49.93, 49.85, 49.57, 38.32, 28.67, 24.00, 19.08; **^{19}F NMR** (565 MHz, MeOD): δ = -76.84, -141.05, -141.93; **HRMS (ESI+)**: *m/z* = 437.2036 ($[M + H]^+$, calcd. for $C_{26}H_{27}O_2N_2F_2^+$: 437.2035) (Δ = 0.28 ppm) – No TFA visible; $[\alpha]_{20}^{D}$= + 91° (c = 1.0, MeOH).

(S)-(3-(3,5-difluorophenyl)-6-methoxyquinolin-4-yl)((2R,4S,5R)-5-vinylquinuclidin-2-yl)methanol- trifluoroacetate (16i)

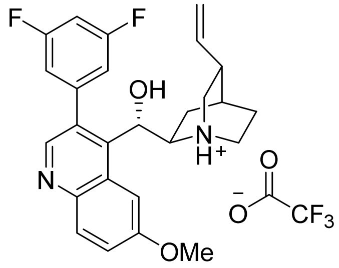

The compound was synthesized following **General Procedure V** starting with (R)-(3-bromo-6-methoxyquinolin-4-yl)((2S,4S,5R)-5-vinylquinuclidin-2-yl)methanol (25.0 mg, mmol, 1.0 eq). Purified using preparative HPLC (ACN/H_2O + 0.1 % TFA) to afford the pure compound (24 mg, 67%).

^{1}H NMR (500 MHz, MeOD): δ = 8.73 (d, J = 4.6 Hz, 1H), 7.81 (d, J = 4.5 Hz, 1H), 7.48 (d, J = 2.6 Hz, 1H), 7.37 (d, J = 2.6 Hz, 1H), 7.15 (tt, J = 10.2, 5.2 Hz, 2H), 7.01 (tt, J = 9.2, 2.3 Hz, 1H), 6.09 (ddd, J = 17.5, 10.4, 7.3 Hz, 1H), 6.02 (s, 1H), 5.33 – 5.19 (m, 2H), 4.26 (ddd, J = 12.4, 8.4, 2.3 Hz, 1H), 4.02 (s, 3H), 3.70 (t, J = 9.5 Hz, 1H), 3.53 (dd, J = 21.3, 10.6 Hz, 2H), 3.44 – 3.33 (m, 1H), 2.74 (dd, J = 17.4, 8.3 Hz, 1H), 2.48 (dd, J = 12.3, 10.6 Hz, 1H), 2.15 (dd, J = 15.1, 7.4 Hz, 1H), 2.05 – 1.91 (m, 2H), 1.91 – 1.83 (m, 1H); **^{13}C NMR** (151 MHz, MeOD): δ =165.03 (d, J = 11.5 Hz), 163.39 (d, J = 11.5 Hz), 159.83, 149.06, 145.43, 142.14, 141.27, 137.81, 134.37, 131.63, 128.66, 124.09, 118.16, 117.11, 106.33, 105.15 (t, J = 25.5 Hz), 101.24, 72.91, 62.39, 56.23, 50.87, 49.85, 37.31, 27.76, 23.66, 23.61; **^{19}F NMR** (377 MHz, MeOD): δ = -76.87, -112.64; **HRMS (ESI+)**: *m/z* = 437.2025 ([*M* + H]$^+$, calcd. for $C_{26}H_{27}O_2N_2F_2^+$: 437.2035) (Δ = 2.26 ppm) – No TFA visible; $[\alpha]_{20}^{D}$= + 73° (c = 1.0, MeOH).

(S)-(3-(3-chloro,4-fluorophenyl)-6-methoxyquinolin-4-yl)((2R,4S,5R)-5-vinylquinuclidin-2-yl)methanol- trifluoroacetate (16j)

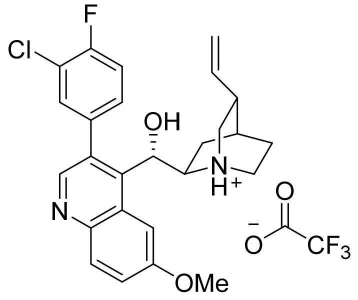

The compound was synthesized following **General Procedure V** starting with (R)-(3-bromo-6-methoxyquinolin-4-yl)((2S,4S,5R)-5-vinylquinuclidin-2-yl)methanol (25.0 mg, mmol, 1.0 eq). Purified using preparative HPLC (ACN/H_2O + 0.1 % TFA) to afford the pure compound (13 mg, 35%).

^{1}H NMR (500 MHz, MeOD): δ = 8.53 (s, 1H), 8.30 (s, 1H), 8.01 (d, J = 9.2 Hz, 1H), 7.73 (b.s, 1H), 7.52 – 7.43 (m, 2H), 7.20 – 7.07 (m, 1H), 5.87 – 5.73 (m, 1H), 5.50 (b.s, 1H), 5.16 (d, J = 10.6 Hz, 1H), 4.98 – 4.88 (m, 1H), 3.97 (s, 3H), 3.19 – 3.06 (m, 3H), 2.71 – 2.58 (m, 1H), 2.34 – 2.20 (m, 2H), 2.05 (s, 1H), 2.02 – 1.75 (m, 2H), 1.43 – 1.17 (m, 2H); **^{13}C NMR** (151 MHz, MeOD): δ = 159.80, 149.49, 145.40, 141.42, 137.84, 137.82, 134.37, 133.18, 131.64, 131.45, 131.40, 128.72, 123.96, 122.72, 117.04, 106.30, 73.00, 62.35, 56.22, 50.87, 49.85, 49.57, 37.27, 27.73, 23.60, 1.79; **^{19}F NMR** (377 MHz, MeOD): δ = -76.74, -117.93; **HRMS (ESI+)**: *m/z* = 455.1705 ([*M* + H]$^+$, calcd. for $C_{26}H_{27}O_2N_2{}^{37}ClF^+$: 455.1710) – No TFA visible., (Δ = 1.16 ppm); $[\alpha]_{20}^{D}$= - 2° (c = 1.0, MeOH).

(S)-(3-(3,4-dimethoxyphenyl)-6-methoxyquinolin-4-yl)((2R,4S,5R)-5-vinylquinuclidin-2-yl)methanol- trifluoroacetate (16k)

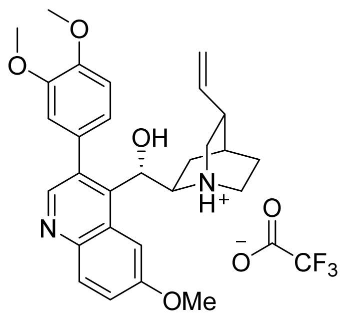

The compound was synthesized following **General Procedure V** starting with (R)-(3-bromo-6-methoxyquinolin-4-yl)((2S,4S,5R)-5-vinylquinuclidin-2-yl)methanol (25.0 mg, mmol, 1.0 eq). Purified using preparative HPLC (ACN/H_2O + 0.1 % TFA) to afford the pure compound (15 mg, 40%).

^{1}H NMR (700 MHz, MeOD): δ = 8.81 (d, J = 5.2 Hz, 1H), 8.14 (d, J = 5.1 Hz, 1H), 7.62 (dt, J = 8.9, 4.6 Hz, 2H), 7.22 – 7.11 (m, 3H), 6.39 (s, 1H), 6.15 (ddd, J = 17.5, 10.4, 7.3 Hz, 1H), 5.31 (dd, J = 19.6, 13.8 Hz, 2H), 4.30 (ddd, J = 12.2, 8.4, 2.2 Hz, 1H), 4.13 (d, J = 17.7 Hz, 3H), 3.97 – 3.93 (m, 3H), 3.90 (s, 3H), 3.78 (dd, J = 16.5, 6.9 Hz, 1H), 3.59 (dd, J = 21.2, 10.0 Hz, 2H), 3.44 – 3.36 (m, 1H), 2.81 (dd, J = 17.4, 8.5 Hz, 1H), 2.59 – 2.51 (m, 1H), 2.07 (d, J = 19.0 Hz, 1H), 2.06 – 1.97 (m, 1H), 1.91 (dt, J = 11.5, 9.3 Hz, 1H), 1.37 – 1.28 (m, 1H); **^{13}C NMR** (176 MHz, MeOD): δ = 163.24 (d, J = 35.2 Hz), 160.90, 151.39, 150.77, 144.64, 138.05, 130.14, 129.01, 127.01, 123.88, 120.88, 117.75, 115.00, 113.33, 106.52, 101.94, 68.94, 61.03, 57.36, 56.92, 56.60, 56.31, 50.51, 49.80, 49.53, 38.29, 28.71, 24.49, 23.99, 18.93; **HRMS (ESI+)**: *m/z* = 461.2439 ([*M* + H]$^+$, calcd. for $C_{28}H_{33}O_4N_2^+$: 461.2435) (Δ = 0.91 ppm) – No TFA visible; $[\boldsymbol{\alpha}]_{\mathbf{20}}^{\boldsymbol{D}}$= - 69° (c = 1.0, MeOH).

(S)-(3-(3,5-dimethoxyphenyl)-6-methoxyquinolin-4-yl)((2R,4S,5R)-5-vinylquinuclidin-2-yl)methanol- trifluoroacetate (16l)

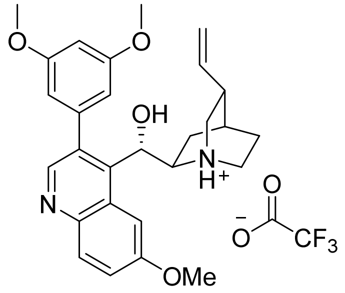

The compound was synthesized following **General Procedure V** starting with (R)-(3-bromo-6-methoxyquinolin-4-yl)((2S,4S,5R)-5-vinylquinuclidin-2-yl)methanol (25.0 mg, mmol, 1.0 eq). Purified using preparative HPLC (ACN/H_2O + 0.1 % TFA) to afford the pure compound (9 mg, 24%).

^{1}H NMR (700 MHz, MeOD): δ = 8.72 (s, 1H), 8.40 (d, J = 2.7 Hz, 1H), 8.11 (d, J = 9.2 Hz, 1H), 7.64 (dd, J = 9.3, 2.7 Hz, 1H), 6.71 (t, J = 2.2 Hz, 2H), 6.52 (d, J = 2.3 Hz, 1H), 5.77 (ddd, J = 17.4, 10.7, 5.6 Hz, 1H), 5.72 (d, J = 7.6 Hz, 1H), 5.17 (ddd, J = 10.7, 1.7, 0.7 Hz, 1H), 4.85 – 4.81 (m, 1H), 4.04 (s, 3H), 3.88 (s, 6H), 3.72 (s, 1H), 3.14 – 3.07 (m, 2H), 2.97 (ddd, J = 13.0, 8.1, 2.4 Hz, 1H), 2.67 (m, 1H), 2.16 (ddd, J = 13.2, 6.3, 4.3 Hz, 1H), 2.08 (m, 1H), 2.05 – 1.98 (m, 2H), 1.93 (m, 1H); **^{13}C NMR** (176 MHz, MeOD): δ =162.70 (q, J = 34.6 Hz), 161.83, 147.24, 144.54, 141.47, 139.37, 137.90, 137.14, 129.63, 128.75, 125.30, 121.09, 117.74, 116.87, 109.17, 106.69, 102.50, 101.51, 72.55, 62.20, 56.40, 56.22, 56.01, 55.64, 55.58, 50.97, 48.48, 37.10, 27.62, 24.43, 23.71, 18.83; **HRMS (ESI+)**: *m/z* = 461.2423 ([*M* + H]$^+$, calcd. for $C_{28}H_{33}O_4N_2^+$: 461.2435) – No TFA visible., (Δ = 2.48 ppm); $[\alpha]^D_{20}$= - 17° (c = 1.0, MeOH).

(S)-((1S,2S,4S,5R)-5-ethylquinuclidin-2-yl)(2-(4-fluorophenyl)-6-methoxyquinolin-4-yl)methanol- trifluoroacetate (17a) *and* (S)-((1S,2S,4S,5R)-5-ethylquinuclidin-2-yl)(5-(4-fluorophenyl)-6-methoxyquinolin-4-yl)methanol- trifluoroacetate (18a)

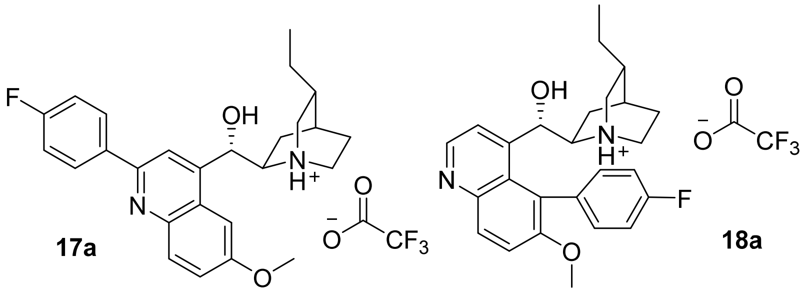

The compound was synthesized following **General procedure I** starting with dihydroquinidine **27** (40 mg, 120 μmol). Crude was purified using preparative HPLC (ACN/H_2O + 0.1 % TFA) to afford the pure compounds **23** (31 mg, 48%) and **25** (6 mg, 10%)

17a: **^{1}H NMR** (400 MHz, MeOD): δ = 8.23 – 8.15 (m, 3H), 8.12 (d, J = 9.3 Hz, 1H), 7.53 (dd, J = 9.3, 2.6 Hz, 1H), 7.36 (d, J = 2.6 Hz, 1H), 7.34 – 7.29 (m, 2H), 6.04 (s, 1H), 4.06 – 3.99 (m, 3H), 3.72 (t, J = 9.4 Hz, 1H), 3.55 (t, J = 11.2 Hz, 2H), 3.42 – 3.34 (m, 1H), 2.56 – 2.48 (m, 1H), 2.05 (s, 1H), 1.93 (dt, J = 12.7, 7.5 Hz, 3H), 1.77 – 1.60 (m, 2H), 1.38 – 1.29 (m, 2H), 1.00 (t, J = 7.4 Hz, 3H); **^{13}C NMR** (101 MHz, MeOD): δ = 166.56, 160.18, 154.96, 147.53, 144.91, 136.54, 132.12, 130.68 (d, J = 8.6 Hz), 126.22, 123.72, 117.97, 116.92, 116.71, 102.00, 69.18, 61.50, 56.48, 51.70, 50.61, 49.85, 36.23, 26.33, 25.38, 24.57, 18.92, 11.78; **^{19}F NMR** (377 MHz, MeOD): δ = -76.98, -114.25; **HRMS (ESI+)**: *m/z* = 421.2274 ([*M* + H]$^+$, calcd. for $C_{26}H_{30}O_2N_2F^+$: 421.2286) (Δ = 2.77 ppm) – No TFA visible; $[\alpha]_{20}^{D}$= + 79° (c = 1.0, MeOH).

18a: **^{1}H NMR** (400 MHz, MeOD): δ = 8.87 (d, J = 4.9 Hz, 1H), 8.26 (d, J = 9.4 Hz, 1H), 8.14 (d, J = 4.8 Hz, 1H), 7.88 (dd, J = 9.4, 4.8 Hz, 1H), 7.76 – 7.70 (m, 1H), 7.37 (td, J = 8.6, 2.7 Hz, 1H), 7.26 (td, J = 8.6, 2.7 Hz, 1H), 7.20 – 7.14 (m, 1H), 5.08 (d, J = 3.6 Hz, 1H), 3.86 (s, 3H), 3.64 – 3.55 (m, 1H), 3.16 (ddd, J = 21.3, 17.3, 10.0 Hz, 2H), 1.88 – 1.71 (m, 5H), 1.43 (tt, J = 13.8, 6.8 Hz, 2H), 1.31 (d, J = 6.5 Hz, 2H), 0.89 (t, J = 7.4 Hz, 3H), 0.72 – 0.62 (m, 1H); **^{13}C NMR** (101 MHz, MeOD): δ = 163.12, 158.29, 152.59, 149.93, 146.82, 134.38 (dd, J = 3.6, 1.9 Hz), 134.07 (d, J = 8.0 Hz), 133.43 (d, J = 7.9 Hz), 130.70, 126.78, 123.97, 122.58, 119.03, 116.95 (dd, J = 38.8, 21.7 Hz), 69.00, 60.10, 57.20, 52.82, 50.65, 49.71, 35.92, 26.41, 25.07, 23.88, 19.69, 11.61; **^{19}F NMR** (377 MHz, MeOD): δ = -76.98, -114.05; **HRMS (ESI+)**: *m/z* = 421.2274 ([*M* + H]$^+$, calcd. for $C_{26}H_{30}O_2N_2F^+$: 421.2286) (Δ = 2.77 ppm) – No TFA visible; $[\alpha]_{20}^{D}$= + 79° (c = 1.0, MeOH).

(S)-((1S,2S,4S,5R)-5-ethylquinuclidin-2-yl)(2-(4-chlorophenyl)-6-methoxyquinolin-4-yl)methanol- trifluoroacetate (17b) ***and*** **(S)-((1S,2S,4S,5R)-5-ethylquinuclidin-2-yl)(5-(4-chlorophenyl)-6-methoxyquinolin-4-yl)methanol- trifluoroacetate (18b)**

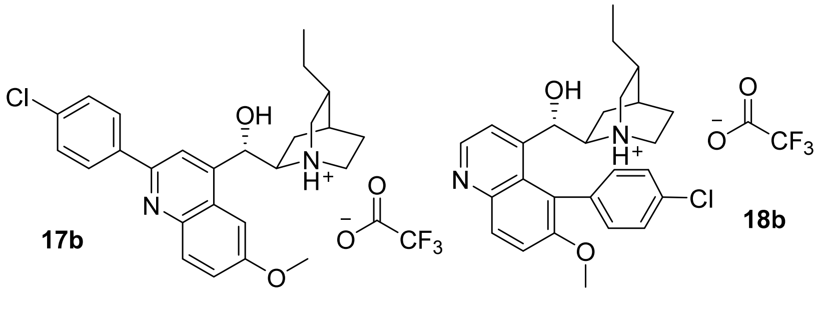

The compound was synthesized following **General procedure I** starting with dihydroquinidine (40 mg, 120 µmol). Crude was purified using preparative HPLC (ACN/H_2O + 0.1 % TFA) to afford the pure compounds **17b** (28 mg, 42%) and **18b** (4 mg, 6%) as amorphous solids.

17b: ^{1}**H NMR** (500 MHz, MeOD): δ = 8.28 (s, 1H), 8.15 (d, J = 9.3 Hz, 1H), 8.09 (d, J = 8.6 Hz, 2H), 7.63 – 7.59 (m, 3H), 7.51 (d, J = 2.4 Hz, 1H), 6.24 (s, 1H), 4.05 (s, 3H), 3.99 – 3.91 (m, 1H), 3.68 (t, J = 9.4 Hz, 1H), 3.50 (t, J = 11.2 Hz, 2H), 3.32 (d, J = 9.2 Hz, 1H), 2.52 – 2.44 (m, 1H), 1.99 (s, 1H), 1.95 – 1.81 (m, 3H), 1.65 (qd, J = 13.7, 7.3 Hz, 2H), 1.31 – 1.24 (m, 1H), 0.96 (t, J = 7.4 Hz, 3H); ^{13}**C NMR** (126 MHz, MeOD): δ = 160.92, 153.38, 146.12, 130.51, 130.37, 129.22, 129.07, 126.80, 125.77, 122.10, 118.72, 102.42, 68.79, 61.07, 57.06, 53.59, 51.33, 50.61, 50.37, 36.03, 28.40, 26.19, 25.22, 24.38, 18.53, 11.63; **HRMS (ESI+)**: *m/z* = 439.1967 ($[M + H]^+$, calcd. for $C_{26}H_{30}O_2N_2{}^{37}Cl^+$:439.1961) (Δ = 1.51 ppm) – No TFA visible; $[\boldsymbol{\alpha}]_{\mathbf{20}}^{\boldsymbol{D}}$= + 112° (c = 1.0, MeOH).

18b: ^{1}**H NMR** (500 MHz, MeOD): δ = 8.92 (d, J = 5.1 Hz, 1H), 8.26 (dd, J = 18.0, 7.2 Hz, 2H), 7.96 (d, J = 9.4 Hz, 1H), 7.68 (dd, J = 8.2, 2.0 Hz, 1H), 7.61 (dd, J = 8.2, 2.2 Hz, 1H), 7.51 (dd, J = 8.2, 2.2 Hz, 1H), 7.11 (d, J = 8.2 Hz, 1H), 5.13 (d, J = 3.7 Hz, 1H), 3.85 (s, 2H), 3.57 – 3.49 (m, 1H), 3.23 – 3.01 (m, 3H), 1.84 – 1.65 (m, 5H), 1.39 (s, 2H), 1.25 (s, 2H), 0.85 (t, J = 7.4 Hz, 3H), 0.70 – 0.60 (m, 1H); **HRMS (ESI+)**: *m/z* = 439.1967 ($[M + H]^+$, calcd. for $C_{26}H_{30}O_2N_2\,Cl^+$:) (Δ = 2.77 ppm) – No TFA visible; $[\boldsymbol{\alpha}]_{\mathbf{20}}^{\boldsymbol{D}}$= ° (c = 1.0, MeOH).

(6-methoxyquinolin-4-yl)((1S,2S,4S,5R)-5-vinylquinuclidin-2-yl)methanone (26)

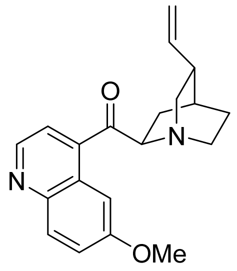

(+)-Quinidine (5.0 g, 15.4 mmol, 1.0 eq) was dissolved in anhydrous toluene (100 ml) and stirred for 5 min under an argon atmosphere. Then, benzophenone (5.59 g, 30.7 mmol, 2.0 eq) and potassium tert-butoxide (4.34 g, 38.7 mmol, 2.5 eq) were added to the solution. The mixture was stirred for 16 h at reflux. The resulting orange viscous mixture was cooled to 0°C and a 3M HCl (40 ml) was added slowly in order to keep the temperature below 30°C. The organic phase was separated, washed with 3 M HCl solution (3x30 ml). The combined aqueous extracts were cooled to 0°C and basified by dropwise addition of conc. ammonia solution (10 ml). The aqueous phase was saturated with brine (20 mL) and extracted with DCM (5x40 ml). The organic phases were recombined, dried over $MgSO_4$ and concentrated under vaccuo. The crude was purified by flash chromatography (EtOAc/acetone 9:1 -> 6:4, 25min) to afford the desired compound (2.8 g, 60%).

^{1}H NMR (700 MHz, MeOD): δ = 8.73 (t, J = 5.0 Hz, 1H), 7.93 (dd, J = 9.2, 2.3 Hz, 1H), 7.71 (t, J = 5.6 Hz, 1H), 7.56 (d, J = 2.6 Hz, 1H), 7.39 (dd, J = 9.2, 2.5 Hz, 1H), 5.88 – 5.80 (m, 1H), 5.09 – 4.96 (m, 2H), 3.86 (t, J = 7.3 Hz, 3H), 3.09 – 2.99 (m, 1H), 2.87 – 2.74 (m, 3H), 2.33 – 2.22 (m, 1H), 2.00 (t, J = 11.6 Hz, 1H), 1.86 (s, 1H), 1.76 – 1.71 (m, 1H), 1.69 (dt, J = 10.4, 5.2 Hz, 1H), 1.64 – 1.46 (m, 2H); **HRMS (ESI+)**: *m/z* = 323.1751 ($[M + H]^+$, calcd. for $C_{20}H_{23}O_2N_2^+$: 323.1754) (Δ = 0.99 ppm) – No TFA visible; $[\boldsymbol{\alpha}]_{\mathbf{20}}^{\boldsymbol{D}}$= - 99° (c = 1.0, MeOH). Data in accordance with the literature.[2]

(2-(4-fluorophenyl)-6-methoxyquinolin-4-yl)((1S,2S,4S,5R)-5-vinylquinuclidin-2-yl)methanone- trifluoroacetate (20)

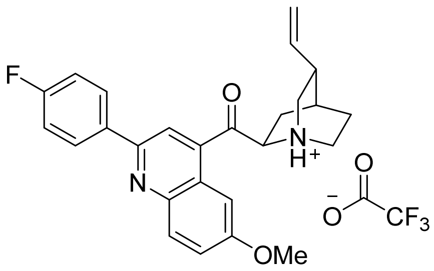

20 was synthesized following General Procedure I starting with compound **26** (40 mg, 120 µmol). Crude was purified using preparative HPLC (ACN/H_2O + 0.1 % TFA) to afford the pure compound (25 mg, 40%).

^{1}H NMR (400 MHz, MeOD): δ = 8.36 (s, 1H), 8.29 (m, 2H), 8.13 (d, J = 9.2 Hz, 1H), 7.87 – 7.79 (m, 1H), 7.55 (dt, J = 9.3, 2.2 Hz, 1H), 7.31 (m, 2H), 5.87 (ddd, J = 17.0, 10.6, 6.1 Hz, 1H), 5.42 – 5.33 (m, 1H), 5.26 – 5.19 (m, 2H), 4.11 (q, J = 7.1 Hz, 2H), 3.99 (s, 3H), 3.82 – 3.72 (m, 1H), 3.71 – 3.58 (m, 1H), 3.59 – 3.45 (m, 2H), 3.00 – 2.85 (m, 1H), 2.23 (m, 2H), 1.91 (m, 1H); **^{13}C NMR** (101 MHz, MeOD): δ = 203.46, 160.52, 147.94, 145.68, 142.71, 142.35, 141.12, 131.31, 126.99, 123.82, 123.64, 121.69, 121.19, 115.18, 103.93, 103.75, 55.96, 55.83, 49.98, 43.83, 40.61, 40.21, 28.75, 27.89, 27.13, 23.44; **^{19}F NMR** (377 MHz, MeOD): δ = -76.92, -114.02; **HRMS (ESI+)**: *m/z* = 417.1963 ([*M* + H]$^+$, calcd. for $C_{26}H_{26}O_2N_2F^+$: 417.1973) (Δ = 2.33 ppm) – No TFA visible; $[\alpha]_{20}^{D}$= + 11° (c = 1.0, MeOH).

(*1S,2R,4S,5S*)-2-[(*S*)-[2-(4-fluorophenyl)-6-methoxyquinolin-4-yl](hydroxy)methyl]-5-[(1*E*)-2-phenylethenyl]-1-azabicyclo[2.2.2]octan-1-ium trifluoroacetate (19a)

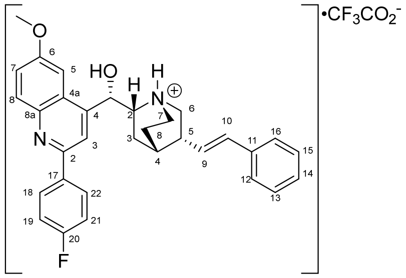

Prepared according the general procedure for Heck couplings using Bromobenzene as aryl bromide. Mass directed, preparative HPLC gave the desired product (14.3 mg, 23.5 µmol, 16%) as pale-yellow solid.

^{1}H-NMR (500 MHz, CD_3OD, OH, NH^+ not observed) δ 8.32 (1H, s, quinoline 3-H), 8.20-8.13 (3H, m, 18-H. 22-H and quinoline 8-H), 7.63-7.55 (2H, m, quinoline 5-H and quinoline 7-H), 7.48 (2H, d, *J* 7.3, 12-H and 16-H), 7.39-7.30 (4H, m, 13-H, 15-H, 19-H and 21-H), 7.26 (1H, t, *J* 7.3, 14-H), 6.64 (1H, d, *J* 16.0, 10-H), 6.56 (1H,

dd, *J* 16.0, 7.5, 9-H), 6.42 (1H, s, C*H*OH), 4.37-4.30 (1H, m, quinuclidine 6-H_A), 4.08 (3H, s, OCH_3), 3.79 (1H, t, *J* 9.4, quinuclidine 2-H), 3.66-3.53 (2H, m, quinuclidine 6-H_B and quinuclidine 7-H_A), 3.44-3.35 (1H, m, quinuclidine 7-H_B), 2.99-2.91 (1H, m, quinuclidine 5-H), 2.73-2.66 (1H, m, quinuclidine 3-H_A), 2.13 (1H, app. s, quinuclidine 4-H), 2.08-1.99 (1H, m, quinuclidine 8-H_A), 1.97-1.89 (1H, m, quinuclidine 8-H_B), 1.44-1.36 (1H, m, quinuclidine 8-H_B). **^{13}C NMR** (151 MHz, CD_3OD, *C*$F_3CO_2^-$ and CF_3*C*O_2^- not observed) 162.7 (Ar-C_q), 162.5 (Ar-C_q), 160.6 (Ar-C_q), 154.4 (Ar-C_q), 138.3 (Ar-C_q), 133.8 (10-C), 131.1 (d, J_{CF} 8.1, 18-C and 22-C)), 129.7 (13-C and 15-C), 128.8 (9-C), 127.5 (14-C), 126.6 (12-C and 16-C), 119.0 (quinoline 7-C), 118.5 (Ar-C_q) (quinoline 3-C), 117.1 (Ar-C_q), 117.0 (d, J_{CF} 22.2, 19-C and 21-C), 116.9 (Ar-C_q), 102.5 (quinoline 5-C), 69.0 (CHOH), 61.5 (quinuclidine 2-C), 57.3 (OCH_3), 49.8 (quinuclidine 7-C), 49.6 (quinuclidine 6-C), 38.1 (quinuclidine 5-C), 29.2 (quinuclidine 4-C), 24.1 (quinuclidine 8-C), 19.1 (quinuclidine 3-C). **HRMS** (ESI): $C_{32}H_{31}FO_2N_2$ $[M+H]^+$; calculated: 495.2442, found: 495.2441. $[\alpha]^D_{20}$ = +108 (c. 0.1, MeOH).

(*1S*,*2R*,*4S*,*5S*)-2-[(*S*)-[2-(4-Fluorophenyl)-6-methoxyquinolin-4-yl](hydroxy)methyl]-5-[(*1E*)-2-(4-fluorophenyl)ethenyl]-1-azabicyclo[2.2.2]octan-1-ium trifluoroacetate (19b)

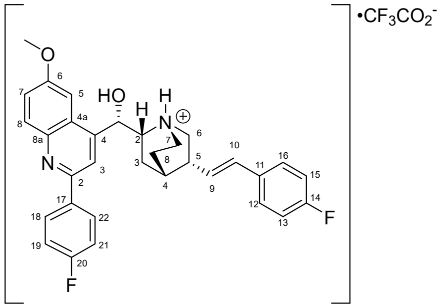

Prepared according the general procedure for Heck couplings using 4-fluoro-bromobenzene as aryl bromide. Mass directed, preparative HPLC gave the desired product (6,0 mg, 9,58 μmol, 8%) as pale-yellow solid.

^{1}H-NMR (500 MHz, CD_3OD, OH, NH^+ not observed) δ 8.29 (1H, s, quinoline 3-H), 8.22-8.16 (2H, m, 18-H and 22-H), 8.14 (1H, d, *J* 9.3, quinoline 8-H), 7.57 (1H, d, *J* 9.3, quinoline 7-H), 7.52-7.48 (2H, m, 12-H and 16-H), 7.48-7.40 (1H, m, quinoline 5-H), 7.38-7.30 (2H, m, 19-H and 21-H), 7.13-7.06 (2H, m, 13-H and 15-H), 6.62 (1H, d, *J* 16.0, 10-H), 6.49 (1H, dd, *J* 16.0, 7.6, 9-H), 6.18 (1H, s, C*H*OH), 4.35-4.28 (1H, m, quinuclidine 6-H_A), 4.04 (3H, s, OCH_3), 3.80 (1H, app. s, quinuclidine 2-H), 3.67-3.56 (2H, m, quinuclidine 6-H_B and quinuclidine 7-H_A), 3.46-3.39 (1H, m, quinuclidine 7-H_B), 3.34-3.33 (1H, m), 2.98-2.90 (1H, m, quinuclidine 5-H), 2.71-2.64 (1H, m, quinuclidine 3-H_A), 2.16-2.12 (1H, m, quinuclidine 4-H), 2.07-2.00 (1H, m, quinuclidine 8-H_A), 1.97-1.89 (1H, m, quinuclidine 8-H_B), 1.47-1.37 (1H, m, quinuclidine 3-H_B)

^{13}**C NMR** (151 MHz, $CF_3CO_2^-$ not observed) δ 166.3 (Ar-C_q), 164.7 (Ar-C_q), 163.0 (Ar-C_q), 162.2 (q, J_{CF} 36.1, $CF_3CO_2^-$), 160.3 (Ar-C_q), 154.8 (Ar-C_q), 136.0 (Ar-C_q), 134.7 (Ar-C_q), 132.6 (10-C), 131.6 (Ar-C_q), 130.8 (d, J_{CF} 8.5, 18-H and 22-H), 129.2 (d, J_{CF} 8.1, 12-H and 16-H), 129.1 (9-C), 126.4 (Ar-C_q), 124.1 (quinoline 7-C), 118.3 (quinoline 3-H), 116.9 (d, J_{CF} 22.0, C-19, C-21), 116.4 (d, J_{CF} 21.8, C-13 and C-15), 102.2 (quinoline 5-C), 69.3 (CHOH), 61.6 (quinuclidine 2-C), 56.6 (OCH_3), 50.5 (quinuclidine 6-C), 49.6 (quinuclidine 7-C), 38.0 (quinuclidine 5-C), 29.1 (quinuclidine 4-C), 24.1 (quinuclidine 8-C), 19.3 (quinuclidine 3-C).

HRMS (ESI): $C_{32}H_{30}F_2O_2N_2$ $[M+H]^+$; calculated: 513.2348 found: 513.2345. $[\boldsymbol{\alpha}]_{\mathbf{20}}^{\boldsymbol{D}}$ = +122 (c. 0.1, MeOH).

(*S*)-[2-(4-fluorophenyl)-6-methoxyquinolin-4-yl][(1*S*,2*R*,4*S*,5*S*)-5-[(1*E*)-2-(3-methoxyphenyl)ethenyl]-1-azabicyclo[2.2.2]octan-2-yl]methanol (19c)

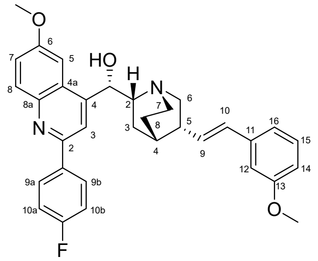

Prepared according the general procedure for Heck couplings using 3-methoxybromobenzene as aryl bromide. Flash column chromatography gave the desired product (5.2 mg, 9.91 µmol, 8%) as light brown solid.

^{1}**H-NMR** (500 MHz, CD_3OD, O*H* not observed) δ 8.22 (1H, s, quinoline 3-H), 8.20-8.15 (2H, m, 9-H_a and 9H_b), 8.09 (1H, d, *J* 9.2, quinoline 8-H), 7.49 (1H, dd, *J* 9.2, 2.7, quinoline 7-H), 7.39 (1H, app. S, quinoline 5-H), 7.30-7.26 (3H, m, quinoline e10-H_a,10-H_b and 15-H), 7.06 (1H, d, *J* 7.8, 16-H), 7.01-6.99 (1H, m, 12-H), 6.86 (1H, dd, *J* 7.8, 2.4, 14-H), 6.62-6.48 (2H, m, 9-H and 10-H), 6.07 (1H, app. s, C*H*OH), 4.33-4.27 (1H, m, quinuclidine 6-H_A), 4.02 (3H, s, OCH_3), 3.83 (3H, s, OCH_3), 3.82 – 3.77 (1H, m, quinuclidine 2-H), 3.64-3.53 (2H, m, quinuclidine 6-H_B and quinuclidine 7-H_A), 3.44-3.35 (1H, m, quinuclidine 7-H_B), 2.96-2.88 (1H, m, quinuclidine 5-H), 2.67-2.61 (1H, m, quinuclidine 3-H_A), 2.13 (1H, app. s, quinuclidine 4-H), 2.07-1.89 (2H, m, quinuclidine 8-H_A and quinuclidine 8-H_B), 1.46-1.36 (1H, m, quinuclidine 3-H_B). ^{13}**C NMR** (151 MHz, CD_3OD) δ 166.0 (Ar-C_q), 164.4 (Ar-C_q), 161.5 (Ar-C_q), 160.0 (Ar-C_q), 155.2 (Ar-C_q), 145.5 (Ar-C_q), 139.7 (Ar-C_q), 137.0 (Ar-C_q), 133.6 (9-C or 10-C), 132.6 (quinoline 8-C), 130.7 (15-C) 130.5 (d, J_{CF} 8.5, 10a-C and 10b-C), 129.6 (9-C or 10-C), 126.2 (Ar-C_q), 123.4 (quinoline 7-C), 119.9 (16-C), 117.9 (quinoline 3-C), 116.71 (d, J_{CF} 21.9, 9a-C and 9b-C), 114.1 (14-C), 113.1 (12-C), 102.1 (quinoline 5-C), 69.6 (*C*HOH), 61.7 (quinuclidine 2-C), 56.5 (OCH_3), 55.7 (OCH_3), 50.6

(quinuclidine 6-C), 50.4 (quinuclidine 7-C), 38.1 (quinuclidine 5-C), 29.1 (quinuclidine 4-C), 24.2 (quinuclidine 8-C), 19.6 (quinuclidine 3-C). **HRMS** (ESI): $C_{33}H_{33}FO_3N_2$ $[M+H]^+$;calculated: 525.2548, found: 525.2546. $[\boldsymbol{\alpha}]_{\mathbf{20}}^{\boldsymbol{D}}$ +125 (c. 0.1, MeOH).

4-[(*S*)-[(*1S*,*2R*,*4S*,*5R*)-5-ethenyl-1-azabicyclo[2.2.2]octan-2-yl](methoxy)methyl]-6-methoxyquinoline (28)

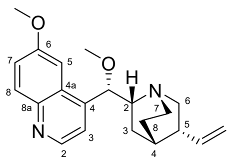

Quinidine (500 mg, 1.54 mmol, 1.0 eq.) was added to a heat and vacuum dried Schlenk flask, degassed with Argon for 10 min and then dissolved in anhydrous DMF (0.3 M, 5 mL). NaH (60% in mineral oil; 184.9 mg, 4.62 mmol, 3.0 eq.) was added portionwise and the mixture was stirred at rt for 1h. Then MeI (95 µL, 1.54 mmol, 1.0 eq.) was added dropwise and the reaction stirred at rt for 16 h, to reach full conversion of the starting material. The reaction was quenched with aqueous, saturated brine (10 mL) followed by extraction with EtOAc (5 x 25 mL). The combined organic layers were dried over Mg_2SO_4, filtrated and concentrated in vacuo. Flash column chromatography on pre-equilibrated silica (DCM:EtOH:NH_4OH 50:8:1) eluting with 0-10% MeOH in DCM gave the title compound (216 mg, 0.64 mmol, 41%) as yellow oil. **^{1}H NMR** (500 MHz, CD_3OD) δ 8.69 (1H, d, *J* 4.6, quinoline 2-H), 7.97 (1H, d, *J* 9.3, quinoline 8-H), 7.58 (1H, d, *J* 4.6, quinoline 3-H), 7.45 (1H, dd, *J* 9.3, 2.5, quinoline 7-H), 7.41 (1H, d, *J* 2.5, quinoline 5-H), 6.17-6.07 (1H, m, C*H*CH_2), 5.23 (1H, d, *J* 2.3, C*H*-OH), 5.16-5.08 (2H, m, CHCH_2), 3.98 (3H, s, OCH_3), 3.50-3.44 (1H, m, quinuclidine 6-H_A), 3.37 (3H, s, OCH_3), 3.12-3.10 (1H, m, quinuclidine 2-H), 3.04-2.92 (2H, m, quinuclidine 6-H_B and quinuclidine 7-H_A), 2.90-2.83 (1H, m, quinuclidine 7-H_B), 2.40-2.33 (1H, m, quinuclidine 5-H), 2.25-2.18 (1H, m, quinuclidine 8-H_A), 1.76 (1H, s, quinuclidine 4-H), 1.67-1.52 (2H, m, quinuclidine 3-H_A and quinuclidine 3-H_B), 1.19-1.11 (1H, m, quinuclidine 8-H_B). **^{13}C NMR (**126 MHz, CD_3OD) δ 159.9 (quinoline 6-C), 148.2 (quinoline 2-C), 146.1 (Ar-C_q), 145.1 (Ar-C_q), 141.3 (*C*HCH_2), 131.5 (quinoline 8-C), 128.8 (Ar-C_q), 123.6 (quinoline 7-C), 120.1 (quinoline 3-C), 115.4 (CHCH_2), 102.2 (quinoline 5-C), 60.6 (quinuclidine 2-C), 57.6 (OCH_3), 56.4 (OCH_3), 50.8 (quinuclidine 6-C or quinuclidine 7-C), 50.4 (quinuclidine 6-C or quinuclidine 7-C), 40.9 (quinuclidine 5-C), 29.5 (quinuclidine 4-C), 26.8 (quinuclidine 8-C), 21.6 (quinuclidine 3-C). **HRMS** (ESI): $C_{21}H_{26}O_2N_2$ $[M+H]^+$; calculated: 339.2067, found: 339.2068. $[\boldsymbol{\alpha}]_{\mathbf{20}}^{\boldsymbol{D}}$ +221 (c. 0.1, MeOH).

(*1S*,*2R*,*4S*,*5R*)-5-ethenyl-2-[(*S*)-[2-(4-fluorophenyl)-6-methoxyquinolin-4-yl](methoxy)methyl]-1-azabicyclo[2.2.2]octan-1-ium trifluoroacetate (21)

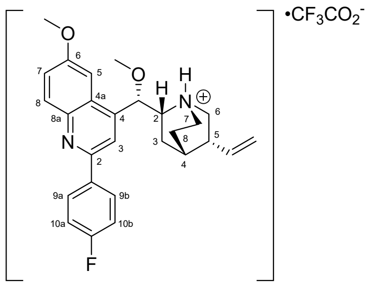

Prepared according to the CH-activation procedure using the methylated quinidine **28** (200 mg, 0.59 mmol, 1 eq.). Preparative HPLC gave the title compound (31.0 mg, 56.7 µmol, 10%) as yellow solid. **^{1}H NMR** (500 MHz, CD_3OD, NH^+ not observed) δ 8.19 (1H, d, *J* 9.3, quinoline 8-H), 8.18-8.14 (2H, m, 9-H a and b), 8.13 (1H, s, quinoline 3-H), 7.62 (1H, dd, *J* 9.3, 2.6, quinoline 7-H), 7.57 (1H, s, quinoline 5-H), 7.38-7.34 (2H, m, 10-H a and b), 6.15-6.07 (1H, m, C*H*CH_2), 5.94 (1H, s, C*H*OCH_3), 5.31-5.28 (1H, m, CHCH_AH), 5.28-5.26 (1H, m, CHCHH_B), 4.06 (3H, s, OCH_3), 4.06-4.01 (1H, m, quinuclidine 6-H_A), 3.78-3.73 (1H, m, quinuclidine 2-H), 3.61-3.54 (2H, m, quinuclidine 6-H_B and quinuclidine 7-H_A), 3.52 (3H, s, OCH_3), 3.39-3.33 (1H, m, quinuclidine 7-H_B), 2.81-2.75 (1H, m, quinuclidine 5-H), 2.56-2.51 (1H, m, quinuclidine 3-H_A), 2.05 (1H, app s, quinuclidine 4-H), 2.01-1.94 (1H, m, quinuclidine 8-H_A), 1.91-1.84 (1H, m, quinuclidine 8-H_B), 1.43-1.38 (1H, m, quinuclidine 3-H_B). **^{13}C NMR (**126 MHz, CD_3OD; *C*F_3CO_2 not observed) δ 166.7 (Ar-C_q), 165.1 (Ar-C_q), 162.45 (q, J_{CF} 39.0; $CF_3CO_2^-$), 161.1 (Ar-C_q), 153.9 (Ar-C_q), 147.1 (Ar-C_q), 142.0 (Ar-C_q), 138.1 (*C*HCH_2), 135.6, 135.5, 131.5 (d, J_{CF} 8.8, C-9a, C-9b), 129.3 (quinoline 8-C), 127.8 (Ar-C_q), 126.1 (quinoline 7-C), 118.7 (quinoline 3-C), 117.7 (CH*C*H_2), 117.2 (d, J_{CF} 22.6, C-10a, C-10b), 102.4 (quinoline 5-C), 79.1 (*C*HOCH_3), 60.9 (quinuclidine 2-C), 57.9 (OCH_3), 57.0 (OCH_3), 50.4 (quinuclidine 7-C), 49.8 (quinuclidine 6-C), 38.2 (quinuclidine 5-C), 28.7 (quinuclidine 4-C), 23.9 (quinuclidine 8-C), 19.5 (quinuclidine 3-C). **HRMS** (ESI): $C_{27}H_{29}FO_2N_2$ $[M+H]^+$; calculated: 433.2286, found: 433.2281. $[\alpha]_{20}^{D}$ +97 (c. 0.1, MeOH).

***tert*-Butyl N-[2-({2-[(1S,3R,4S,6R)-6-[(S)-[2-(4-fluorophenyl)-6-methoxyquinolin-4-yl](hydroxy)methyl]-1-azabicyclo[2.2.2]octan-3-yl]ethyl}sulfanyl)ethyl]carbamate (23)**

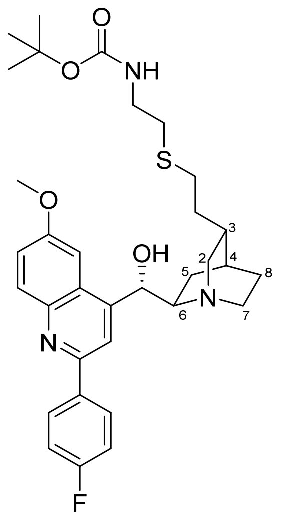

Autoquin (163 mg, 0.31 mmol, 1.0 eq., TFA salt used) was suspended in neat 2-(Boc-amino)ethanethiol (1 mL, 5.64 mmol, 18 eq.) in a Supelco 7 mL vial fitted with a screw cap bearing a PTFE/silicone septum and equipped with a magnetic stirrer bar. The reaction mixture was stirred and flushed with Ar (needle used as a gas outlet) for 10 min. The reaction mixture was then securely sealed with parafilm and transferred to a pre-heated heating block at 80 °C (the solvent level in the vial was submerged below the heating block level). The reaction mixture was stirred at this temperature for 60 h. The reaction mixture was cooled, then directly loaded (CH_2Cl_2) onto a SiO_2 column (pre-slurried in 5% {50:8:1 CH_2Cl_2:EtOH:$NH_4OH_{(sat.; aq.)}$} in CH_2Cl_2). Flash column chromatography eluting with 5% {50:8:1 CH_2Cl_2:EtOH:$NH_4OH_{(sat.; aq.)}$} in CH_2Cl_2 (until all the unreacted thiol had come off [TLC monitoring]) then 33% {50:8:1 CH_2Cl_2:EtOH:$NH_4OH_{(sat.; aq.)}$} in CH_2Cl_2 gave the *title compound* **23** (177 mg, 0.30 mmol, 97%) as a pale orange glass.

^{1}H NMR (700 MHz, CD_3OD, NH and OH not observed): δ = 8.12 – 8.08 (m, 3H, phenyl 2-H and quinoline 3-H), 8.01 (d, J = 9.2, 1H, quinoline 8-H), 7.41 (dd, J = 9.2, 2.5, 1H, quinoline 7-H), 7.34 (d, J = 2.5, 1H, quinoline 5-H), 7.23 (app. t, J = 8.8, 2H, phenyl 3-H), 5.87 (s, 1H, C*H*(OH)), 3.97 (s, 3H, OCH_3), 3.77 – 3.68 (m, 1H, 2-H_A), 3.42 – 3.34 (m, 1H, 6-H), 3.25-3.16 (4H, m, includes 2-H_B, 7-H_A and at d 3.21: t, J = 7.1, 2H, *C*H_2NHBoc), 3.25 – 3.15 (m, 1H, 7-H_B), 2.64 – 2.52 (m, 4H, 2 × SCH_2), 2.38 – 2.29 (m, 1H, 5-H_A), 2.03 – 1.79 (m, 4H, CHC*H*$_2$$CH_2$S, 3-H, 4-H), 1.74 – 1.64 (m, 1H, 8-H_A and 8-H_B), 1.39 (s, 9H, $C_q(CH_3)_3$), 1.22 – 1.13 (m, 1H, 5-H_B); **^{13}C NMR** (126 MHz, CD_3OD): δ = 165.12 (d, J = 247.6, phenyl 4-C),

159.81 (Ar-C_q), 158.43 (Ar-C_q), 155.13 (NH(CO)O), 148.84 (Ar-C_q), 145.39 (Ar-C_q), 137.18 (d, J = 3.1, phenyl 1-C), 132.28 (quinoline 8-C), 130.58 (d, J = 8.4, phenyl 2-C), 126.45 (Ar-C_q), 123.50 (quinoline 7-C), 117.68 (quinoline 3-C), 116.68 (d, J = 21.9, phenyl 3-C), 102.09 (quinoline 5-C), 80.12 ($OC_q(CH_3)_3$), 70.68 (CH(OH)), 61.12 (6-C), 56.58 (OCH_3), 51.45 (2-C), 50.74 (7-C), 41.30 (*C*H_2NHBoc), 34.51 (3-C), 32.97 (CH*C*H_2CH_2), 32.65 (SCH_2), 30.31 (SCH_2), 28.76 ($C_q(CH_3)_3$), 27.03 (4-C), 26.03 (8-C), 19.87 (5-C); **HR-ESI-MS**: *m/z* = 596.2951 ($[M + H]^+$, calcd. for $C_{33}H_{43}O_4N_3FS$: 596.2953) (Δ = –0.3655 ppm); $[\alpha]_{20}^{D}$ = +346 (c. 0.1, MeOH).

(*S*)-[(*1S,2R,4S,5R*)-5-{2-[(2-Aminoethyl)sulfanyl]ethyl}-1-azabicyclo[2.2.2]octan-2-yl][2-(4-fluorophenyl)-6-methoxyquinolin-4-yl]methanol

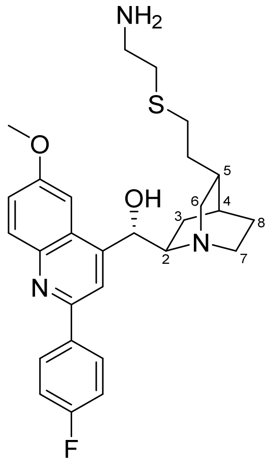

Compound **23** (37 mg, 62 µmol, 1.0 eq.) was dissolved in CH_2Cl_2 (3 mL), and TFA (1.5 mL) was added at rt. The RM was stirred for 1 h, then concentrated *in vacuo*. The crude reaction product was purified by flash column chromatography eluting with 50-100% {50:8:1 CH_2Cl_2:EtOH:$NH_4OH_{(sat.; aq.)}$} in CH_2Cl_2 to give the *title compound* **27** (23 mg, 46 µmol, 75%) as a pale yellow oil.

^{1}H NMR (700 MHz, CD_3OD, NH_2 and OH not observed): δ = 8.11 (dd, J = 8.8, 5.4, 2H, phenyl 2-H), 8.08 (s, 1H, quinoline 3-H), 8.02 (d, J = 9.2, 1H, quinoline 8-H), 7.41 (dd, J = 9.2, 2.7, 1H, quinoline 7-H), 7.37 (d, J = 2.7, 1H, quinoline 5-H), 7.26 (app. t, J = 8.8, 2H, phenyl 3-H), 5.67 (d, J = 2.6, 1H, C*H*(OH), 3.98 (s, 3H, OCH_3), 3.41 (app. ddd, J = 13.0, 7.6, 1.5, 1H, 6-H_A), 3.08 (app. t, J = 9.0, 1H, 2-H), 2.99 – 2.89 (m, 2H includes 7-H_A; and at δ 2.96, dd, J = 13.0, 9.8, 1H, 6-H_B), 2.87 – 2.77 (m, , 3H; includes 7-H_B; and at δ 2.84: t, J = 6.6, 2H, *C*H_2NH_2),

2.67 (t, J = 6.7, 2H, SCH_2CH$_2$NH$_2$), 2.61 – 2.51 (m, 2H, CHCH$_2$CH_2S), 2.21 (dd, J = 12.3, 9.6, 1H, 3-H$_A$), 1.95 – 1.69 (m, 4H, CHCH_2CH$_2$S, 4-H, 5-H), 1.56 (dd, J = 7.6, 6.0, 8-H$_A$ and 8-H$_B$, 2H), 1.13 – 1.06 (m, 1H, 3-H$_B$); **^{13}C NMR** (126 MHz, CD$_3$OD): δ = 165.06 (d, J = 247.5, phenyl 4-C), 159.54 (Ar-C$_q$), 155.20 (Ar-C$_q$), 150.94 (Ar-C$_q$), 145.33 (Ar-C$_q$), 137.38 (d, J = 3.1, phenyl 1-C), 132.04 (quinoline 8-C), 130.58 (d, J = 8.4, phenyl 2-C), 126.77 (Ar-C$_q$), 123.39 (quinoline 7-C), 117.52 (quinoline 3-C), 116.64 (d, J = 21.9, phenyl 3-C), 102.26 (quinoline 5-C), 72.62 (CH(OH)), 60.74 (2-C), 56.40 (OCH$_3$), 51.57 (6-C), 50.98 (7-C), 41.53 (*C*H$_2$NH$_2$), 35.71 (S*C*H$_2$CH$_2$NH$_2$), 35.37 (5-C), 33.58 (CH*C*H$_2$CH$_2$S), 30.47 (CHCH$_2$*C*H$_2$S), 27.79 (8-C), 27.75 (4-C), 20.81 (3-C); **HR-ESI-MS**: *m/z* = 496.2424 ([*M* + H]$^+$, calcd. for $C_{28}H_{35}FN_3O_2S$: 496.2429) (Δ = –0.9553 ppm); $[\alpha]_{20}^{D}$ = +145 (c. 1.5, MeOH).

(*1S,2R,4S,5R*)-5-{2-[(2-Aminoethyl)sulfanyl]ethyl}-2-[(*S*)-[2-(4-fluorophenyl)-6-methoxyquinolin-4-yl](hydroxy)methyl]-1-azabicyclo[2.2.2]octan-1-ium trifluoroacetate

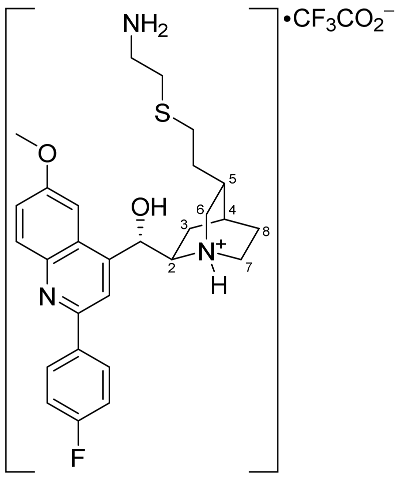

Compound **23** (177 mg, 0.30 mmol, 1.0 eq.) was dissolved in CH_2Cl_2 (5 mL), and TFA (5 mL) was added at rt. The RM was stirred for 1 h, then concentrated *in vacuo*. The crude reaction product was purified by mass-directed preparative HPLC (10-100% MeCN in H_2O + 0.1 % TFA) to give the *title compound* (140 mg, 0.23 mmol, 77%) as a pale yellow oil.

^{1}H NMR (500 MHz, CD$_3$OD, NH$^+$, NH$_2$ and OH not observed): δ = 8.31 (s, 1H, quinoline 3-H), 8.21 – 8.14 (m, 3H, includes phenyl 2-H; and at δ 8.19: d, J = 9.4, 1H, quinoline 8-H), 7.64 (dd, J = 9.4, 2.3, 1H, quinoline 7-H), 7.52 (d, J = 2.3, 1H, quinoline 5-H), 7.38 (app. t, J = 8.7, 2H, phenyl 3-H), 6.24 (s, 1H, C*H*(OH)), 4.13 – 4.02 (m, 4H, includes 6-H$_A$; and at δ 4.06: s, 3H, OCH$_3$), 3.75 (app. t, J = 9.5, 1H, 2-H), 3.62 – 3.51 (m, 2H, 6-H$_B$ and 7-H$_A$), 3.40 – 3.33

(m, 1H, 7-H_B), 3.17 (t, J = 6.8, 2H, C$H_2$$NH_2$), 2.86 (t, J = 6.8, 2H, SC$H_2$$CH_2$$NH_2$), 2.73 – 2.62 (m, 2H, CHCH_2CH_2S), 2.53 (dd, J = 12.4, 10.4, 1H, 3-H_A), 2.29 – 2.16 (m, 1H, 5-H), 2.04 (br. s, 1H, 4-H), 2.03 – 1.83 (m, 4H, CHC$H_2$$CH_2$S, 8-$H_A$ and 8-H_B), 1.39 – 1.27 (m, 1H, 3-H_B); **^{13}C NMR** (126 MHz, CD_3OD): δ = 165.99 (d, J = 250.6, phenyl 4-C), 162.24 (q, J = 36.4, CF_3*C*O_2^-), 161.09 (Ar-C_q), 153.68 (Ar-C_q), 151.86 (Ar-C_q), 141.09 (Ar-C_q), 133.51 (d, J = 2.3, phenyl 1-C), 131.59 (d, J = 8.9, phenyl 2-C), 128.78 (quinoline 8-C), 126.83 (quinoline 7-C), 126.12 (Ar-C_q), 119.01 (quinoline 3-C), 117.74 (q, J = 291.0, *C*$F_3$$CO_2^-$), 117.34 (d, J = 22.3, phenyl 3-C), 102.40 (quinoline 5-C), 68.89 (CH(OH)), 61.07 (2-C), 56.95 (OCH_3), 51.13 (6-C), 50.44 (7-C), 39.71 (*C*$H_2$$NH_2$), 33.49 (5-C), 32.11 (CH*C*$H_2$$CH_2$S), 29.83 ($SCH_2$), 29.74 ($SCH_2$), 26.56 (4-C), 24.5 (8-C), 18.72 (3-C); **HR-ESI-MS**: *m/z* = 496.2438 ([*M* + H]$^+$, calcd. for $C_{28}H_{35}FN_3O_2S$: 496.2429) (Δ = +1.9617 ppm); $[\alpha]_{20}^{D}$ = +83 (c. 0.1, MeOH).

(*1S,2R,4S,5R*)-5-(3-{[2-(3-{2-[2-(2-Aminoethoxy)ethoxy]ethoxy}propanamido)ethyl]sulfanyl}propyl)-2-[(*S*)-[2-(4-fluorophenyl)-6-methoxyquinolin-4-yl](hydroxy)methyl]-1-azabicyclo[2.2.2]octan-1-ium trifluoroacetate (25)

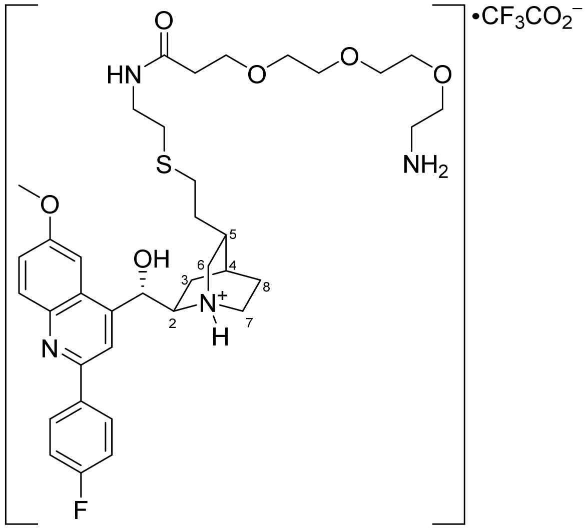

Compound **23** (70 mg, 0.12 mmol, 1.0 eq.) was dissolved in CH_2Cl_2 (2.5 mL) and TFA (2.5 mL) was added at rt. The RM was stirred for 1 h. The reaction mixture was concentrated *in vacuo* to give the crude amine TFA salt which was subjected to the next steps without further purification.

Amine TFA salt was taken up in DMA (1.0 mL) and Boc-NH-PEG(3)-CO_2H (60 mg, 0.19 mmol, 1.60 eq.), TBTU (60 mg, 0.19 mmol, 1.60 eq.) and DIPEA (72 µL, 0.41 mmol, 3.5 eq.) were added. The reaction mixture was stirred for 17 h. The reaction mixture was directly subjected to purification preparative HPLC (10-100% MeCN in H2O + 0.1 % TFA), isolating 80 mg yellow oil with MH^+= 800. Following concentration of the appropriate fractions, analysis of the isolated product by 300 MHz NMR spectroscopy identified a 70:30 mixture of Boc/deBoc products. The yellow oil (80 mg) was dissolved in CH_2Cl_2 (2.5 mL) and TFA (2.5 mL) was added at rt. The RM was stirred for 1 h. The reaction mixture was concentrated *in vacuo* to give the *title product* **25** (83 mg, 0.10 mmol, 87%) as a yellow oil.

^{1}H NMR (500 MHz, CD_3OD, NH^+, NH, NH_2 and OH not observed): δ = 8.36 (s, 1H, quinoline 3-H), 8.22 (d, J = 9.3, 1H, quinoline 8-H), 8.20 – 8.15 (m, 2H, phenyl 2-H), 7.69 (dd, J = 9.3, 2.5, 1H, quinoline 7-H), 7.57 (d, J = 2.5, 1H, quinoline 5-H), 7.41 (t, J = 8.7, 2H, phenyl 3-H), 6.26 (s, 1H, C*H*(OH)), 4.11 – 4.01 (m, 4H, includes 6-H_A; and at δ 4.07, s, 3H, OCH_3), 3.80 –

3.50 (m, 15H, includes 2-H, 6-H_B, 7-H_A, and 6 × OCH_2), 3.40 (t, J = 7.0, 2H, C*H*$_2$NH(CO)), 3.38 – 3.33 (m, 1H, 7-H_B), 3.13 (t, J = 5.0, 2H, CH_2NH_2), 2.72 – 2.58 (m, 4H, includes $CHCH_2CH_2S$; and at δ 2.69: t, J = 7.0, 2H, SC*H*$_2$CH$_2$NH(CO)), 2.58 – 2.49 (m, 1H, 3-H_A), 2.47 (t, J = 6.2, 2H, NH(CO)C*H*$_2$), 2.26 – 2.17 (m, 1H, 5-H), 2.05 (br. s, 1H, 4-H), 2.03 – 1.83 (m, 4H, CHC*H*$_2$CH$_2$S and 8-H), 1.38 – 1.27 (m, 1H, 3-H_B); **^{13}C NMR** (126 MHz, CD_3OD): δ = 173.97 (NH(CO)), 166.18 (d, J = 251.1, phenyl 4-C), 161.68 (q, J = 37.3, $CF_3CO_2^-$), 161.31 (Ar-C_q), 153.35 (Ar-C_q), 152.95 (Ar-C_q), 140.06 (Ar-C_q), 132.66 (d, J = 1.3, phenyl 1-C), 131.86 (d, J = 8.9, phenyl 2-C), 127.91 (quinoline 8-C), 127.00 (quinoline 7-C), 126.64 (Ar-C_q), 119.37 (quinoline 3-C), 117.49 (d, J = 22.3, phenyl 3-C), 117.48 (q, J = 289.8, $CF_3CO_2^-$), 102.61 (quinoline 5-C), 71.52 (OCH_2), 71.40 (OCH_2), 71.20 (OCH_2), 71.14 (OCH_2), 68.96 (CH(OH), 68.25 (OCH_2), 67.87 (OCH_2), 61.04 (2-C), 57.03 (OCH_3), 51.22 (6-C), 50.49 (7-C), 40.60 (CH_2NH_2), 40.08 (CH_2NH(CO)), 37.49 (NH(CO)CH_2), 33.51 (5-C), 32.47 (C*H*CH$_2$CH$_2$S), 32.03 (S*C*H$_2$CH$_2$NH(CO)), 29.91 (CHCH$_2$*C*H$_2$S), 26.57 (4-C), 24.48 (8-C), 18.79 (3-C); **HR-ESI-MS**: *m/z* = 721.3407 ([*M* + H]$^+$, calcd. for $C_{37}H_{52}FN_4O_6S$: 721.3406) (Δ = +0.2165 ppm); $[\alpha]_{20}^{D}$ = +74 (c. 0.1, MeOH).

***tert*-Butyl *N*-[2-({3-[(*1S*,*3R*,*4S*,*6R*)-6-[(*S*)-hydroxy(6-methoxyquinolin-4-yl)methyl]-1-azabicyclo[2.2.2]octan-3-yl]propyl}sulfanyl)ethyl]carbamate (22)**

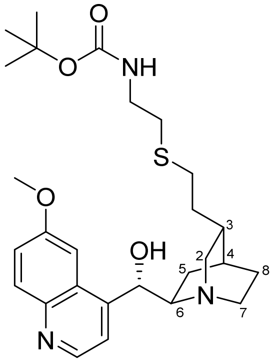

Quinidine (100 mg, 0.31 mmol, 1.0 eq.) was suspended in neat 2-(Boc-amino)ethanethiol (1 mL, 5.64 mmol, 18 eq.) in a Supelco 7 mL vial fitted with a screw cap bearing a PTFE/silicone septum and equipped with a magnetic stirrer bar. The reaction mixture was stirred and flushed with Ar (needle used as a gas outlet) for 10 min. The reaction mixture was then securely sealed with parafilm and transferred to a pre-heated heating block at 80 °C (the solvent level in the vial was submerged below the heating block level). The reaction mixture was stirred at this temperature for 60 h. The reaction mixture was cooled, then directly loaded (CH_2Cl_2) onto a

SiO_2 column (pre-slurried in 5% {50:8:1 CH_2Cl_2:EtOH:$NH_4OH_{(sat.;\ aq.)}$} in CH_2Cl_2). Flash column chromatography eluting with 5% {50:8:1 CH_2Cl_2:EtOH:$NH_4OH_{(sat.;\ aq.)}$} in CH_2Cl_2 (until all the unreacted thiol had come off [TLC monitoring]) then 33% {50:8:1 CH_2Cl_2:EtOH:$NH_4OH_{(sat.;\ aq.)}$} in CH_2Cl_2 gave the *title compound* **14** (38 mg, 75.8 μmol, 25%) as a colourless oil.

^{1}H NMR (500 MHz, CD_3OD, NH and OH not observed): δ = 8.68 (d, 1H, J = 4.7, quinoline 2-H), 7.95 (d, 1H, J = 9.2, quinoline 8-H), 7.73 (d, 1H, J = 4.7, quinoline 3-H), 7.43 (dd, 1H, J = 9.2, 2.7, quinoline 7-H), 7.38 (d, 1H, J = 2.7, quinoline 5-H), 5.75 (app. s, 1H, C*H*(OH)), 3.98 (s, 3H, OCH_3), 3.58 – 3.51 (m, 1H, 2-H_A), 3.24 (t, J = 7.1, 2H, C*H*$_2$NHBoc), 3.22 – 3.15 (m, 1H, 6-H), 3.12 – 3.01 (m, 2H, 2-H_B and 7-H_A), 2.97 – 2.88 (m, 1H, 7-H_B), 2.66 – 2.61 (m, 2H, SC*H*$_2$$CH_2$NHBoc), 2.61 – 2.54 (m, 2H, $CHCH_2$C*H*$_2$S), 2.25 (dd, J = 13.2, 9.1, 1H, 5-H_A), 1.94 – 1.80 (m, 3H, 3-H and CHC*H*$_2$$CH_2$S) 1.79 (br. s, 4-H), 1.67 – 1.58 (m, 2H, 8-H_A and 8-H_B), 1.44 (s, 9H, $OC(CH_3)_3$), 1.15 – 1.07 (m, 1H, 5-H_B) **^{13}C NMR** (126 MHz, CD_3OD): δ = 159.79 (C_q), 158.41 (C_q), 149.73 (Ar-C_q), 148.18 (quinoline 2-C), 144.72 (Ar-C_q), 131.43 (quinoline 8-C), 127.90 (Ar-C_q), 123.45 (quinoline 7-C), 120.03 (quinoline 3-C), 102.22 (quinoline 5-C), 80.11 (O*C*$_q$$(CH_3)_3$), 71.49 (CH(OH)), 60.86 (6-C), 56.49 (OCH_3), 51.51 (2-C), 50.86 (7-C), 41.34 (CH_2NHBoc), 35.11 (3-C), 33.26 (CH*C*H_2CH_2S), 32.62 ($CHCH_2$*C*H_2S), 30.48 (S*C*H_2CH_2NHBoc), 28.77 (OC(*C*H_3)$_3$), 27.39 (4-C), 26.98 (8-C), 20.41 (5-C); **HR-ESI-MS**: *m/z* = 502.2729 ([*M* + H]$^+$, calcd. for $C_{27}H_{40}O_4N_3S$: 502.2734) (Δ = –1.0539 ppm); $[\boldsymbol{\alpha}]_{\mathbf{20}}^{\boldsymbol{D}}$ = +19.0 (c. 1.0, MeOH).

(*1S,2R,4S,5R*)-5-(3-{[2-(3-{2-[2-(2-Aminoethoxy)ethoxy]ethoxy}propanamido)ethyl]sulfanyl}propyl)-2-[(*S*)-hydroxy(6-methoxyquinolin-4-yl)methyl]-1-azabicyclo[2.2.2]octan-1-ium trifluoroacetate (24)

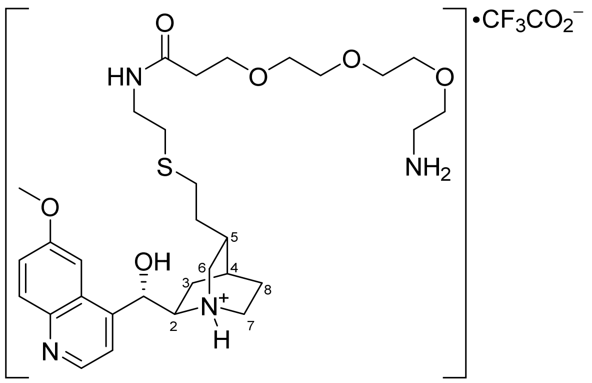

Compound **22** (36 mg, 72 µmol, 1.0 eq.) was dissolved in CH_2Cl_2 (2.5 mL) and TFA (2.5 mL) was added at rt. The RM was stirred for 1 h. The reaction mixture was concentrated *in vacuo* to give the crude amine TFA salt which was subjected to the next steps without further purification.

The amine TFA salt was taken up in DMA (1.0 mL) and Boc-NH-PEG(3)-CO_2H (37 mg, 0.11 mmol, 1.60 eq.), TBTU (37 mg, 0.11 mmol, 1.60 eq.) and DIPEA (44 µL, 0.25 mmol, 3.5 eq.) were added. The reaction mixture was stirred for 17 h. The reaction mixture was directly subjected to purification preparative HPLC (10-100% MeCN in H_2O + 0.1 % TFA), isolating 98 mg yellow oil with MH^+= 705; and MNa^+= 727. Following concentration of the appropriate fractions, analysis of the isolated product by 300 MHz NMR spectroscopy identified a mixture of Boc/deBoc products. The yellow oil (98 mg) was dissolved in CH_2Cl_2 (2.5 mL) and TFA (2.5 mL) was added at rt. The RM was stirred for 1 h. The reaction mixture was concentrated *in vacuo*. The reaction mixture purified by preparative HPLC (10-100% MeCN in H2O + 0.1 % TFA), isolating the *title compound* **16** (44 mg, 61 µmol, 85%) as a colourless oil.

^{1}H NMR (700 MHz, CD_3OD, NH^+, NH, NH_2 and OH): δ = 8.99 (d, J = 5.5, 1H, quinoline 2-H), 8.27 (d, J = 5.5, 1H, quinoline 3-H), 8.20 (d, J = 9.3, 1H, quinoline 8-H), 7.80 (dd, J = 9.3, 2.6, 1H, quinoline 7-H), 7.66 (d, J = 2.6, 1H, quinoline 5-H), 6.33 (s, 1H, CH(OH)), 4.09 (s, 3H, OCH_3), 4.03 (ddd, J = 12.6, 8.3, 2.5, 1H, 6-H_A), 3.75 (t, J = 6.2, 2H, OCH_2), 3.73 – 3.70 (m, 3H, OCH_2 and 2-H), 3.69 – 3.64 (m, 6H, 3 × OCH_2), 3.64 – 3.61 (m, 2H, OCH_2), 3.59 – 3.55 (m, 1H, 6-H_B), 3.55 – 3.51 (m, 1H, 7-H_A), 3.41 (t, J = 7.0, 2H, C*H*$_2$NH(CO)), 3.38 – 3.32 (m, 1H, 7-H_B), 3.14 (t, J = 5.0, 2H, C*H*$_2$$NH_2$), 2.69 (t, J = 7.2, 2H, SC*H*$_2$$CH_2$NH(CO)), 2.68 –

2.60 (m, 2H, $CHCH_2CH_2S$), 2.50 – 2.45 (m, 3H, includes 3-H_A and at δ 2.48: app. t, J = 6.2, 2H, NH(CO)CH_2), 2.25 – 2.19 (m, 1H, 5-H), 2.05 (br. s, 1H, 4-H), 2.01 – 1.84 (m, 4H, 8-H_A, 8-H_B, and $CHCH_2CH_2S$), 1.31 – 1.25 (m, 1H, 3-H_B); **^{13}C NMR** (176 MHz, CD_3OD): δ = 173.99 (CO), 162.19 (Ar-C_q), 161.90 (q, J 35.8, $CF_3CO_2^-$), 156.17 (Ar-C_q), 142.87 (quinoline 2-C), 136.59 (Ar-C_q), 128.81 (quinoline 7-C), 128.45 (Ar-C_q), 125.30 (quinoline 8-C), 121.12 (quinoline 3-C), 116.44 (q, J 295.8, $CF_3CO_2^-$), 102.90 (quinoline 5-C), 71.53 (OCH_2), 71.42 (OCH_2), 71.23 (OCH_2), 71.16 (OCH_2), 68.75 (CH(OH)), 68.26 (OCH_2), 67.88 (OCH_2), 60.76 (2-C), 57.33 (OCH_3), 51.21 (6-C), 50.51 (7-C), 40.63 (CH_2NH_2), 40.12 (CH_2NH(CO)), 37.52 (NH(CO)CH_2), 33.49 (5-C), 32.52 ($CHCH_2CH_2S$), 32.07 (SCH_2CH_2NH(CO)), 29.94 ($CHCH_2CH_2S$), 26.59 (4-C), 24.43 (8-C), 18.69 (3-C); **HR-ESI-MS**: *m/z* = 605.3367 ([M + H]$^+$, calcd. for $C_{31}H_{49}O_6N_4S$: 605.3367) (Δ = –0.0105 ppm); $[\boldsymbol{\alpha}]_{20}^{D}$ = +86.3 (c. 1.0, MeOH).

Cell Biological Experimental

Cell Culture and Transfection

HEK293T (ATCC) and HeLa (ATCC) cells were cultured in Dulbecco's modified Eagle's medium (Sigma) supplemented with 10% fetal calf serum, 1% penicillin/streptomycin, and non-essential amino acids at 37°C with 5% CO_2. MCF7 cells stably transfected with EGFP-LC3 (MCF7-eGFP-LC3) were cultured at 37 °C with 5% CO_2 using Eagle's MEM (PAN Biotech cat# P04-08500) containing 10% FBS (Invitrogen cat# 10500-084), 1% sodium pyruvate (PAN Biotech cat# P04-43100), 1% NEAA (PAN Biotech cat# P08-32100), 0.01 mg/ml bovine insulin (Sigma Aldrich cat# I9278) and 200 µg/ml G418 as the medium. Untransfected MCF7 cells were incubated in the same media without G418. Cells were tested for mycoplasma contamination bimonthly using the MycoAlertTM mycoplasma detection kit (Lonza).

The human mammary epithelial cell line infected with a retrovirus carrying hTERT, SV40 and the oncogenic allele *HrasV12*, named HMLER cells were a generous gift from A. Puisieux. HMLER cells were cultured in DMEM/F12 supplemented with 10% FBS, 10 µg/mL insulin, 0.5 µg/mL hydrocortisone, 10 ng/mL hEGF, and 0.5 µg/mL puromycin.

High-content screening for autophagy inhibitors

The phenotypic autophagy screen utilizes MCF7-eGFP-LC3 cells. 4000 cells per well were seeded in 25 µl medium in a 384 well Greiner µclear plate (cat# 781080, lid cat# 656191) and incubated (37 °C, 5% CO2) overnight. Cells were then washed by a plate washer (Biotek, ELx405) three times with 1X PBS followed by a final aspiration of the washing buffer. The addition of 25 nl of compound solution (10 mM stock solution in DMSO) was then carried out with an echo dispenser (Labcyte, Echo 520 dispenser). Addition of medium to induce autophagy was carried out with a Multidrop Combi (Thermo Scientific). 25 µl EBSS (Sigma Aldrich, cat# E3024-500ml) containing 50 µM Chloroquine (Sigma Aldrich, cat# C6628-25g) was used for starvation-induced autophagy and 25 µl medium containing 50 µM Chloroquine and 100 nM Rapamycin (Biomol, cat# Cay13346)-1 was used for rapamycin-induced autophagy screening. After incubation (37 °C, 5% CO_2) for three hours cells were fixed by addition of 25 µl 1:4 formaldehyde in 1X PBS + 1:500 Hoechst (stock: 1 mg/ml, Sigma Aldrich

cat# B2261-25mg) and incubation for 20 min at room temperature. Cells were then washed three times with 1X PBS. Four images per well were taken with ImageXpress Micro XL (Molecular Devices) at 20x. Automated image analysis was performed using the granularity setting of MetaXpress Software (Molecular Devices). The most significant analysis parameter was granule area; with resulting signal-to-background ratios around 40 and Z' values around 0.7.

Antibodies

Anti-p62/SQSTM1 was purchased from MBL international (Cat# PM045) and used at 1:10000. Anti-LC3B was obtained from Cell Signaling Technology (Cat# 2775) and used at 1:1000. Anti-beta-actin was purchased from abcam (Cat# ab8227) and used at 1:10,000. Goat anti-rabbit-HRP was purchased from Pierce (Thermo Scientific, Cat# 31460) and used at a dilution of 1:10,000. Anti-His-tag monoclonal antibody was obtained from Cell Signaling Technology (Cat# 2366) and used at 1:1000. Goat anti-mouse HRP secondary antibody was purchased from Thermo Fisher (Cat# 31430) and used at 1:10,000.

Immunoblotting

200,000 MCF7-eGFP-LC3 cells in 2 mL media were seeded in 6-well plates and incubated (37 °C, 5% CO_2) overnight. The media was removed and the cells were washed with PBS (1X), before adding test compounds at the required concentrations in EBSS. Cells were incubated (37 °C, 5% CO_2) for 3 h before removing the media, washing with PBS (1X), and lysing in SDS loading buffer without bromophenol blue. Protein concentrations were determined using the DC Assay (Bio-rad) according to the manufacturer's instructions. SDS-PAGE was carried out using 15% polyacrylamide gels run at a constant voltage of 80 V for 15 min followed by 120 V for approximately 2 h. Semi-dry transfer onto a PVDF membrane was performed at 25 V for 45 min. Membranes were blocked in 5% milk in TBST (blocking buffer) for 1 h at room temperature. The membrane was incubated with the primary antibody in blocking buffer overnight at 4 °C. After washing with TBST (3 x 5 min) the membrane was incubated with the secondary antibody in blocking buffer for one hour at room temperature. Signals were visualized using the SuperSignal West Pico Chemiluminescent Substrate or the SuperSignal West Femto Maximum Sensitivity Substrate (Thermo Fischer) on a Li-COR Odyssey Fc.

Alternatively, cells were lysed in ice-cold lysis buffer (20 mM Tris-HCl pH 8, 300 mM KCl, 10% Glycerol, 0.25% Nonidet P-40, 0.5 mM EDTA, 0.5 mM EGTA, 1 mM PMSF, 1x complete protease inhibitor (Roche)), passed 5x through a 21G needle, and cleared by centrifugation. Protein concentrations were determined using Bio-Rad Protein Reagent (Bio-Rad). Lysates were then mixed with 2x sample buffer and boiled for 10 min prior to separation by SDS-PAGE and transferred to a nitrocellulose membrane (GE). Membranes were incubated with primary antibody overnight at 4°C, washed with TBST and incubated for 1 h at room temperature with the appropriate HRP-conjugated secondary antibody in blocking buffer (5% skim milk in TBST). Protein detection was carried out using chemiluminescence (Thermo Scientific) and radiographic film (Santa-Cruz).

Affinity enrichment with NHS-activated beads

Preparation of MCF7/LC3 Cell Lysate

MCF7 cells stably transfected with eGFP-LC3 were cultivated according to previously described procedure until they reached 80 to 90% confluence. Subseuqently, the cells were washed with PBS and detaches with cell dissociation solution (Sigma Aldrich cat# C5915). The cells were resuspended in 1X PBS buffer, centrifuged at 350 g for 5 min and washed twice with 1X PBS. The cells were resuspended in lysis buffer (50 mM PIPES (pH 7.4), 50 mM NaCl, 5 mM MgCl2, 5 Mm EGTA, 0.1% (v/v) NP40, 0.1% (v/v) Triton, 0.1% (v/v) Tween, 1 mM DTT, EDTA-free protease inhibitor (Sigma Aldrich cat# 11873580001)) and incubated on ice for 30 min, while gently vortexing every 10 min. The lysate was homogenized by passing it 10 times through a 20 g needle and centrifuged at 4 °C for 20 min at 18,500 g. The protein concentration was determined by means of DCTM protein assay (BIO-RAD cat# 5000112) according to the manufacturer’s instructions. The lysate was diluted with lysis buffer to a finally protein concentration of 4.5 mg/ml and stored at -80 °C until further use.

Affinity Enrichment

The NHS Mag Sepharose beads (GE-Healthcare cat# 28951380) were equilibrated to room temperature. For each sample 25 µl of bead slurry were washed with 500 µl ice cold equilibration buffer (1 mM HCl). The equilibration buffer was removed immediately and replaced by 500 µl of 10 µM free amine compound in coupling buffer (0.15 M

Triethanolaminem, 0.5 M NaCl, pH 8.3). The beads were incubated rotating for 1 h at the ambient temperature, washed with 500 µl blocking buffer A (0.5 M Ethanolamine, 0.5 M NaCl, pH 8.3) and 500 µl blocking buffer B (0.1 M Sodium acetate, 0.5 M NaCl). After 15 min incubation with blocking buffer A rotating at the ambient temperature, the beads were washed each time with 500 µl of blocking buffer B, blocking buffer A and again with blocking buffer B. The beads were equilibrated by addition of 500 µl lysis buffer and incubated with MCF7/LC3 lysate for 2 h rotating at 4 °C. The beads were washed twice for 10 min with 500 µl lysis buffer and twice for 10 min with 1X PBS.

On-bead Digestion

As a control sample 100 µl of 5 pM BSA in 1X PBS were used. To each sample 50 µl of reducing buffer (50 mM Tris, pH 7.5, 8 M urea, 1 mM DTT) were added, followed by 30 min incubation at the ambient teperature shaking at 350 rpm. The beads were treated with 5.5 µl of alkylation solution (50 mM chloroacetamide in reducing buffer) and incubation was continued for another 30 min. Subsequently, 2 µl LysC digestion solution (0.5 g/l in H2O) were added. The samples were incubated shaking at 350 rpm for 1 h at 37 °C and transferred to fresh Eppendorf tubes. The beads were resuspended in 167.5 µl Tris buffer containing 2.5 µl Trypsin digestion solution (0.4 µg/µl in 10 mM HCl), followed by incubation shaking at 37 °C for 1 h. The supernatant was removed and combined with the respective sample of the LysC digestion step. To these mixtures another 5 µl of trypsin digestion solution were added and the samples were incubated at 37 °C shaking overnight at 350 rpm. The reaction was terminated the following day by addition of 2 µl concentrated TFA.

Stage-Tip Purification

By addition of 100 µl methanol the C18 Stage Tips (3M™ Empore™ C18 Extraction Disks, Mfr. No. 2215) were activated. The Stage Tips were washed once with 100 µl buffer B (H2O/ACN 2:8 with 0.1 % formic acid) and twice with 100 µl buffer A (H2O with 0.1 % formic acid). The samples were loaded onto the Stage Tips followed by 1 min incubation at the ambient temperature. The Tips were washed once by addition of 100 µl buffer A and 20 µl buffer B were added. After 1 min incubation the samples were eluted centrifuging at 8,000 rpm for 5 min. The elution step was performed a second time and the combined fractions were dried in a SpeedVac concentrator (Eppendorf) at 30 °C.

Data Evaluation by Nano-LC-MS/MS

Protein identification and relative quantification was performed by separation and analysis on a nano-HPLC/MS/MS. For separation, an UltiMateTM 3000 RSLCnano system (Dionex, Germany) was employed. The MS and MS/MS experiments performed on a Q Exactive Hybrid Quadrupole-Orbitrap PlusTM or HFTM Mass Spectrometer equipped with a nano-spray source (Nanospray Flex Ion Source, Thermo Scientific). All solvents were purchased as LC-MS grade. The lyophilized peptides were resuspended in 20 µl 0.1 % TFA in H2O and 3µl of sample were injected onto a pre-column cartridge (5 µm, 100 Å, 300 µm ID * 5 mM, Dionex, Germany) using 0.1 % TFA in water as eluent with a flow rate of 30 µl/min. In order to desalt the sample, the eluent flow to was directed to waste for 5 min. Subsequently, the sample was back-flushed during the whole analysis from the pre-column to the PepMap100 RSLC C18 nano-HPLC column (2 µm, 100 Å, 75 µm ID × 50 cm, nanoViper, Dionex, Germany). Peptide separation was carried out with the following linear gradient: starting conditions 95% solvent A (0.1% formic acid in H2O) / 5% solvent B (0.1% formic acid in acetonitrile), linear increase to 30% solvent B in 90 min, further linear increase to 60% solvent B in 5 min, further linear increase to 95% solvent B in 5 min, washing with these conditions for 5 min, and re-equilibration to starting conditions. The nano-HPLC was coupled online to the Quadrupole-Orbitrap Mass Spectrometer using a standard coated SilicaTip (ID 20 µm, Tip-ID 10 µM, New Objective, Woburn, MA, USA). Mass range of m/z 300 to 1650 was acquired with a resolution of 70000 (Q-Exactive Plus) or 60000 (Q-Exactive HF) for full scan, followed by up to 10 (Q-Exactive Plus) or 15 (Q-Exactive HF) high energy collision dissociation (HCD) MS / MS scans of the most intense at least doubly charged ions.

A Quadrupole-Orbitrap Mass Spectrometer was coupled online to the nano-PLC employing a standard coated Silica Tip (ID 20 µm, Tip-ID 10 µM, New Objective, Woburn, MA, USA). Mass range of m/z 300 to 1650 was acquired with a resolution of 70000 (Q-Exactive Plus) or 60000 (Q-Exactive HF) for full scan, followed by up to 10 (Q-Exactive Plus) or 15 (Q-Exactive HF) high energy collision dissociation (HCD) MS / MS scans of the most intense at least doubly charged ions. For the three biological replicates data evaluation was carried out separately by means of MaxQuant software including the Andromeda search algorithm and searching the human reference proteome of the Uniprot database[3] For the search full enzymatic digestion was assumed tolerating two miscleavages. Carbamidomethlyation was selected as a fixed protein modification, while N-terminal acetylation and methionine oxidation were chosen as variable modifications. For full mass spectra the mass accuracy was set to 4.5 ppm and to 20 ppm for MS/MS spectra. For peptide and protein identification the false discovery rates were

set to 1%. At least to peptides had to be quantified for those proteins that were selected for further validation. The label-free quantification algorithm implemented in MaxQuant was employed to perform relative quantification of proteins. By means of Perseus software (v.1.6.1.1) further data evaluation was carried out.[4] All those proteins, which were not identified with at least two peptides in at least one of the samples, were filtered off. Furthermore, all known contaminations were excluded. The samples originating from affinity enrichment with the active probe were grouped together, as well as the ones originating from the inactive probe. Label-free quantification (LFQ) intensities were logarithmized (log2) and proteins, which were not three times quantified in at least one of the groups, were filtered off. With small normal distributed values (width 0.3, down shift 1.8) the missing values were imputed, followed by a two sided t-test (s0 = 1, FDR 0.05). Hits were selected according to significant enrichment by the active probe in comparison to the inactive probe in all three replicates.

Competitive Affinity Enrichment

For competitive affinity enrichment, the protein lysate was preincubated for 1 h at 4 °C with the desired concentration of compound, followed by addition to the immobilized probe. The detection was performed by immunoblotting. Therefore, the samples were diluted to a final SDS concentration of 2% with 5X SDS loading buffer (50% v/v Glycerol, 250 mM Tris (pH 6.8), 10% w/v SDS, 500 mM DTE, 360 µM bromophenol blue). Protein denaturation was performed by heating to 95 °C for 5 min. Subsequently, the samples were loaded on a self-cast 15% polyacrylamide gel. Gels were run at a constant voltage of 80 V for 20 min and 120 V for 1.5 h. Semi-dry transfer onto a PVDF membrane was carried out for 45 min at 25 V. Blocking of the membrane was performed with blocking buffer (5% milk powder in TBS-T) for 1 h at the ambient temperature. The membrane was incubated with primary antibody in blocking buffer for 1 h at the ambient temperature and washed three times with TBS-T. Incubation with the secondary antibody in blocking buffer was carried out for 1 h at the ambient temperature and the membrane was washed three times with TBS-T. Super SuperSignal™ West Femto Maximum Sensitivity Substrate (ThermoFisher Scientific cat# 34095) was employed for signal visualization on a LI-COR Odyssey Fc.

Cell painting assay

The described assay follows closely the method described by Bray *et al.*[5]

Initially, 5 µl U2OS medium were added to each well of a 384-well plate (PerkinElmer CellCarrier-384 Ultra). Subsequently, U2OS cell were seeded with a density of 1600 cells per well in 20 µl medium. The plate was incubated for 10 min at the ambient temperature, followed by an additional 4 h incubation (37 °C, 5% CO_2). Compound treatment was performed with the Echo 520 acoustic dispenser (Labcyte) at final concentrations of 10 µM, 3 µM or 1 µM. Incubation with compound was performed for 20 h (37 °C, 5% CO2). Subsequently, mitochondria were stained with Mito Tracker Deep Red (Thermo Fisher Scientific, Cat. No. M22426). The Mito Tracker Deep Red stock solution (1 mM) was diluted to a final concentration of 100 nM in prewarmed medium. The medium was removed from the plate leaving 10 µl residual volume and 25 µl of the Mito Tracker solution were added to each well. The plate was incubated for 30 min in darkness (37 °C, 5% CO2). To fix the cells 7 µl of 18.5 % formaldehyde in PBS were added, resulting in a final formaldehyde concentration of 3.7 %. Subsequently, the plate was incubated for another 20 min in darkness (37 °C, 5% CO2) and washed three times with 70 µl of PBS. (Biotek Washer Elx405). Cells were permeabilized by addition of 25 µl 0.1% Triton X-100 to each well, followed by 15 min incubation (37 °C, 5% CO2) in darkness. The cells were washed three times with PBS leaving a final volume of 10 µl. To each well 25 µl of a staining solution were added, which contains 1% BSA, 50 µl Phalloidin (Thermo Fisher Scientific, A12381), 25 µg/ml Concanavalin A (Thermo Fisher Scientific, Cat. No. C11252), 50 µl/ml Hoechst 33342 (Sigma, Cat. No. B2261-25mg), 15 µl/ml WGA-Alexa594 conjugate (Thermo Fisher Scientific, Cat. No. W11262) and 0.3 µl/ml SYTO 14 solution (Thermo Fisher Scientific, Cat. No. S7576). The plate is incubated for 30 min (37 °C, 5% CO2) in darkness and washed three times with 70 µl PBS. After the final washing step the PBS was not aspirated. The plates were sealed and centrifuged for 1 min at 500 rpm.

The plates were prepared in triplicates with shifted layouts to reduce plate effects and imaged using a Micro XL High-Content Screening System (Molecular Devices) in 5 channels (DAPI: Ex350-400/ Em410-480; FITC: Ex470-500/ Em510-540; Spectrum Gold: Ex520-545/ Em560-585; TxRed: Ex535-585/ Em600-650; Cy5: Ex605-650/ Em670-715) with 9 sites per well and 20x magnification (binning 2).

The generated images were processed with the CellProfiler package (https://cellprofiler.org/) on a computing cluster of the Max Planck Society to extract 1716 cell features (parameters).

Further analysis was performed with custom Python (https://www.python.org/) scripts using the Pandas (https://pandas.pydata.org/) and Dask (https://dask.org/) data processing libraries (separate publication to follow).

In a first step, the data was aggregated as overall medians per well.

A subset of highly reproducible parameters was determined using the procedure described by Woehrmann *et al.*[6] in the following way:

Two biological repeats of one plate containing reference compounds were analyzed. For every parameter, its full profile over each whole plate was calculated. If the profiles from the two repeats showed a similarity >= 0.8 (see below), the parameter was added to the set.

This was only done once and resulted in a set of 579 parameters that was used for all further analyses.

Z-scores were then calculated for each parameter as how many times the MAD of the controls the measured value deviates from the median of the controls:

$$z - score = \frac{value_{meas.} - Median_{Controls}}{MAD_{Controls}}$$

The phenotypic compound profile is then the list of z-scores of all parameters for one compound.

In addition to the phenotypic profile, an induction value was determined for each compound as the fraction of significantly changed parameters, in percent:

$$Induction\ [\%] = \frac{number\ of\ parameters\ with\ abs.\ \ values > 3}{total\ number\ of\ parameters}$$

Similarities of phenotypic profiles were calculated from the correlation distances between two profiles (https://docs.scipy.org/doc/scipy/reference/generated/scipy.spatial.distance.correlation.html;

Similarity = 1 - Correlation Distance) and the compounds with the most similar profiles were determined from a set of 3000 reference compounds that was also measured in the assay.

mCherry-EGFP-LC3 analysis

MCF7 mCherry-EGFP-LC3 cells (described previously (Laraia *et al.*[7])) were cultured in Dulbecco's modified Eagle's medium (DMEM) (Sigma-Aldrich) supplemented with 10% fetal bovine serum, 1% penicillin/streptomycin, non-essential amino acids, and 500 µg/mL G418 at 37°C with 5% CO_2. Cells were routinely tested for mycoplasma contamination using the MycoAlert mycoplasma detection kit (Lonza).

After treatment, cells were fixed with 4% paraformaldehyde for 10 minutes at room temperature, washed with PBS and nuclei counterstained with 4′,6-diamidino-2-phenylindole (DAPI). Immunofluorescence imaging was performed on a Leica SP8 FALCON inverted confocal system (Leica Microsystems) equipped with a HC PL APO 63x/1.40 oil immersion lens. DAPI was excited using a 405 nm Diode laser and EGFP/mCherry using a tuned white light laser. Scanning was performed in line-by-line sequential mode.

Image processing was restricted to brightness/contrast adjustment using ImageJ (NIH). Quantification of autophagosomes/autolysosomes was performed using the Analyze Particles function in imageJ.

Assessment of acid sphingomyelinase and ceramidase activity

HEK 293T cells grown in a six well plate (~300K cells / well) in DMEM containing 5% FCS were treated with desipramine or the indicated compounds at 2 µM or 10 µM concentration or 0.1% DMSO as a control for 16h. 1 µM of the FRET probe TP366[8] for the acid sphingomyelinase assay or 2µM of NBD-ceramide (labeled in sphingosine moiety) for acid ceramidase assay were added as BSA complexes. After an additional incubation for one hour, the media was removed and cells were washed twice with ice-cold PBS. Cells were then scraped with a tip in the presence of 500 µl methanol and transferred in a siliconized tube. The remaining residue was treated in the same way with another 500 µl of methanol and transferred into the same tube. The emulsions were sonicated for 5 minutes and then the liquid was removed in a speed vac apparatus over 5h. The residue was re-suspended in 900 µl of chloroform/methanol 2:1 and centrifuged at 13.000 rpm for 5 minutes. The supernatant was mixed with 300 µl of water and vortexed for 2 minutes. This mixture was centrifuged again at

13,000 rpm for 3 minutes. Finally 250 µl of the lower phase were collected, dried in a speed vac and re-dissolved in 50 µl ethanol. 10µl of this solution were injected into an UHPLC with fluorescence detector (Agilent) using a gradient from 50% MeOH to 85% MeOH over 4 minutes. Substrate cleavage was assessed by recording the fluorescence peaks of products at their specific retention times (NBD-Ceramide or NBD-sphingosine) divided by fluorescence peaks of substrates. Fluorescence was recorded at 535 nm, using an excitation wavelength of 410 nm. In the case of TP366 substrate, NBD fluorescence was measured by using an excitation wavelength of 350 nm.

For assessing acid ceramidase activity in cell lysates, lysates were exposed to compound treatment for 16 h and to the probe for 1 hour, before lipid extraction and UHPLC analysis.

Cell lysates were prepared as follows: HEK 293T Cells were grown in DMEM in 75cm^2 cell culture flask to 90-95% confluency. The cells were harvested using a cell scraper and transferred with the media into a falcon tube and centrifuged at 4°C, for 10 minutes at 4000 rpm. The supernatant was removed and the pellet was re-suspended in 1 ml of PBS. After another centrifugation step at 4°C for 5 minutes at 3000 rpm, the supernatant was removed and the pellet was re-suspended in 75 µl 0.4M sucrose solution containing 2x protease inhibitor cocktail (Roche). This suspension was sonicated at 4°C for 5-10 minutes until an almost clear solution was obtained. Aliquots of this solution were frozen in liquid nitrogen and stored at -20 °C for up to 2 weeks. Enzyme assays were performed in sodium acetate buffer pH 4.5 containing 0.1 % Triton X100 and the respective substrate and usually 10% of the cell lysate.

Assay for detection of lysosomotropic compounds

Compounds were tested for lysosomotropy using the fluorescent dye LysoTracker Red DND-99 (ThermoFisher), which accumulates in acidic organelles, such as lysosomes. 10,000 MCF-7 cells/well were seeded into black 96-well plates with clear bottom and incubated overnight at 37°C and 5% CO_2. Medium was exchanged for medium containing test compounds, followed by 3 h incubation at 37 °C and 5% CO_2. Subsequently, medium containing LysoTracker Red DND-99 and Hoechst-33342 was added, yielding final concentrations of 500 nM and 5 µg/µL, respectively. After 30 min incubation at 37 °C and 5% CO_2, cells were fixed with 4% paraformaldehyde in PBS for 5 min at room temperature, followed by three washing steps with PBS. Cells were imaged using an Axiovert 200 M automated screening microscope (Carl Zeiss, Germany) at 10x magnification. Image analysis was performed using

CellProfiler.[9] Cells were identified via Hoechst-33342 staining, LysoTracker Red DND-99 intensity was measured per cell, obtained values were averaged per well and normalized to solvent control (=100%).

Reagents

BODIPY 581/591 C11 (Thermo Fisher Scientific, #D3861), Calcein-AM (Thermo Fisher Scientific, #C1430), Cell Rox Deep Red (Thermo Fisher Scientific, #C10422), CM-H2-DCFDA (Thermo Fisher Scientific, #C6827), Lysotracker-DR (Thermo Fisher Scientific, #L12492), FITC-Dextran (1mg/ mL for 1h, Sigma-Aldrich, #FD10S), Lysotracker DND-26 (Thermo Fisher Scientific, #L7535), MitoSOX (Thermo Fisher Scientific, #M36008) Trypan blue solution 0.4% (EVE™ Cell Counting Slides, NanoEnTek kit, #E1020), Turn-on cytosolic Fe^{2+} probe (1 μM for 1 h , Rhonox-1),[10] Turn-on lysosomal Fe^{2+} probe (1 μM for 1 h , Rhonox-M),[11] Turn-off mitochondrial Fe^{2+} probe (1 μM for 1 h).[12] All commercial probes were used according to the manufacturer's protocol.

Cell viability

Cell viability was carried out by plating 1000 cells/well in 96-well plates using CellTiter-Blue® viability assay according to the manufacturer's protocol. Cells were treated as indicated for 72 h. CellTiter-Blue® reagent (G8081, Promega) was added after 72 h treatment and cells were incubated for 1-2 h before recording fluorescence intensities (ex. 560/20 nm; em. 590/10 nm) using a Perkin Elmer Wallac 1420 Victor2 Microplate Reader. Trypan blue exclusion and cell viability measurements were performed using an EVE Automated Cell Counter (NanoEnTek, #E1000) and EVE™ Cell Counting Slides. 10 μL of cell suspension were mixed with 10 μL of 0.4% Trypan solution and a final volume of 10 μL of this mixture used to count cells and assess viability according to the manufacturer's protocol.

Cell imaging (Figure 3 and Supporting Information Figure 3)

For immunofluorescence, cells were blocked with 2% BSA, 0.2% Tween-20/PBS (blocking buffer) for 20 min at RT. Cover-slips were incubated with 50 to 100 μL of diluted primary antibodies in blocking buffer 1 h at RT. Cover-slips were then washed three times with blocking buffer and incubated as described above with the appropriate secondary antibodies for 1 h. Cover-slips were washed three times with PBS and mounted using Vectashield Mounting Medium with DAPI (H-1200, VECTOR Laboratories). Fluorescence images were

acquired using a Deltavision real-time microscope (Applied Precision). 60×/1.4NA and 100×/1.4NA objectives were used for 2D and 3D acquisitions that were deconvoluted with SoftWorx (Ratio conservative - 15 iterations, Applied Precision) and processed with ImageJ®.

Flow cytometry

Metal-specific probes were synthesized as previously described. Cells were treated as indicated in the figures. For mitochondrial copper and iron probes, cells were incubated with the relevant probe prior to being analyzed by flow cytometry. Cells were trypsinized (TrypLE™ Express Enzyme, Life Technologies, 12605010) and washed twice with ice cold PBS. For antibodies, cells were suspended in ice cold PBS containing 2% FBS and 1 mM EDTA (incubation buffer) and incubated for 20 min at 4 °C with the relevant antibody. Cells were then washed twice with ice cold PBS and suspended in incubation buffer prior to being analyzed by flow cytometry. For each condition, at least 100.000 cells were counted. Data were recorded on a BD Accuri™ C6 (BD Biosciences) and processed using Cell Quest (BD Biosciences) and FlowJo (FLOWJO, LLC) software.

Cellular levels of copper and iron

Cells were incubated with the relevant probes as described in Reagents section. Cells were then analyzed either by fluorescence microscopy or flow cytometry as described in Cell Imaging and Flow Cytometry sections.

Lysosomal membrane permeabilization assay

Cells were plated 24 h prior to the experiments and then treated as indicated. Then, cells were treated with Dextran-FITC at 1 mg/ mL for 1 h in cell medium. Cells where then fixed with formaldehyde (2% in PBS, 12 min) and analyzed by microscopy.

ROS levels

ROS were measured using appropriate according to the manufacturer's protocol. In brief, cells were treated for the indicated time with the relevant dye, trypsinized, washed and resuspended in ice cold PBS containing 2% FBS and 1 mM EDTA prior to being analyzed by flow

cytometry. For immunofluorescence, live cells were treated with the appropriate dye for 1 h prior to fixation.

Statistical Analysis

All results are presented as means with their standard deviation (SD), unless stated otherwise. Data are representative of at least three independent biological replicates, unless stated otherwise. Statistical significance (p values) was determined by Student's t tests (two-tailed unpaired) using Prism6 software (GraphPad). $*p \leq 0.05$, $**p \leq 0.01$, $***p \leq 0.001$, $****p \leq 0.0001$, NS = non-significant.

References

[1] M. Jaric, B. A. Haag, S. M. Manolikakes, P. Knochel, *Org. Lett.* **2011**, *13*, 2306-2309.
[2] D. R. Hutchison, V. V. Khau, M. J. Martinelli, N. K. Nayyar, B. C. Peterson, K. A. Sullivan, *Org. Synth.* **2003**, 223-223.
[3] J. Cox, M. Mann, *Nat. Biotech.* **2008**, *26*, 1367.
[4] S. Tyanova, J. Cox, in *Cancer Systems Biology: Methods and Protocols* (Ed.: L. von Stechow), Springer New York, New York, NY, **2018**, pp. 133-148.
[5] M.-A. Bray, S. Singh, H. Han, C. T. Davis, B. Borgeson, C. Hartland, M. Kost-Alimova, S. M. Gustafsdottir, C. C. Gibson, A. E. Carpenter, *Nat. Prot.* **2016**, *11*, 1757.
[6] M. H. Woehrmann, W. M. Bray, J. K. Durbin, S. C. Nisam, A. K. Michael, E. Glassey, J. M. Stuart, R. S. Lokey, *Mol. BioSystems* **2013**, *9*, 2604-2617.
[7] L. Laraia, A. Friese, D. P. Corkery, G. Konstantinidis, N. Erwin, W. Hofer, H. Karatas, L. Klewer, A. Brockmeyer, M. Metz, B. Schölermann, M. Dwivedi, L. Li, P. Rios-Munoz, M. Köhn, R. Winter, I. R. Vetter, S. Ziegler, P. Janning, Y.-W. Wu, H. Waldmann, *Nat. Chem. Biol.* **2019**, *15*, 710-720.
[8] T. Pinkert, D. Furkert, T. Korte, A. Herrmann, C. Arenz, *Angew. Chem. Int. Ed.* **2017**, *56*, 2790-2794.
[9] A. E. Carpenter, T. R. Jones, M. R. Lamprecht, C. Clarke, I. H. Kang, O. Friman, D. A. Guertin, J. H. Chang, R. A. Lindquist, J. Moffat, P. Golland, D. M. Sabatini, *Genome Biol.* **2006**, *7*, R100.
[10] T. Hirayama, K. Okuda, H. Nagasawa, *Chem. Sci.* **2013**, *4*, 1250-1256.
[11] M. Niwa, T. Hirayama, K. Okuda, H. Nagasawa, *Org. Biomol. Chem.* **2014**, *12*, 6590-6597.
[12] F. Petrat, D. Weisheit, M. Lensen, H. de Groot, R. Sustmann, U. Rauen, *Biochem. J.* **2002**, *362*, 137.
